# Quality of Life Following Receipt of Adjuvant Chemotherapy With and Without Bevacizumab in Patients With Lymph Node–Positive and High-Risk Lymph Node–Negative Breast Cancer

**DOI:** 10.1001/jamanetworkopen.2022.0254

**Published:** 2022-02-28

**Authors:** Shoshana M. Rosenberg, Anne O’Neill, Karen Sepucha, Kathy D. Miller, Chau T. Dang, Donald W. Northfelt, George W. Sledge, Bryan P. Schneider, Ann H. Partridge

**Affiliations:** 1 Dana-Farber Cancer Institute, Boston, Massachusetts; 2 ECOG-ACRIN Biostatistics Center, Boston, Massachusetts; 3 Massachusetts General Hospital, Boston; 4 Indiana University, Indianapolis; 5 Memorial Sloan Kettering Cancer Center, New York, New York; 6 Mayo Clinic, Phoenix, Arizona; 7 Stanford University, Stanford, California

## Abstract

**Importance:**

Breast cancer treatment can impact not only short-term health but may also affect longer-term quality of life (QOL).

**Objective:**

To describe and evaluate factors associated with diminished QOL following completion of active treatment.

**Design, Setting, and Participants:**

This was a secondary analysis of a randomized clinical trial included patients with lymph node–positive or high-risk lymph node–negative breast cancer who had undergone definitive surgery and were enrolled in ECOG-ACRIN E5103, a multisite phase 3 trial. A survey was administered 18 months after enrollment to patients enrolled between January and June 2010. Final analysis of the data took place from March to December 2021.

**Interventions:**

Patients received adjuvant doxorubicin, cyclophosphamide, and paclitaxel with either bevacizumab or placebo.

**Main Outcomes and Measures:**

QOL and health status assessed with the EuroQol 5-Dimension 3-Levels (EQ-5D-3L), EQ–visual analog scale (EQ-VAS), and the Functional Assessment of Cancer Therapy–Breast Cancer, with arm subscale (FACT-B+4). Groups were compared by Fisher exact test, Wilcoxon rank sum, or Kruskal-Wallis test. Multivariable linear regression was used to assess factors independently associated with FACT-B scores.

**Results:**

Data at 18 months were available from 455 of 519 patients (87.7%) enrolled in the trial. Median (range) age at enrollment was 52 (25-76) years. No differences in QOL (median [range] FACT-B scores: group A, 123 [67-146]; group B, 114 [54-148]; group C, 117 [42-148]; *P* = .23) or health status (median [range] EQ-5D-3L index scores: group A, 0.83 [0.28-1.00]; group B, 0.83 [0.20-1.00]; group C, 0.83 [0.17-1.00], *P* = .80; median EQ-VAS: group A, 85 [20-100]; group B, 85 [0-100]; group C, 85 [0-100]; *P* = .79) were observed across treatment groups; results for subsequent analyses were therefore reported irrespective of primary treatment. Overall, half of patients (258 of 444 [58%]) reported at least some pain or discomfort; 170 (38%) reported symptoms of anxiety or depression. In multivariable analyses, mastectomy with radiation (vs breast conserving surgery) and Asian, Black, or American Indian or Alaska Native race (vs White race) were associated with lower QOL (mastectomy with radiation: coefficient: −5.5; 95% CI, −10.1 to −0.9; Asian, Black, or American Indian or Alaska Native race: coefficient: −7.3; 95% CI, −13.2, −1.4).

**Conclusions and Relevance:**

In this study, the addition of bevacizumab to chemotherapy was not negatively associated with QOL at 18 months. A substantial proportion of participants reported problems related to pain or discomfort and anxiety or depression, demonstrating persistent consequences for physical and psychosocial well-being in this heavily treated population. Many problems reported are amenable to intervention, underscoring the need for timely referral to supportive resources, especially for women of color and those who have more extensive local therapy.

**Trial Registration:**

ClinicalTrials.gov Identifier: NCT00433511

## Key Points

**Question:**

Who is at risk of diminished quality of life (QOL) in extended follow-up of breast cancer survivors treated with intensive systemic treatment?

**Findings:**

Among 455 participants included in this secondary analysis of a phase 3 randomized clinical trial, adding bevacizumab to chemotherapy was not negatively associated with QOL at 18 months. A substantial proportion of patients reported problems related to pain or discomfort and anxiety or depression; receiving mastectomy with radiation as well as identifying as Asian, Black, or American Indian or Alaska Native were associated with lower QOL.

**Meaning:**

These findings suggest that early intervention and referral to supportive resources is critical for survivors at risk of experiencing diminished QOL, especially among individuals from underrepresented racial and ethnic groups and those undergoing extensive local therapy.

## Teaser

This secondary analysis of a phase 3 randomized clinical trial evaluates factors associated with diminished quality of life among individuals who have completed active treatment for breast cancer.

## Introduction

It is well established that breast cancer treatment can affect not only short-term health outcomes but also longer-term health-related and psychosocial quality of life (QOL).^1,2,3^ While clinical trials routinely document adverse effects during treatment, incorporation of patient-reported outcomes (PROs) into trial assessments increases our understanding of the acute and sustained consequences of treatment on physical and psychosocial well-being.

The ECOG-ACRIN Cancer Research Group (ECOG-ACRIN) E5103 multisite trial randomly assigned patients to chemotherapy with and without bevacizumab between November 2007 and February 2011, and a large consecutively enrolled subset were surveyed regarding QOL and health status in follow-up.^4^ The primary objective of the current analysis was to compare QOL and health status among treatment arms in the parent trial at 18 months following enrollment. As secondary objectives, we explored differences in QOL and health status by local therapy as well as demographic, clinical, and treatment factors associated with of worse QOL at 18 months after enrollment. Collection of PROs beyond the completion of active treatment from patients, all of whom received chemotherapy and nearly 80% of whom received bevacizumab, allowed for the evaluation of QOL in the setting of targeted therapy, and provides important information regarding the experience of breast cancer survivors with higher-risk disease in extended follow-up.

## Methods

### Study Design

This study was a secondary analysis assessing quality of life objectives in ECOG-ACRIN E5103, a double-blind phase 3 trial open to enrollment between November 2007 and February 2011.^4^ The trial protocol appears in Supplement 1. Patients were randomly assigned 1:2:2 to 1 of 3 study arms: adjuvant doxorubicin, cyclophosphamide followed by paclitaxel with placebo (group A); adjuvant doxorubicin, cyclophosphamide with bevacizumab followed by paclitaxel with bevacizumab (group B); or adjuvant doxorubicin, cyclophosphamide with bevacizumab followed by paclitaxel with bevacizumab and bevacizumab monotherapy (group C). The decision-making–QOL (DM-QOL) component of the study was open to all patients who enrolled onto E5103 between January and June 2010. The National Cancer Institute central institutional review board (IRB) approved the protocol as the IRB of record for a subset of institutions; the remaining sites used their own individual IRBs. Written informed consent was obtained from all participants. The Consolidated Standards of Reporting Trials (CONSORT) reporting guidelines for randomized studies were followed.

### Study Procedures

PROs were assessed via telephone-based surveys administered at baseline (enrollment), 6 weeks (midtreatment), 18 or 22 weeks (after unblinding), 12 months (completion of all treatment), and 18 months post enrollment. Given the focus on posttreatment QOL, the current analysis includes data collected at the final 18-month time point.

At baseline, patients were provided a copy of the surveys to complete in clinic and were then asked to take them home. A research interviewer then called and collected the PROs over the telephone. Patients were mailed a copy of the follow-up surveys 7 to 10 days prior to their scheduled interview, with a request to complete them prior to the arranged time of their interview.

### Outcome Measures

#### Overall QOL

Overall QOL was assessed with the Functional Assessment of Cancer Therapy Scale–Breast Cancer with arm subscale (FACT-B+4). The FACT-B includes the FACT-General (FACT-G), version 4.0, developed by Cella and colleagues^6^ as an overall cancer-specific QOL measure, with a 9-item subscale concerning breast cancer–specific issues.^5^ The FACT-G consists of a 27 item core QOL measure grouped into 4 subscale domains: physical well-being, social and/or family well-being, emotional well-being, and functional well-being.^6^ Almost all items are rated on a 5-item Likert scale, from 0, indicating not at all, to 4, indicating very much, and ask individuals about how they have felt during the past 7 days. The breast cancer module consists primarily of physical symptoms, body image, and sexual issues. The arm subscale includes 4 additional items plus 1 item from the breast cancer module to assess the impact of arm morbidity on patients following surgery. FACT-B scores range from 0 to 148; scores for the physical, social, and functional subscales range from 0 to 28. For the emotional subscale, breast cancer–specific subscales, and arm subscales, scores range from 0 to 24, 0 to 40, and 0 to 20, respectively. Higher scores represent better QOL. A difference of 7 to 8 points on the FACT-B score has been determined to be a minimally important difference (MID), with MIDs for individual subscale scores ranging from 1 to 3 points.^7,8^

#### Health Status

The EuroQol 5-Dimensions 3-Levels (EQ-5D-3L) is a measure of health status for use in evaluating health and health care.^9^ Descriptively, the EQ-5D consists of 5 dimensions: mobility, self-care, usual activity, pain or discomfort, and anxiety or depression. Each dimension has 3 levels designated as no problem, some problem, or extreme problem, with patients selecting their current level of function for that day on each dimension. Three different health status metrics were evaluated: (1) health status evaluation, representing the proportion reporting no problems vs some or extreme problems within each of the 5 dimensions; (2) index score, derived from the 5 dimensions, ranging from −0.11 (death) to 1 (perfect health) with an MID of 0.06 points; and (3) visual analog score (VAS), ranging from 0 (worst imaginable) to 100 (best imaginable), with an MID of 7 points.^10^

### Other Covariates

Sociodemographic characteristics, such as marital and education status, were collected using a modified version of the Cancer and Leukemia Group B (CALGB) background information form.^11^ Age, race, ethnicity, menopausal status, local therapy information (surgery, radiation), tumor pathologic features (estrogen [ER] and progesterone receptor [PR] status, tumor size, nodal status) at study enrollment were collected on trial case report forms. Race and ethnicity were collected at the time of online registration to the parent trial on case report forms. Race and ethnicity were included as covariates to explore the association of sociodemographic characteristics and clinical factors with QOL.

### Statistical Analysis

Final analysis of the data took place from March to December 2021. The Fisher exact test (categorical variables), Wilcoxon rank sum test (continuous variables), and Kruskal-Wallis test (if more than 2 groups) were used to test across or between groups. A 2-sided *P* <.05 was considered statistically significant. Linear regression was used to assess the association of sociodemographic (eg, age, race, education status) and clinical (eg, surgery or radiation, ER and PR status, tumor size, nodal status) variables with QOL (FACT-B score at 18 months). Individuals identifying as Asian, Black, or American Indian or Alaska Native were combined into 1 group for analytic purposes. The multivariable model with the lowest Akaike information criteria (AIC) was selected. Analyses were conducted using SAS version 9.4 (SAS Institute).

## Results

Of 4836 patients who started protocol treatment on E5103, 571 patients started treatment between January and June 2010. Of these, 519 remained in the study at the 18-month point and 465 answered at least 1 question on the 18-month surveys; additionally, 10 patients recurred prior to 18 months and were therefore excluded, leaving 455 patients (87.7%) in the analytic cohort (eFigure in Supplement 2). There were no significant differences in baseline and disease characteristics for the subset enrolled in the DM-QOL component and those in the parent study who began protocol treatment and were not included in this analytic cohort (eTable in Supplement 2).

### Study Participant Characteristics

Table 1 describes participant characteristics. Median (range) age at enrollment was 52 (25-76) years. Most participants were White (393 of 455 [86%]), non-Hispanic (403 of 426 [95%]), and married or partnered (302 of 424 [71%]). More than half (234 of 425 [55%]) had less than a college education. Regarding tumor pathologic features, nearly two-thirds (299 of 455 [66%]) had ER- and/or PR-positive tumors, 290 of 455 tumors (64%) were larger than 2 cm, and 330 (73%) were node positive.

**Table 1.** Patient Demographic and Clinical Characteristics

Characteristic	Patients, No. (%) (N = 455)
Age, median (range), y	51.9 (25.2-76.4)
Gender	
Female	452 (99)
Male	3 (<1)
Race	
White	393 (86)
Black	49 (11)
Asian	12 (2.6)
American Indian or Alaska Native	1 (<1)
Ethnicity	
Hispanic	23 (5)
Non-Hispanic	403 (95)
Missing or unknown	29
Marital status	
Single, separated, divorced, or widowed	122 (28)
Married or partnered	302 (71)
Missing or unknown	31
Education status	
No college	234 (55)
College or higher	191 (45)
Missing or unknown	30
Menopausal status	
Postmenopausal	250 (56)
Premenopausal or perimenopausal	198 (44)
Missing, unknown, or male patient	7
ER/PR status	
ER and PR negative	156 (34)
ER and PR positive	299 (66)
Tumor size, cm	
≤2	165 (36)
>2	290 (64)
Nodal status	
Negative	125 (27)
Positive	330 (73)
Histologic grade	
I	39 (9)
II	160 (36)
III	245 (55)
Missing or unknown	11
Surgery	
BCS^a^	195 (43)
Mastectomy	
Without radiation	86 (19)
With radiation	171 (38)
Missing	3
Axillary surgery	
SLNB	86 (19)
ALND	108 (24)
ALND and SLNB	261 (57)

Abbreviations: ALND, axillary lymph node dissection; BCS, breast conserving surgery; ER, estrogen receptor; PR, progesterone receptor; SLNB, sentinel lymph node biopsy.

^a^ Includes patients who received BCS with whole breast radiation (n = 184), BCS with partial breast irradiation (n = 3), and BCS without radiation (n = 8).

A total of 257 women (57%) underwent mastectomy; of these, approximately two-thirds (171 [66%]) received postmastectomy radiation therapy (PMRT). Among the 195 women who had breast conserving surgery (BCS), 184 (94%) received whole breast radiation therapy; 3 women (2%) had partial breast irradiation and 8 (4%) did not receive any type of radiation. All women who had BCS were analyzed as a single group, as small numbers precluded any evaluation of women who had BCS without radiation separately from those who did. Regarding axillary surgery, 86 women (19%) had a sentinel lymph node biopsy (SLNB) only, 108 (24%) had an axillary lymph node dissection (ALND) only, and 261 (57%) had both an SLNB and an ALND.

### QOL and Health Status Across Treatment Groups

There were no statistically or clinically significant differences in QOL and health status scores observed across treatment groups for the FACT-B (median [range] score: group A, 123 [67-146]; group B, 114 [54-148]; group C, 117 [42-148]; *P* = .23), the EQ-5D-3L Index Score (median [range] score: group A, 0.83 [0.28-1.00]; group B, 0.83 [0.20-1.00]; group C, 0.83 [0.17-1.00]; *P* = .80), and the EQ-VAS (median [range] score: group A, 85 [20-100]; group B, 85 [0-100]; group C, 85 [0-100]; *P* = .79) (Table 2). Scores for individual FACT-B domains were also similar across treatment group, except for the emotional well-being domain, in which statistical significance was achieved but scores were not clinically different (median [range]: group A, 21.0 [8.0-24.0]; group B, 20.0 [6.0-24.0]; group C, 21.0 [2.0-24.0]; *P* = .03). Across treatment groups, while physical well-being and emotional well-being scores were similar to the US female general population references^12^ of 23.3 (median physical well-being) and 21.0 (median emotional well-being), observed functional and social and family well-being scores were slightly higher in our sample than the US female general population references of 18.7 (median functional well-being) and 21.0 (median social well-being). Because there were no statistically or clinically significant differences across the treatment groups, results for the subsequent QOL analyses are reported irrespective of treatment received in the parent clinical trial.

**Table 2.** Quality of Life and Health Status by Study Group

Scale	Median score (range)^a^			*P* value^b^
	Group A, chemotherapy with placebo	Group B, chemotherapy with bevacizumab	Group C, chemotherapy with bevacizumab (extended)	
FACT-B	123.0 (67.2-146.0)	113.5 (54.0-148.0)	117.0 (42.3-148.0)	.23
FACT-G individual subscale domains				
Physical	24.0 (9.0-28.0)	24.0 (0-28.0)	24.0 (1.0-28.0)	.97
Social and family	24.8 (0-28.0)	24.2 (5.6-28.0)	24.0 (4.0-28.0)	.47
Emotional	21.0 (8-24.0)	20.0 (6.0-24.0)	21.0 (2.0-24.0)	.03
Functional	23.0 (6.0-28.0)	22.0 (4.0-28.0)	22.0 (4.0-28.0)	.62
Breast cancer–specific	29.0 (12.0-40.0)	27.0 (9.0-40.0)	28.0 (7.0-40.0)	.20
Arm subscale	17.0 (4.0-20.0)	16.0 (0-20.0)	16.0 (0-20.0)	.76
EQ-5D-3L Index	0.83 (0.28-1.00)	0.83 (0.20-1.00)	0.83 (0.17-1.00)	.80
EQ-VAS	85.0 (20.0-100)	85.0 (0-100)	85.0 (0-100)	.79

Abbreviations: EQ-5D-3L, EuroQol 5–Dimension 3–Level; EQ-VAS, EuroQol visual analogue scale; FACT-B, Functional Assessment of Cancer Therapy Scale–Breast Cancer; FACT-G, Functional Assessment of Cancer Therapy Scale–General.

^a^ Higher scores indicate better quality of life. The maximum score for FACT-B is 148; FACT-G physical, social, and functional subscale, 28; FACT-G emotional subscale, 24; breast cancer–specific subscale, 40, arm subscale, 20.

^b^ Kruskal-Wallis test.

### Comparison of QOL and Health Status Between Local Therapy Strategies

FACT-B, EQ-5D-3L, and EQ-VAS scores by local therapy strategy are presented in Table 3. Compared with women who had BCS, women who had mastectomy with PMRT had statistically significantly lower median (range) FACT-B scores (120.0 [56.2-148.0] vs 113.5 [42.3-148.0], *P* = .003), a 6.5 point difference approaching the MID threshold of 7 to 8 points. Regarding FACT-B individual domains, women who had mastectomy with PMRT, compared with those who had BCS, had lower median (range) arm subscale (15.0 [0-20.0] vs 17.0 [0-20.0]; *P* < .001) and breast cancer–specific (27.0 [7.0-40.0] vs 30.0 [9.0-40.0]; *P* < .001) scores, with between-group differences meeting the MID threshold (1-3 points). Median (range) emotional well-being scores (20.0 [2.0-24.0] vs 20.0 [9.0-24.0], *P* = .03) were statistically but not numerically or clinically different. Among women who had mastectomy, median (range) arm subscale scores were lower among those who received PMRT vs those who did not (15.0 [0-20.0] vs 17.0 [1.0-20.0]; *P* < .001). Median (range) EQ-VAS scores were also lower among women who had mastectomy with PMRT vs BCS (80.0 [0-100] vs 86.5 [30-100]; *P* = .003; MID, 7 points). EQ-5D-3L profile domains are shown in the Figure. Among all patients, more than half (258 of 444 [58%]) reported at least some pain or discomfort; 170 of 444 (38%) reported symptoms of anxiety or depression; and 135 of 444 (30%) reported at least some problems with usual activities. There were no statistically significant differences by local therapy strategy within each of the 5 EQ-5D-3L health status dimensions.

**Table 3.** Quality of Life and Health Status: BCS vs Mastectomy With and Without Radiation

Scale	Median score (range)^a^			*P* value^b^		
	BCS	Mastectomy		Mastectomy without PMRT vs BCS	Mastectomy with PMRT vs BCS	Mastectomy with vs without PMRT
		Without PMRT	With PMRT			
FACT-B	120.0 (56.2-148.0)	117.3 (53.3-146.0)	113.5 (42.3-148.0)	.26	.003	.23
FACT-G individual domains						
Physical	25.0 (0-28.0)	25.0 (2.8-28.0)	24.0 (1.0-28.0)	.65	.02	.11
Social and family	25.0 (0-28.0)	24.0 (4.7-28.0)	24.0 (8.0-28.0)	.49	.23	.66
Emotional	20.0 (9.0-24.0)	20.0 (7.0-24.0)	20.0 (2.0-24.0)	.44	.03	.32
Functional	23.0 (4.0-28.0)	22.0 (5.0-28.0)	21.0 (4.0-28.0)	.23	.06	.79
Breast cancer–specific	30.0 (9.0-40.0)	29.0 (7.0-40.0)	27.0 (7.0-40.0)	.06	<.001	.17
Arm subscale	17.0 (0-20.0)	17.0 (1-20.0)	15.0 (0-20.0)	.63	<.001	<.001
EQ-5D-3L Index	0.83 (0.24-1.00)	0.83 (0.20-1.00)	0.83 (0.17-1.00)	.16	.05	.89
EQ-VAS	86.5 (30.0-100)	85.0 (20.0-100)	80.0 (0-100)	.33	.003	.11

Abbreviations: BCS, breast conserving surgery; EQ-5D-3L, EuroQol 5–Dimension–3 Level; EQ-VAS, EuroQol visual analogue scale; FACT-B, Functional Assessment of Cancer Therapy Scale–Breast Cancer; FACT-G, Functional Assessment of Cancer Therapy Scale–General; PMRT, postmastectomy radiation.

^a^ Higher scores indicate better quality of life.

^b^ *P* values based on Wilcoxon rank sum test.

**Figure.**
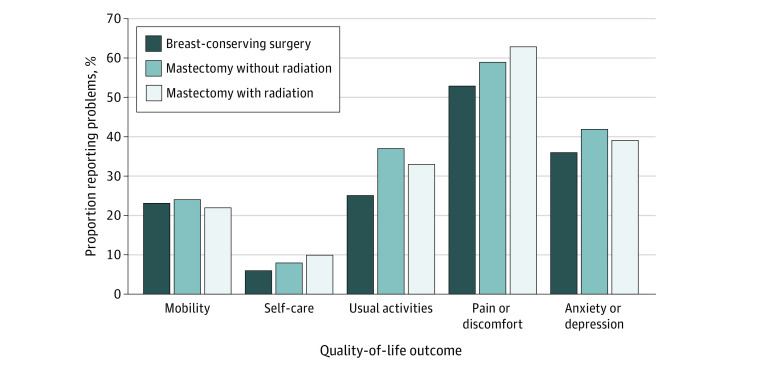
EuroQol 5-Dimension 3-Level Health Profile Evaluation by Local Therapy Strategy The 3 levels have 3 possible answers: (1) no problems, (2) some or moderate problems, and (3) problems; responses were then collapsed into no problems vs any problems (any problems including some or moderate problems and problems).

### Factors Associated With QOL

Modeling was used to assess the association of sociodemographic and clinical factors with QOL as measured by the FACT-B. Univariable and multivariable model results are presented in Table 4. The mean FACT-B score for Asian, Black, and American Indian or Alaska Native women was approximately 7 points lower than the mean FACT-B score for White women in both univariate (coefficient: −7.3, 95% CI, −13.2 to −1.3) and multivariable models (coefficient, −7.3; 95% CI, −13.2 to −1.4). The mean FACT-B score for women who had a mastectomy with PMRT was more than 6 points lower than the mean FACT-B score for women who had BCS in the univariate model (coefficient, −6.6; 95% CI, −11.1 to −2.1) and slightly attenuated in the multivariable model (coefficient, −5.5; 95% CI, −10.1 to −0.9). The mean FACT-B score for women with ER-positive tumors was 5 points lower than the mean FACT-B score for women with ER-negative tumors in univariable analyses (coefficient, −5.1; 95% CI, −9.4 to −0.9), although this effect was modestly attenuated in multivariable analyses. Age, a known prognostic factor, although not statistically significant, was retained by the model selection criteria (lowest AIC) in the final multivariable model. Education status, tumor size, and nodal status were not associated with QOL as assessed by the FACT-B in either univariable or multivariable analyses.

**Table 4.** Factors Associated With Total FACT-B Scores at 18 Months

Factor	Univariate model		Multivariable model^a^	
	Coefficient (95% CI)	*P* value	Coefficient (95% CI)	*P* value
Mastectomy				
Without PMRT vs BCS	−3.1 (−8.7 to 2.5)	.28	−2.9 (−8.6 to 2.6)	.29
With PMRT vs BCS	−6.6 (−11.1 to −2.1)	.004	−5.5 (−10.1 to −0.9)	.02
Age (years)	0.1 (−0.1 to 0.3)	.15	0.1 (−0.06 to 0.3)	.17
Asian, Black, or American Indian or Alaska Native vs White	−7.3 (−13.2 to −1.3)	.02	−7.3 (−13.2 to −1.4)	.02
No college education vs college education^b^	−0.71 (−4.9 to 3.4)	.74	NA	NA
ER/PR positive vs negative	−5.1 (−9.4 to −0.9)	.02	−4.4 (−8.8 to 0.07)	.05
Tumor size >2 cm vs ≤2 cm	−3.7 (−7.9 to 0.5)	.09	NA	NA
Nodal status positive vs negative	−3.3 (−7.9 to 1.3)	.15	NA	NA

Abbreviations: BCS, breast conserving surgery; ER, estrogen receptor; FACT-B, Functional Assessment of Cancer Therapy Scale–Breast Cancer; NA, not applicable; PR, progesterone receptor; PMRT, postmastectomy radiation.

^a^ Based on linear regression model with lowest Akaike information criterion.

^b^ Education status was missing or unknown for 30 patients and was not assessed in the multivariable model.

## Discussion

At 18 months post enrollment, the addition of bevacizumab to an adjuvant chemotherapy regimen was not negatively associated with QOL, including among those who received extended duration therapy. Additionally, by 18 months, QOL scores were similar to US general population references. However, in post hoc analyses, while there did not appear to be differences between women who had BCS and women who had mastectomy without PMRT, women who had mastectomy and PMRT had lower FACT-B and EQ-VAS scores compared with women who had BCS. Additionally, among those who had mastectomy, those who received PMRT had lower FACT-B arm subscale scores than those who did not have PMRT, suggesting decreased QOL attributable to greater arm morbidity associated with the addition of radiation therapy.

Many women may not be monitored as closely by their clinicians when active treatment ends, yet we observed that a substantial symptom burden persists in the months following the completion of active treatment. Overall, in our study, 58% of all patients reported problems with pain and discomfort. Prior studies have documented substantial levels of chronic pain reported by women after breast cancer surgery, with 1 systematic review reporting a median frequency of persistent pain of 37.5%.^13,14,15^ Established treatment-related risk factors for experiencing chronic postsurgical pain include radiation, receipt of ALND, and receipt of chemotherapy.^16^ Given that all women in our study were treated with chemotherapy, most had either ALND or SLNB with ALND, and two-thirds of women who had mastectomy received PMRT, reports of persistent pain and discomfort at 18 months after diagnosis should underscore the need for effective pain amelioration strategies into longer-term survivorship, particularly among patient populations who undergo heavy treatment.

Additionally, we found that women who identified as Asian, Black, or American Indian or Alaska Native appeared to be at increased risk for experiencing posttreatment detriments in QOL. Findings from prior studies that have explored differences in QOL by race have been mixed. While mental, social, and emotional well-being have generally been found to be better in Black breast cancer survivors relative to White survivors, physical and functional domains of QOL have more often been worse, although these differences have usually been modest and not always of clinical significance.^17,18,19^ Black women are less likely to undergo SLNB and are more likely to develop lymphedema than White women.^20^ Among women enrolled in a recent lymphedema prevention trial, African American women had greater QOL detriments as a consequence of lymphedema compared with White non-Hispanic women or women from other racial and ethnic backgrounds.^21^ Given the well-documented association between lymphedema and experiencing pain as well as decreased QOL,^16,22^ prevention and early treatment of lymphedema among women at increased risk, including Black and African American women, should be a priority to enhance well-being in survivorship.

The American Cancer Society/American Society of Clinical Oncology (ACS/ASCO) Breast Cancer Survivorship Care Guidelines comprise comprehensive recommendations that address both physical and psychosocial long-term and late effects of treatment, including the prevention and treatment of lymphedema, musculoskeletal symptoms, pain, neuropathy, and fatigue.^23^ Notably, 38% of women in our study reported at least some problems related to anxiety or depression. Given that a substantial proportion of breast cancer survivors experience psychological sequelae, effectively implementing the ACS/ASCO recommendations regarding evaluation of distress, depression, and anxiety (with a more in-depth evaluation for those deemed to have higher risk of depression) is critical to ensuring that those who need it are provided with appropriate psychological support.^23^

### Limitations

Our findings should be interpreted in the context of some limitations. Our study was not designed to analyze differences by surgery or radiation, thus comparisons between BCS and mastectomy (with and without radiation) were exploratory and were not adjusted for multiple comparisons. While we adjusted for potential factors known to be associated with QOL in the regression model, we cannot exclude the possibility that differences in preoperative factors, including QOL, may be associated with surgical preferences as well as postoperative QOL. Furthermore, we did not have data available regarding how many women underwent postmastectomy reconstruction which may also factor into longer-term QOL. Finally, although we observed an association between race and QOL, our study population was predominantly White (86%). Ensuring that Black patients as well as patients from other racial and ethnic backgrounds who historically have been underrepresented in clinical trials are offered the opportunity to participate in clinical trials should be a priority. These efforts should extend to trials that include QOL end points, where diversity in the patient-reported experience is essential to capture.

## Conclusions

This secondary analysis of a randomized clinical trial found no significant differences in QOL between study groups, with a substantial proportion of patients reporting problems related to pain or discomfort and anxiety or depression. Attention to both physical and psychosocial well-being is essential both during and after completion of active breast cancer treatment. Many of the problems that were reported by survivors in our study are amenable to intervention, underscoring the need for timely referral to supportive resources, especially for underserved populations. Routine monitoring of symptoms and functional status is warranted to identify and ameliorate symptoms early that may adversely affect QOL as well as to improve pain and optimize and sustain psychological health and physical functioning in breast cancer survivors during treatment and into survivorship.

## Notes

Supplement 1.
Trial Protocol

Supplement 2. **eFigure. **E5103 Decision-making–Quality of Life Flowchart **eTable. **Comparison of Baseline Characteristics Between Those Included and Not Included in the QOL Analysis Relative to the Parent Trial

Supplement 3.
Data Sharing Statement
