# Supplementary material for: Quality of Life Following Receipt of Adjuvant Chemotherapy With and Without Bevacizumab in Patients With Lymph Node–Positive and High-Risk Lymph Node–Negative Breast Cancer
Source: JAMA Netw Open. 2022 Feb 28;5(2):e220254. doi: 10.1001/jamanetworkopen.2022.0254 (PMC8886546; doi:10.1001/jamanetworkopen.2022.0254)
Supplement: Supplement 1. — Trial Protocol [file jamanetwopen-e220254-s001.pdf]

E5103

Rev. 5/14, 7/14

This is an FDA Registration Trial

A Double-Blind Phase III Trial of Doxorubicin and Cyclophosphamide followed by Paclitaxel with Bevacizumab or Placebo in Patients with Lymph Node Positive and High Risk Lymph Node Negative Breast Cancer

STUDY CHAIR:	Kathy D. Miller, M.D.
STUDY CO-CHAIR:	Ramona Swaby, M.D.
RADIATION THERAPY CO-CHAIR:	Larry Solin, M.D.
STUDY STATISTICIAN:	Anne O'Neill, M.S.
COMMITTEE CHAIR:	George W. Sledge, Jr., M.D.
COMMUNITY CO-CHAIR:	Robin Zon, M.D.
NCCTG CO-CHAIR:	Donald W. Northfelt, M.D.
CALGB CO-CHAIR:	Chau T. Dang, M.D.
DEVELOPMENTAL THERAPEUTICS CO-CHAIR:	Bryan P. Schneider, M.D.
QOL CHAIR:	Ann Partridge, M.D., M.P.H.

Rev. 10/09

Version Date: March 23, 2018
NCI Update Date: July 2, 2014

Rev. 12/11, 5/14, 7/14, 10/14

Bevacizumab (NSC 704865, IND #113919) Supplied by the NCI

STUDY PARTICIPANTS

Cancer Trials Support Unit (CTSU)*
ALLIANCE / Alliance for Clinical Trials in Oncology
SWOG / SWOG
NRG / NRG Oncology

ACTIVATION DATE

November 2, 2007
Addendum #1 – Incorporated prior to activation
Update #1 – Incorporated prior to activation
Update #2 – 12/07
Addendum #2 – 4/08
Update #3 – 5/08
Addendum #3- 7/09
Update #4 – 7/09
Addendum #4 – 10/09
Addendum #5 – 12/09
Addendum #6 – 7/10
Addendum #7 – 9/10
Addendum #8 – 1/11
Update #5 – 1/11
Update #6 – 9/11
Addendum #9 – 12/11
Update #7 – 1/12
Addendum #10 – 2/13
Update #8 – 3/13
Update #9 – 10/13
Addendum #11 – 5/14
Update #10 – 7/14
Addendum #12 – 10/14
Addendum #13 – 1/15
Addendum #14

Rev. 3/13

Table of Contents

Schema – classical (every 3 week) AC 8
Schema – dose dense (every 2 week) AC 9
1. Introduction 10
1.1 Angiogenesis in Breast Cancer 10
1.2 Vascular Endothelial Growth Factor 11
1.3 Bevacizumab 11
1.4 Bevacizumab in Breast Cancer 14
1.5 Selection of Adjuvant Chemotherapy 15
1.6 Gender and Ethnicities Statement 16
1.7 Predictive Biomarkers for Bevacizumab 16
1.8 Pharmacogenetics/Pharmacogenomics Data Analysis 16
2. Objectives 17
2.1 Primary Objective 17
2.2 Secondary Objectives 17
2.3 Quality of Life Objectives 17
2.4 Determinants of Late Relapse Ancillary Study (EL112LAB) 17
3. Selection of Patients 18
3.1 STEP 1: Registration to Arm A, B, C 18
3.2 STEP 2: Unblinding and Re-registration to Arm D 23
3.3 Step 3: Registration to Ancillary Study EL112LAB 23
4. Randomization Procedures 24
4.1 STEP 1: Registration to Arm A, Arm B or Arm C 26
4.2 Eligibility Verification 27
4.3 Stratification Factors 27
4.4 Additional Requirements 27
4.5 Instructions for Patients who Do Not Start Assigned Protocol Treatment28
4.6 Unblinding Procedures 28
4.7 STEP 2: Re-registration to Arm D for Unblinded Patients on Arm C 28
4.8 Emergency Unblinding 29
4.9 Reimbursement Guidelines for MUGA/ECHO scans, EKG and Urine Protein Studies 29
4.10 Guidelines for Technetium-99 Shortages 30
4.11 STEP 3: Registration to Ancillary Study EL112LAB 31
5. Treatment Plan 34
5.1 Administration Schedule – Classical Every 3 Week AC 34
5.2 Administration Schedule – Dose Dense Every 2 Week AC 41
5.3 Adverse Event Reporting Requirements 48
5.4 Comprehensive Adverse Events and Potential Risks list (CAEPR) for Bevacizumab (rhuMAb VEGF, NSC 704865) 56
5.5 Dose Modifications 62
5.6 Supportive Care 68

5.7 Duration of Therapy 69
5.8 Duration of Follow-up 69
6. Measurement of Effect 70
6.1 Local, Regional Recurrence 70
6.2 Distant Recurrence 70
6.3 Invasive Disease-Free Survival 70
6.4 Survival 70
6.5 Evidence of Recurrence 70
6.6 Quality of Life Measurement 70
7. Study Parameters 75
7.1 Therapeutic Parameters 75
7.2 Biological Sample Submissions 80
8. Drug Formulation and Procurement 82
8.1 Bevacizumab (NSC 704865, IND 113919) 82
8.2 Doxorubicin 91
8.3 Cyclophosphamide 93
8.4 Paclitaxel 97
8.5 Filgrastim 100
8.6 Pegfilgrastim 102
8.7 Epirubicin 104
9. Statistical Considerations 109
9.1 Primary and Secondary Efficacy Evaluations 109
9.2 Safety Monitoring 111
9.3 Gender and Ethnicity 112
9.4 QOL Analysis Plan 113
9.5 Study Monitoring 113
9.6 Pharmacogenetics/Pharmacogenomics Data Analysis 114
10. Correlative Studies 115
10.1 Sample Submission Schedule 115
10.2 Sample Preparation Guidelines 115
10.3 Shipping Procedures 116
10.4 ECOG-ACRIN Sample Tracking System 117
10.5 Banking 118
10.6 Sample Inventory Submission Guidelines 118
11. Records to Be Kept 119
11.1 Records Retention 119

12. Patient Consent and Peer Judgment 119
13. References 120
Appendix I Suggested Patient Consent Form [Deleted in Update #8] 128
Appendix I-A EL112LAB: North American Breast Cancer Groups Biospecimen Bank for Determinants of Late Relapse in Operable Breast Cancer Suggested Patient Consent Form [Deleted in Update #8] 129
Appendix II Pathology Submission Guidelines 130
Appendix III Patient Thank You Letter 135
Appendix IV Evaluation of Cardiac Toxicity 136
Appendix V Procedure for Obtaining a Urine Protein/Creatinine Ratio 137
Appendix VI Breast Cancer Clinical Trial Announcement 138
Appendix VII Cancer Trials Support Unit (CTSU) Participant Procedures 140
Appendix VIII E5103 Shipment Notification Form – REMOVED 144
Appendix IX Cooperative Research and Development Agreement (CRADA) 145
Appendix X NYHA Classification and Peripheral Vascular Disease Grading 147
Appendix XI E5103 Cycle 8 Unblinding Instructions 148
Appendix XII Substitute W-9 Tax Form 150
Appendix XIII Important Tips for this FDA Registration Trial 152
Appendix XIV E5103 and EL112LAB Collection and Shipping Kit Order Form 153
Appendix XV Ancillary Study EL112LAB 155

STUDY CHAIR

Kathy D. Miller, M.D.
Indiana Cancer Pavilion
535 Barnhill Dr., RT-473
Rev. 1/12 Indianapolis, IN 46202-5289
Phone: (317) 944-3553
Fax: (317) 274-3646
Email: kathmill@iupui.edu

STUDY CO-CHAIR

Ramona Swaby, M.D.
Fox Chase Cancer Center
333 Cotman Ave.
Philadelphia, PA 19111
Phone: (215) 728-0417
Fax: (215) 728-3639
Email : ramona.swaby@fccc.edu

Rev. 12/07 4/08 ***STUDY CHAIR LIASON (SCL)***

LaTrice Vaughn, R.N.
Breast Care and Research Center
Indiana University
Simon Cancer Center
535 Barnhill Dr., RT-473
Indianapolis, IN 46202-5289
Phone: (317) 278-3730
Fax: (317) 278-6796
Rev. 5/08 Pager: (317) 312-1822
Email : lgvaughn@iupui.edu
Page (317) 312-1822 for “patient in office” questions only.

NCCTG CO-CHAIR

Donald W. Northfelt, M.D.
Mayo Clinic
13400 East Shea Blvd.
Scottsdale, AZ 85259
Phone: (480) 301-8000
Fax: (480) 301-4359
Email: northfelt.donald@mayo.edu

CALGB CO-CHAIR

Chau T. Dang, M.D.
Memorial Sloan-Kettering Cancer Center
1275 York Ave.
New York, NY 10021
Phone: (212) 639-7940
Fax: (212) 717-3619
Email: dangc@mskcc.org

This study is supported by the NCI Cancer Trials Support Unit (CTSU).

Institutions not aligned with ECOG-ACRIN will participate through the CTSU mechanism as outlined below and detailed in the CTSU logistical appendix.

- The study protocol and all related forms and documents must be downloaded from the protocol-specific Web page of the CTSU Member Web site located at https://members.ctsu.org
- Send completed site registration documents to the CTSU Regulatory Office. Refer to the CTSU logistical appendix for specific instructions and documents to be submitted.
- Patient enrollments will be conducted by the CTSU. Refer to the CTSU logistical appendix for specific instructions and forms to be submitted.
- Data management will be performed by the ECOG-ACRIN. Case report forms (with the exception of patient enrollment forms) and clinical reports must be sent to ECOG-ACRIN unless otherwise directed by the protocol. Do not send study data or case report forms to the CTSU Data Operations.
- Data clarification forms and delinquency reports will be sent directly to the enrolling site by ECOG-ACRIN. Please send data clarification form responses and delinquent data to ECOG-ACRIN and do not copy CTSU Data Operations. Please mail data clarification form responses and delinquent data directly to ECOG-ACRIN unless otherwise directed. Each site should have a designated CTSU Administrator and Data Administrator and must keep their CTEP AMS account contact information current. This will ensure timely communication between the clinical site and the ECOG-ACRIN data center.

CANCER TRIALS SUPPORT UNIT (CTSU) ADDRESS AND CONTACT INFORMATION

<table>
<tr><th>To submit site registration documents:</th><th>For patient enrollments:</th><th>Submit study data directly to the Lead Cooperative Group unless otherwise specified in the protocol:</th></tr>
<tr><td>CTSU Regulatory Office
1818 Market Street,
Suite 1100
Philadelphia, PA 19103
Phone - 1-888-823-5923
Fax – 215-569-0206
Email:
CTSURegulatory@ctsu.coccg.org (for submitting regulatory documents only)</td><td>CTSU Patient Registration
Voice Mail – 1-888-462-3009
Fax – 1-888-691-8039
Hours: 9:00 AM – 5:30 PM Eastern Time, Monday – Friday (excluding holidays)

[Registrations received after 5:00 PM ET will be handled the next business day. For CTSU patient enrollments that must be completed within approximately one hour, or other extenuating circumstances, call 301-704-2376 between 9:00 AM and 5:30 PM ET.</td><td>ECOG-ACRIN Operations Office - Boston,
FSTRF, 900 Commonwealth Avenue, Boston, MA 02215 (ATTN: DATA).
Phone # 617-632-3610
Fax # 617-632-2990
Data should be sent via postal mail.
Do not submit study data or forms to CTSU Data Operations. Do not copy the CTSU on data submissions.</td></tr>
<tr><td colspan="3">The most current version of the study protocol and all supporting documents must be downloaded from the protocol-specific Web page of the CTSU Member Web site located at https://www.ctsu.org. Access to the CTSU members' websiteis managed through the Cancer Therapy and Evaluation Program - Identity and Access Management (CTEP-IAM) registration system and requires user log on with CTEP-IAM username and password. Permission to view and download this protocol and its supporting documents is restricted and is based on person and site roster assignment housed in the CTSU RSS.</td></tr>
<tr><td colspan="3"><u>For patient eligibility or treatment-related questions</u>: Contact the Study PI of the Coordinating Group.</td></tr>
<tr><td colspan="3"><u>For questions unrelated to patient eligibility, treatment, or data submission</u> contact the CTSU Help Desk by phone or e-mail:
CTSU General Information Line – 1-888-823-5923, or ctsucontact@westat.com. All calls and correspondence will be triaged to the appropriate CTSU representative.</td></tr>
<tr><td colspan="3"><u>For detailed information on the regulatory and monitoring procedures for CTSU sites,</u> please review the CTSU Regulatory and Monitoring Procedures policy located on the CTSU members' website https://www.ctsu.org> education and resources tab > CTSU Operations Information >CTSU Regulatory and Monitoring Policy</td></tr>
<tr><td colspan="3">The CTSU Public Web site is located at: https://www.ctsu.org
The CTSU Registered Member Web site is located at https://members.ctsu.org</td></tr>
</table>

Rev. 1/15

Rev. 1/15

CTSU logistical information is located in Appendix VIII.

Schema – classical (every 3 week) AC

Choice of classical versus dose dense AC scheduling based on investigator's discretion

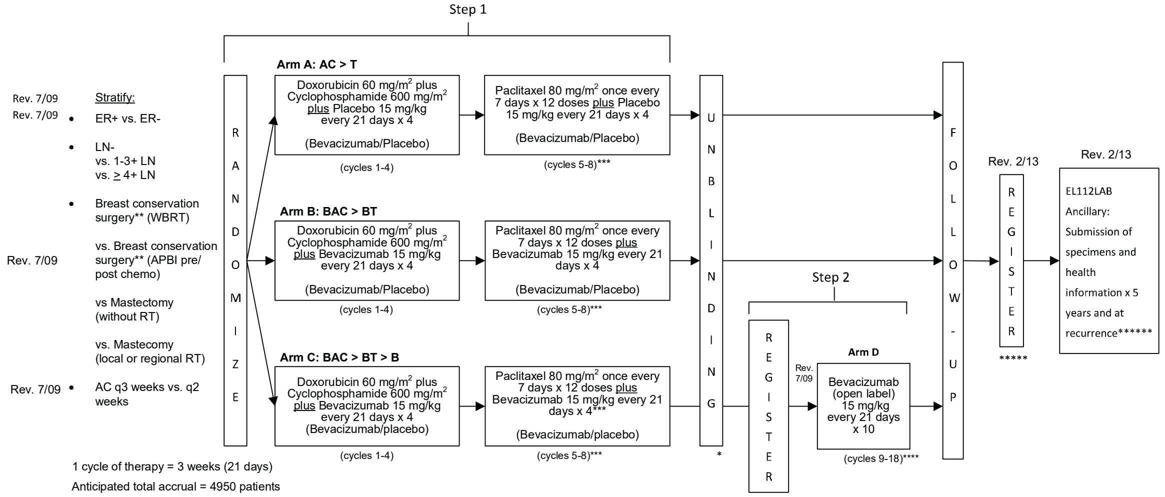

NOTE: All patients and treating physicians will be blinded as to assigned treatment group through Cycle 7. All patients and physicians will be unblinded on Day 1 of Cycle 8.

All patients with ER+ and/or PR+ breast cancer should receive hormonal therapy. Hormonal therapy should begin within 6 weeks of completing chemotherapy. For patients in Arm D, hormonal therapy will be administered concurrently with bevacizumab. Please see section 5.1.3 for additional details regarding hormonal therapy.

Post-mastectomy RT is required for all patients with a primary tumor ≥ 5 cm or involvement of 4 or more lymph nodes. Post-mastectomy RT may be administered at the investigator's discretion for all other mastectomy patients. For patients planned to receive radiation, treatment should begin within 6 weeks of completing chemotherapy. For patients in Arm D, radiation will be given concurrently with bevacizumab. Please see section 5.1.4 for additional details regarding radiation therapy.

Rev. 5/08 RT is required for patients who underwent breast conservation surgery**. WBRT is preferred but APBI is allowed. Patients interested in APBI should be encouraged to enroll in NSABP B-39. WBRT should begin within 6 weeks of completing chemotherapy. WBRT may not have been administered prior to protocol entry. APBI may be administered prior to protocol entry OR at the completion of protocol chemotherapy. If APBI was completed prior to study entry, D1 of protocol therapy must be at least 4 weeks after the completion of APBI. If APBI is planned post-chemotherapy, radiation should begin within 6 weeks of completing chemotherapy. For Arm C patients registered to Step 2, Arm D, radiation will be given concurrently with bevacizumab. Please see section 5.1.4 for additional details regarding radiation therapy

Abbreviations: WBRT - whole breast radiation therapy, APBI - accelerated partial breast radiation

* All patients will be unblinded on Day 1 of Cycle 8. However, all patients will continue to receive Cycle 8 treatment as outlined. See Section 4.6.

** Breast conservation surgery includes lumpectomy, partial mastectomy, and excisional biopsy.

Rev. 7/09 *** Cycle 5 treatment to begin 3 weeks after last AC treatment.

Rev. 7/09 **** Step 2 Arm D treatment must begin no later than 2 months following the last dose of Step 1 bevacizumab.

Rev. 10/09, 7/10, 9/10 **NOTE:** QOL assessment should be completed on the first 500 patients entered onto E5103 who receive protocol treatment at: baseline (prior to starting treatment) and at 6 weeks, 18 or 22 weeks, 12 months and 18 months post-registration.

NOTE: The accrual goal for the QOL assessment has been met and this component has closed.

Rev. 2/13 ***** Patients become potentially eligible for EL112LAB if disease free and were registered to step 1 (Arm A,B or C) at least 4.5 years (54 months) and no more than 7.5 years (90 months) prior to registration to step 3. To assist sites, ECOG will provide a list of patients with date ranges of their potential eligibility. Requirements for EL112LAB do NOT impact or change the requirements of the E5103 protocol.

Rev. 2/13 ****** Following registration to EL112LAB: blood samples are submitted at registration, yearly for 5 years and upon recurrence; primary tumor (if not previously submitted) after registration; recurrence tumor tissue upon recurrence; and health information at registration and yearly for 5 years.

Schema – dose dense (every 2 week) AC

Choice of classical versus dose dense AC scheduling based on investigator's discretion

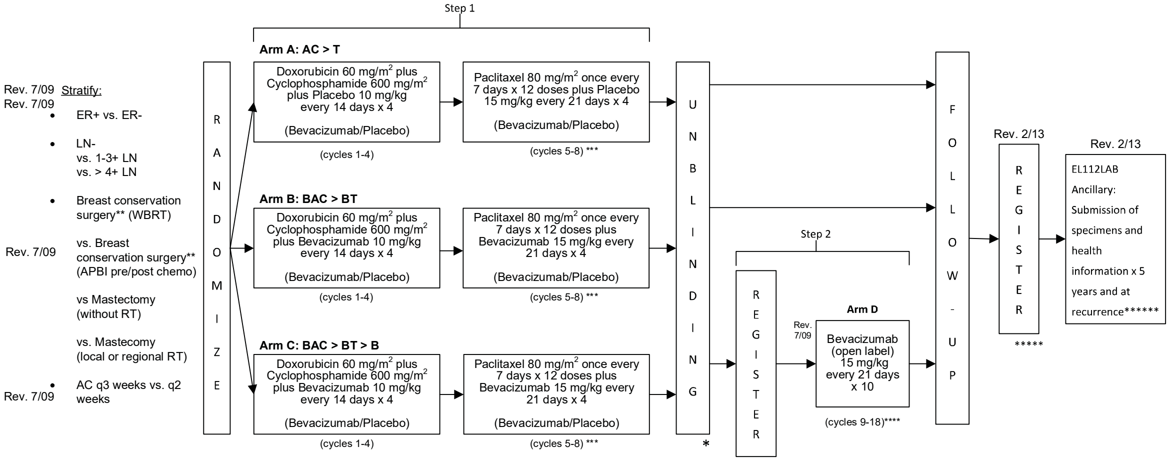

For cycles 1-4: 1 cycle of therapy = 2 weeks (14 days) **NOTE:** Filgrastim (5 μg/kg SQ) days 2-11 or Pegfilgramstim (6 mg SQ) day 2 required for cycles 1-4 ONLY
For cycle 5 and beyond: 1 cycle of therapy = 3 weeks (21 days)

Anticipated total accrual = 4950 patients

NOTE: All patients and treating physicians will be blinded as to assigned treatment group through Cycle 7. All patients and physicians will be unblinded on Day 1 of Cycle 8.

Rev. 7/09 All patients with ER+ and/or PR+ breast cancer should receive hormonal therapy. Hormonal therapy should begin within 6 weeks of completing chemotherapy. For patients in Arm D, hormonal therapy will be administered concurrently with bevacizumab. Please see section 5.2.3 for additional details regarding hormonal therapy.

Rev. 7/09 Post-mastectomy RT is required for all patients with a primary tumor ≥ 5 cm or involvement of 4 or more lymph nodes. Post-mastectomy RT may be administered at the investigator's discretion for all other mastectomy patients. For patients planned to receive radiation, treatment should begin within 6 weeks of completing chemotherapy. For patients in Arm D, radiation will be given concurrently with bevacizumab. Please see section 5.2.4 for additional details regarding radiation therapy.

Rev. 7/09 RT is required for patients who underwent breast conservation surgery**. WBRT is preferred but APBI is allowed. Patients interested in APBI should be encouraged to enroll in NSABP B-39. WBRT should begin within 6 weeks of completing chemotherapy. WBRT may not have been administered prior to protocol entry. APBI may be administered prior to protocol entry OR at the completion of protocol chemotherapy. If APBI was completed prior to study entry, D1 of protocol therapy must be at least 4 weeks after the completion of APBI. If APBI is planned post-chemotherapy, radiation should begin within 6 weeks of completing chemotherapy. For Arm C patients registered to Step 2, Arm D, radiation will be given concurrently with bevacizumab. Please see section 5.2.4 for additional details regarding radiation therapy.

Abbreviations: WBRT - whole breast radiation therapy, APBI - accelerated partial breast radiation

* All patients will be unblinded on Day 1 of Cycle 8. However, all patients will continue to receive Cycle 8 treatment as outlined. See section 4.6.

** Breast conservation surgery includes lumpectomy, partial mastectomy, and excisional biopsy.

Rev. 7/09 *** Cycle 5 treatment to begin 2 weeks after last AC treatment.

Rev. 7/09 **** Step 2 Arm D treatment must begin no later than 2 months following the last dose of Step 1 bevacizumab.

Rev. 10/09, 7/10, 9/10 **NOTE:** QOL assessment should be completed on the first 500 patients entered onto E5103 who receive protocol treatment at: baseline (prior to starting treatment) and at 6 weeks, 18 or 22 weeks, 12 months and 18 months post-registration.

NOTE: The accrual goal for the QOL assessment has been met and this component has closed.

Rev. 2/13 ***** Patients become potentially eligible for EL112LAB if disease free and were registered to step 1 (Arm A,B or C) at least 4.5 years (54 months) and no more than 7.5 years (90 months) prior to registration to step 3. To assist sites, ECOG will provide a list of patients with date ranges of their potential eligibility. Requirements for EL112LAB do NOT impact or change the requirements of the E5103 protocol.

Rev. 2/13 ****** Following registration to EL112LAB: blood samples are submitted at registration, yearly for 5 years and upon recurrence; primary tumor (if not previously submitted) after registration; recurrence tumor tissue upon recurrence; and health information at registration and yearly for 5 years.

1. Introduction

Breast cancer remains a devastating disease affecting the lives of 211,000 women in the United States in 2003 (1). Improved early detection and advances in systemic therapy of early stage disease have lead to a small decline in overall breast cancer mortality since 1989 (2). Advances in adjuvant therapy have generally been incremental. In the 1970s, cyclophosphamide, methotrexate, fluorouracil (CMF) was the gold standard. In the 1980s, trials first suggested that the anthracyclines were marginally superior (3,4). By the 1990s, anthracyclines had become the mainstay of adjuvant chemotherapy; clinical trials first suggested benefit from early use of taxanes (5,6). Most recently administering doxorubicin and cyclophosphamide followed by paclitaxel (AC>T) in a dose dense schedule improved both disease free and overall survival in patients with ER- disease; no improvement was found in patients with ER+ disease (7,8). Paclitaxel schedule appears to be particularly important; weekly paclitaxel improved overall response rate and progression free survival in patients with metastatic disease compared to every three weekly paclitaxel (9). Data from the completed N9831 and E1199 trials confirm the safety and feasibility of administering paclitaxel weekly for 12 weeks in the adjuvant setting (10,11).

Further advances will require new therapeutic strategies that are firmly rooted in an understanding of basic breast cancer biology. Over the last two decades substantial laboratory and indirect clinical evidence has accumulated to support the central role of angiogenesis in breast cancer progression (reviewed by Gasparini (12)). This nascent vascular network provides a novel opportunity for therapy. Indeed the combination of paclitaxel and bevacizumab, an antiangiogenic agent, improved response rate, time to progression compared to paclitaxel alone in patients with newly diagnosed metastatic breast cancer (Miller et al, ASCO 2005). However, as tumors progress increasing numbers of pro-angiogenic peptides are produced (13). As such, the most successful clinical application of angiogenesis inhibitors is likely to be in patients with micrometastatic disease that is in the adjuvant setting. This trial is designed to test that hypothesis.

1.1 Angiogenesis in Breast Cancer

Extensive laboratory data suggests that angiogenesis plays an essential role in breast cancer development, invasion and metastasis. Hyperplastic murine breast papillomas (14) and histologically normal lobules adjacent to cancerous breast tissue (15) support angiogenesis in preclinical models suggesting that angiogenesis precedes transformation of mammary hyperplasia to malignancy. Transfection of tumor cells with angiogenic stimulatory peptides such as fibroblast growth factor-1 or –4 (16,17), vascular endothelial growth factor (VEGF) (18,19), or progelatinase-B (matrix metalloproteinase-9, MMP-9) (20), increases tumor growth, invasiveness, microvasculature and metastasis (21). Conversely, transfection of tumor cells with inhibitors of angiogenesis, including thrombospondin-1 (22) or tissue inhibitor of metalloproteinase-4 (TIMP-4) (23) decreases growth and metastasis (24).

Clinicopathologic correlations also confirm the central role of angiogenesis in breast cancer progression. Fibrocystic lesions with the highest vascular density were associated with a greater risk of breast cancer (25). Two distinct vascular patterns have been described in association with ductal carcinoma *in situ*: a diffuse increase in stromal vascularity between duct lesions and a dense rim of

microvessels adjacent to the basement membrane of individual ducts (26,27). Microvessel density (MVD) was highest with histopathologically aggressive DCIS lesions (26) and was associated with increased VEGF expression (28).

Weidner and colleagues found a statistically significant correlation between MVD and the incidence of metastases, relapse-free and overall survival in patients with early breast cancer (29,30). Investigation in this area has flourished with other groups modifying the technique to use different endothelial antibodies (31,32) and counting strategies (33). These studies have generally (34,35), though not uniformly (36), validated the poor prognosis and early relapse associated with increasing MVD. The extent of primary tumor vascularization has also been associated with tumor shedding at the time of surgery (37) and the probability of bone marrow micrometastases (38).

1.2 Vascular Endothelial Growth Factor

Angiogenesis requires stimulation of vascular endothelial cells through the release of angiogenic peptides, of which the vascular endothelial growth factor (VEGF) is the most potent. VEGF is a highly conserved, homodimeric, secreted, heparin-binding glycoprotein whose dominant isoform has a molecular weight of ~45,000 (39). VEGF produces a number of biologic effects, including endothelial cell mitogenesis and migration, induction of proteinases leading to remodeling of the extracellular matrix, increased vascular permeability and vasodilation, immune modulation via inhibition of antigen-presenting dendritic cells and maintenance of survival for newly formed blood vessels by inhibiting endothelial cell apoptosis. The biologic effects of VEGF are mediated through binding to one of three endothelial surface receptors VEGF-R1 (flt-1), VEGF-R2 (flk-1/kdr), VEGF-R3; binding to the co-receptor neurophilin enhances signaling.

Though multiple angiogenic factors are commonly expressed by invasive human breast cancers, the 121-amino acid isoform of VEGF predominates (13). Several studies have found an inverse correlation between VEGF expression and overall survival in both node-positive and node-negative patients (40-43). Eppenberger confirmed the negative prognostic value of VEGF expression for both relapse and survival (44).

1.3 Bevacizumab

An antibody directed against VEGF (rhuMAb VEGF; bevacizumab) inhibited the growth of several human tumors in animal models (45,46). A phase I trial confirmed the safety of bevacizumab and found the expected decrease in circulating VEGF levels (47). Bevacizumab has been studied in at least 3500 patients in a number of Phase I, II, and III clinical trials in a number of tumor types, including colorectal, breast, lung, and renal carcinoma.

In the Phase I and II clinical trials, four potential bevacizumab-associated safety signals were identified: hypertension, proteinuria, thromboembolic events, and hemorrhage. Completed Phase II and Phase III studies of bevacizumab have further defined the safety profile of this agent in patients with metastatic malignancies. Also during the Phase III trials, three new possible bevacizumab-associated safety signals were identified: congestive heart failure (CHF) in patients who had been exposed to anthracyclines, gastrointestinal perforations, and wound healing complications.

1.3.1 Hypertension

Hypertension has been seen in all clinical trials to date and oral medications have been used to manage the hypertension when indicated. Grade 4 hypertensive events are rare.

1.3.2 Proteinuria

Proteinuria, ranging from asymptomatic and transient events detected on routine dipstick urinalysis to nephrotic syndrome, has been seen in all clinical trials to date. The majority of proteinuria events have been grade 1 or 2. In the phase III pivotal trial in metastatic colorectal cancer (CRC), the rate of grade 3 or greater proteinuria was < 1% in both treatment arms.

1.3.3 Thromboembolic Events

Both venous and arterial thromboembolic (TE) events, ranging in severity from catheter-associated phlebitis to fatal, have been reported in patients treated with bevacizumab in the colorectal cancer trials and, to a lesser extent, in patients treated with bevacizumab in non-small cell lung cancer (NSCLC) and breast cancer trials. In the phase III pivotal trial in metastatic CRC, there was a slightly higher rate of venous TE events that was not statistically significant in the patients on the bevacizumab treatment arm (16% versus 19%). There was also a higher rate of arterial TE events (1% versus 3%) such as myocardial infarction, transient ischemia attack, cerebrovascular accident/stroke and angina/unstable angina. A pooled analysis of the rate of arterial TE events from 5 randomized studies showed that treatment with bevacizumab increased the risk two to three fold (up to 5%) of having an arterial TE event. Furthermore, certain baseline characteristics conferred additional risk; specifically age > 65 years and prior arterial TE event.

1.3.4 Hemorrhage

Bleeding events have been observed in all clinical trials. The majority of the events are grade 1 and grade 2 mucocutaneous; typically epistaxis. Major hemorrhage has been observed. In a Phase II NSCLC trial, 6 of 66 bevacizumab-treated patients experienced life-threatening hemoptysis or hematemesis. Four of these events were fatal. Centrally located lesions that were necrotic or cavitary and squamous cell histology were identified as possible risk factors for bleeding.

1.3.5 Gastrointestinal Perforation

In the Phase III colorectal trial, gastrointestinal perforation and wound dehiscence, complicated by intra-abdominal abscesses, occurred at an increased incidence in patients receiving bevacizumab compared with control subjects. These events varied in type and severity, ranging from free air seen on kidney, ureter, and bladder x-ray (KUB), which resolved without treatment, to a colonic perforation with abdominal abscess, which was fatal. The overall rates of GI perforation are low (<5%) and to date, there have been no

characteristics identified that can predict risk. GI perforation has been observed in other, nonCRC tumor types as well (see Investigator Brochure).

1.3.6 Congestive Heart Failure

The association between bevacizumab treatment and cardiac dysfunction remains uncertain. Congestive heart failure was reported by 2 (2.7%) of 75 patients enrolled in the phase II study of bevacizumab monotherapy. Both patients had had prior anthracycline treatment and left chest wall radiation; one had concurrent pericardial involvement with metastatic disease (48,49). In a phase III trial of capecitabine with or without bevacizumab, two (0.9%) patients treated with capecitabine alone compared to 7 (3.1%) patients receiving capecitabine plus bevacizumab developed clinically significant congestive heart failure. Of note, all patients in this trial had had prior anthracycline treatment (50).

A recently published phase II study in patients with refractory acute myelogenous leukemia (AML), reported 5 cases of cardiac dysfunction (congestive heart failure or decreases of LVEF to < 40%) out of 48 patients treated with sequential 1-beta-darabinofuranosylcytosine, mitoxantrone, and bevacizumab (51). All but one of these patients had significant prior exposure to anthracyclines as well. Two other studies investigated concurrent administration of anthracyclines and bevacizumab. Wedam treated 21 patients with inflammatory breast cancer with neoadjuvant docetaxel, doxorubicin and bevacizumab. Though no patient developed clinically apparent CHF, two patients had asymptomatic decreases in LVEF to below 40% (52). In another trial, patients with soft tissue sarcoma were treated with high dose doxorubicin (75 mg/m^2; total dose 600 mg/m^2) and bevacizumab. Two of 17 patients treated with bevacizumab and doxorubicin (75 mg/m^2) developed CHF (one Grade 3, one Grade 4); an additional 4 patients had asymptomatic decreases in LVEF (53). There are no data to suggest that bevacizumab increases the risk of CHF in patients without exposure to anthracyclines. A pilot safety study, E2104, will further define the impact of incorporating bevacizumab into an anthracycline containing adjuvant therapy regimen.Neutropenia

1.3.7 Neutropenia

When combined with chemotherapy, bevacizumab increased the risk of neutropenia compared to chemotherapy alone. In a phase 3 trial with Irinotecan-Fluorouracil-Leucovorin (IFL) +/- bevacizumab in colorectal cancer, grade 3-4 neutropenia was 21% in the bevacizumab + IFL arm vs. 14% in the IFL arm (grade 4 neutropenia was 3% vs. 2%). In a phase 3 trial with carboplatin and paclitaxel +/- bevacizumab is NSCLC, the bevacizumab-containing arm was associated with an increased rate of grade 4 neutropenia (27% vs. 17%), febrile neutropenia (5.4% vs. 1.8%), and an increased rate of infection with neutropenia (4.4% vs. 2.0%) with three fatal cases in the

bevacizumab + chemotherapy arm vs. none in the chemotherapy control arm.

1.3.8 Other Safety Concerns

Other safety concerns seen to date—asthenia, pain, headache, fever, chills, rash, infection, epistaxis, and mouth ulceration—are not thought to be clinically significant in that they rarely or never required treatment or study drug discontinuation. Please review the bevacizumab Investigator Brochure for additional details regarding safety experience with bevacizumab.

Reversible Posterior Leukoencephalopathy Syndrome (RPLS) or similar leukoencephalopathy syndrome: RPLS or clinical syndromes related to vasogenic edema of the white matter, have been rarely reported in association with bevacizumab therapy (<1%). Clinical presentations are variable and may include altered mental status, seizure and cortical visual deficit. Hypertension (HTN) is a common risk factor and was present in most (though not all) patients on bevacizumab who developed RPLS. MRI scans are key to diagnosis and typically demonstrate vasogenic edema (hyperintensity in T2 and FLAIR images and hypointensity in T1 images) predominantly in the white matter of the posterior parietal and occipital lobes; less frequently, the anterior distributions and the gray matter may also be involved. RPLS should be in the differential diagnosis in patients presenting with unexplained mental status change, visual disturbance, seizure or other CNS findings. RPLS is potentially reversible, but timely correction of the underlying causes, including control of BP and interruption of the offending drug, is important in order to prevent progression to irreversible tissue damage.

1.4 Bevacizumab in Breast Cancer

A dual-institution phase II study of bevacizumab in patients with previously treated metastatic breast cancer enrolled 75 patients in three successive dose cohorts, 3 mg/kg (n=18), 10 mg/kg (n=41) and 20 mg/kg (n=16) every other week. Overall 17% of patients were responding or stable at 22 weeks; four patients continued therapy without progression for over 12 months (49). Burstein and colleagues combined bevacizumab with vinorelbine in a phase II study of 56 patients with advanced breast cancer; most patients had had prior chemotherapy for metastatic disease. Overall response rate was 30%; no unexpected toxicities were reported (54).

Given the efficacy and minimal toxicity, a phase III study was initiated in 462 patients with anthracycline- and taxane- refractory disease. Patients were randomized to receive capecitabine with or without bevacizumab; primary endpoint was progression-free survival as assessed by an independent review facility (IRF). Therapy was generally well tolerated with adverse events leading to discontinuation in 12% of patients in each group. As expected, bevacizumab therapy induced hypertension, proteinuria and minor mucosal bleeding but these toxicities were rarely severe (Table1). Combination therapy significantly increased the response rates whether designated by the IRF (9.1% vs. 19.8%; p=0.001) or the local investigators (19.1% vs. 30.2%; p=0.006). As many of the excess responses in the combination group were relatively short-lived,

progression free survival was similar in both groups (4.17 vs. 4.86 mos.; hazard ratio = 0.98) (50).

Table 1. Bevacizumab-associated toxicities

	Cap (n=215)	Cap + Bev (n=229)
Hypertension (grade 3 only)	0.5%	17.9%
Thromboembolic	5.6%	7.4%
PE	1.4%	1.3%
DVT	2.3%	6.1%
Bleeding	11.2%	28.8%
Grade ≥ 3 or serious	1.4%	0.4%
Proteinuria	7.4%	22.3%
Grade ≥ 3	0	0.9%

Cap=capecitabine, PE=pulmonary embolism, DVT=deep venous thrombosis

Though clearly a negative trial, this initial phase III randomized trial represented an important proof of concept. The activity of bevacizumab first suggested in phase II trials was confirmed; toxicity was minimal and rarely limited therapy.

That potential has recently been realized in E2100, a randomized trial of weekly paclitaxel with or without bevacizumab as initial chemotherapy in 722 patients with metastatic breast cancer. The addition of bevacizumab to weekly paclitaxel (90 mg/m^2 weekly x 3 out of 4 weeks) increased response rate (20.9% vs. 35.8%; *p*<0.0001) and prolonged progression-free survival by over 5 months (*p*<0.0001). Toxicity in E2100 was similarly reassuring.

	Pac (n=330)		Pac + Bev (n=342)	
	G3	G4	G3	G4
Hypertension	2%	0	15%	<1%
Thromboembolic	2%	2%	2%	0
Bleeding	0	0	2%	<1%
Proteinuria	0	0	1%	1%
Neuropathy	17%	1%	22%	1%
Fatigue	4%	<1%	8%	<1%
Neutropenia	NR	3%	NR	3%
Decreased LVEF	0	0	<1%	0

Pac – paclitaxel, Bev – bevacizumab, LVEF – left ventricular function

Angiogenesis inhibitors significantly curtail primary tumor growth and establishment of metastases in several pre-clinical minimal disease models; overt shrinkage of large, well established tumors is less common. As tumors progress, increasing numbers of pro-angiogenic peptides are produced making resistance to any single anti-angiogenic agent more likely (13). As such, the most successful clinical application of angiogenesis inhibitors is likely to be in patients with micrometastatic disease that is in the adjuvant setting. E5103 will directly test that hypothesis.

1.5 Selection of Adjuvant Chemotherapy

E5103 uses a sequential anthracycline (AC) and taxane (paclitaxel) chemotherapy backbone The AC may be administered using the classical every three week or dose dense every two week schedule. The choice of AC schedule

will be left to the treating investigator's (and patient's) discretion. Patients will be stratified for AC schedule prior to randomization. As preclinical data and the results of E2100 support the use of a weekly paclitaxel regimen, all patients will receive weekly paclitaxel for 12 weeks following AC.

1.6 Gender and Ethnicities Statement

This study is open to patients of all ethnic backgrounds. Historically, approximately 15% of patients enrolled on ECOG breast cancer studies are members of minority ethnic groups. It is anticipated that a similar proportion of patients on this study will be members of ethnic minorities. Based on current data, ECOG-ACRIN believes that interactions between ethnicity and treatment are not expected. Accrual will not be increased to meet subgroup targets.

Rev. 10/09 1.7 Predictive Biomarkers for Bevacizumab

Much of the genetic variability seen in common polymorphic sites in genes that regulate tumor angiogenesis appear to be inherited. Thus, the therapeutic heterogeneity observed with an agent that targets a host directed process (i.e. bevacizumab) might best be explained by host-imprinted variability (e.g. single nucleotide polymorphisms). There are multiple single nucleotide polymorphisms (SNPs) within the drug target for bevacizumab, VEGF. Prior studies suggest that SNPs within VEGF can impact outcome in conditions regulated by angiogenesis including cancer risk and prognosis, retinopathy, nephropathy, pre-eclampsia, and vasculopathy. SNPs in VEGF have been shown to affect VEGF production which may explain its ability to predict outcome. More recently, retrospective analysis of E2100 demonstrated that SNPs in VEGF can predict which subgroup of patients with metastatic breast cancer receiving bevacizumab will experience a prolonged median overall survival and which subgroup will be protected from hypertension.

Although a candidate gene approach is a high yield strategy for biomarker discovery, the variability seen in most disease processes is highly dependent on complex gene-gene interactions. Recently, advances in technical platforms and bioinformatics have made genome wide association studies (GWAS) feasible. This comprehensive approach has led to some important discoveries including identification of genes, SNPs, and haplotypes associated with breast cancer, lung cancer, colon cancer, prostate cancer, myocardial infarction, and type-2 diabetes. Thus, GWAS has become a powerful platform for biomarker discovery.

Rev. 10/09 1.8 Pharmacogenetics/Pharmacogenomics Data Analysis

When pharmacogenetics/pharmacogenomics data are available, they will be used first for population stratification assessment. Then they will be correlated with efficacy and toxicity outcomes. The goal of the data analysis is to identify SNP/treatment interactions, i.e. SNPs that predict bevacizumab's efficacy and toxicity. The data analysis will be accomplished through Cox regression model for efficacy variables and the generalized linear model for toxicity outcome variables. Additional gene ontology analyses will be performed to evaluate the functions of significant genes.

Analysis for the association of toxicity with genotype will occur as these results are reported. Genetic effect on efficacy will first be analyzed with the reporting of the first interim analysis as well as with subsequent updates (i.e. other interim analyses and/or final analysis).

2. Objectives

2.1 Primary Objective

To determine the disease-free survival of patients (defined as invasive disease-free survival (IFDS) per Section 6.3) with lymph node positive and high risk lymph node negative breast cancer randomized to treatment with either doxorubicin/cyclophosphamide plus placebo followed by paclitaxel (AC + Placebo > T + Placebo) or the same chemotherapy regimen plus bevacizumab.

2.2 Secondary Objectives

2.2.1 To compare short-term (20-24 weeks) versus long-term (50-54 weeks) bevacizumab therapy

2.2.2 To compare overall survival

2.2.3 To evaluate toxicity

Rev. 10/09

2.2.4 To evaluate the association between outcomes in E5103 (disease-free survival, overall survival and toxicities) and genotype (derived from candidate single nucleotide polymorphisms and genome wide evaluations).

Rev. 10/09

2.3 Quality of Life Objectives

2.3.1 To compare the quality of life of breast cancer patients treated with AC/Paclitaxel and bevacizumab or placebo, in terms of physical symptoms, physical functioning, psychological state and social functioning over an 18 month period.

2.3.2 To determine the impact of theoretical biomarker information on patients' willingness to accept the toxicities of bevacizumab for the estimated potential benefit.

Rev. 2/13

2.4 Determinants of Late Relapse Ancillary Study (EL112LAB)

2.4.1 To create a biospecimen repository including plasma, serum and CellSearch™ cassettes containing circulating tumor cells (CTC) for evaluating determinants of late relapse, including candidate biomarkers reflecting occult tumor burden (e.g., CTCs and plasma tumor DNA) and host factors (e.g., estrogen, insulin-IGF axis, inflammation, etc).

2.4.2 To create a biorepository of metastatic tumor samples in patients who have had a late relapse.

2.4.3 To determine body mass index (BMI) and comorbidity burden in patients with operable breast cancer five or more years after diagnosis.

2.4.4 To determine whether there is a relationship between late relapse and BMI at diagnosis and at 5 years after diagnosis, and whether BMI-associated inflammatory and/or metabolic biomarkers are associated with early and late recurrence.

3. Selection of Patients

Each of the criteria in the checklist that follows must be met in order for a patient to be considered eligible for this study. Use the checklist to confirm a patient's eligibility. The checklist must be completed for each patient and must be signed and dated by the treating physician. **Please submit the completed eligibility checklist as outlined in the Forms Submission Schedule, which is posted on the ECOG website with the protocol (www.ecog.org).** A copy of the completed checklist should also be maintained in the patient's chart.

In calculating days of tests and measurements, the day a test or measurement is done is considered Day 0. Therefore, if a test is done on a Monday, the Monday four weeks later would be considered Day 28.

ECOG-ACRIN Patient No. ____________________________

Patient's Initials (L, F, M) _______________________

Physician Signature and Date ___________________

NOTE: All questions regarding eligibility should be directed to the study chair or study chair liaison.

NOTE: Institutions may use the eligibility checklist as source documentation if it has been reviewed, signed, and dated prior to registration/randomization by the treating physician.

3.1 STEP 1: Registration to Arm A, B, C

_____ 3.1.1 Patients must have histologically confirmed adenocarcinoma of the breast at significant risk of distant recurrence based on at least one of the following criteria:

Rev. 7/09 **For Axillary Lymph Node Positive Disease:**

Rev. 7/09 _____ 3.1.1.1 Involvement of at least one sentinel or axillary lymph node on routine histologic examination. Patients with negative
Rev. 7/09 sentinel nodes and negative axillary nodes or involvement only demonstrated by immunohistochemistry are not eligible unless they meet one of the other eligibility criteria below.

NOTE: Consider intramammary nodes as equivalent to axillary nodes for the purposes of eligibility and stratification.

Rev. 7/09 **For Axillary Lymph Node Negative Disease:**

_____ 3.1.1.2 ER negative tumor ≥1 cm

_____ 3.1.1.3 ER+ tumor ≥ 5 cm regardless of recurrence score

_____ 3.1.1.4 ER+ tumor ≥1 cm but < 5 cm with a recurrence score ≥ 11. (Patients enrolled in the TAILORx trial are eligible.)

Date of histological confirmation of adenocarcinoma of the breast at significant risk of distant recurrence__________

Rev. 7/09

NOTE: Axillary dissection is strongly encouraged in patients with lymph node involvement identified on sentinel node biopsy.

NOTE: Premenopausal patients with ER+ tumor may participate in the IBCSG SOFT trial.

NOTE: Premenopausal patients with ER- tumor may participate in S0230.

_____ 3.1.2 Patients must have completed definitive breast surgery including total mastectomy and axillary dissection (modified radical mastectomy), total mastectomy and sentinel node biopsy, breast conservation surgery and axillary dissection or breast conservation surgery and sentinel node biopsy.

Rev. 7/09

NOTE: Breast conservation surgery includes lumpectomy, partial mastectomy, and excisional biopsy.

_____ 3.1.3 Margins of breast conservation surgery or mastectomy must be histologically free of invasive breast cancer and ductal carcinoma in situ (DCIS). Patients with resection margins positive for lobular carcinoma in situ (LCIS) are eligible.

Rev. 7/09

_____ 3.1.4 Time from last surgery for breast cancer (breast conservation surgery, mastectomy, sentinel node biopsy, axillary dissection or re-excision of breast conservation surgery margins) to planned treatment start date must be > 28 days and ≤ 84 days.

Date of last surgery: __________

Scheduled Day 1 of protocol treatment: _________

Rev. 7/09

NOTE: Treatment start date more than 84 days or ≤ 28 days from date of last surgery is considered a protocol violation.

_____ 3.1.5 ECOG performance status of 0-1

_____ 3.1.6 Patients must have adequate organ function within ≤ 8 weeks prior to randomization, as measured by:

______ 3.1.6.1 Absolute neutrophil count ≥ 1000/mm^3

Absolute neutrophil count:________

Date of Test: ____________

______ 3.1.6.2 Platelet count ≥ 100,000/mm^3

Platelet count:____________________

Date of Test: ____________

______ 3.1.6.3 Total bilirubin ≤ 1.5 mg/dL

Total bilirubin:____________________

Date of Test: ____________

Rev. 7/09 ______ 3.1.6.4 AST ≤ 2 X upper limit of normal

AST:________________

Date of Test: ____________

Upper Limit of Normal: ______________

______3.1.6.5 Serum creatinine ≤ 1.5 mg/dL

Serum Creatinine:___________

Date of Test: ____________

Rev. 7/10 ______ 3.1.6.6 Urine protein: creatinine (UPC) ratio <1.0* or 24-hour protein

Urine protein: creatinine ratio:________ or 24-hour protein: ________

Date of Test: ____________

Rev. 7/09 ______ 3.1.6.7 PTT ≤ 1.5 X ULN

PTT: _______________

Date of Test: ____________

Normal: _______________

_____ 3.1.6.8 LVEF ≥ institutional limits of normal by MUGA or ECHO

LVEF: ______________

Date of Test: ____________

Institutional limit of normal: _____________

*Please see Appendix V for instructions on how to obtain the urine protein:creatinine ratio

_____ 3.1.7 Patients who have undergone breast conservation surgery must receive radiation. Prior to randomization, the investigator must specify the planned radiation technique.

_____ Whole breast radiation (WBRT) after chemotherapy

_____ Accelerated partial breast radiation (APBI) after chemotherapy

_____ Accelerated partial breast radiation (APBI) prior to chemotherapy

NOTE: If APBI was completed prior to study entry, day 1 of protocol therapy must be at least 4 weeks after the completion of APBI.

Last day of APBI __________

Planned day 1 of protocol therapy ________

Rev. 5/08 _____ 3.1.8 Post-mastectomy RT is required for all patients with a primary tumor of ≥ 5 cm or involvement of 4 or more lymph nodes. Post-mastectomy RT may be administered at the investigator's discretion for all other mastectomy patients.

Is post-mastectomy radiation planned? Yes/No_______

Rev. 7/09 _____ 3.1.9 Patients with HER2 + (3+ by IHC or FISH ratio ≥ 2) breast cancer are not eligible

_____ 3.1.10 Patients with synchronous bilateral breast cancer (diagnosed within one month) are eligible if the higher TNM stage tumor meets the eligibility criteria for this trial.

_____ 3.1.11 Patients must not have clinical evidence of inflammatory disease or fixed axillary nodes at diagnosis.

_____ 3.1.12 Patients must not have received prior cytotoxic chemotherapy or hormonal therapy for this breast cancer. Prior treatment with an anthracycline, anthracenedione or taxane for any condition is not allowed.

NOTE: Prior use of tamoxifen for chemoprevention is allowed but must be discontinued at study entry. Similarly, prior raloxifene use is allowed but must be discontinued at study entry.

Rev. 7/09 _____ 3.1.13 Patients must not have had any major surgical procedure within 28 days of planned treatment start date.

Date of last major surgery __________

Scheduled day 1 of protocol treatment __________

NOTE: Non-operative biopsy or placement of a vascular access device is not considered a major surgery.

_____ 3.1.14 Patients may not have had placement of a vascular access device within 24 hours of planned Day 1 of treatment.

Is an indwelling vascular device planned? Yes/No_______

If yes, date of vascular device placement____________

Scheduled Day 1 of treatment________________

_____ 3.1.15 Patients must not have clinically significant cardiovascular or cerebrovascular disease, including:

<u>Any history of</u>

- Cerebrovascular disease including TIA, stroke or subarachnoid hemorrhage
- Ischemic bowel

<u>Within the last 12 months</u>

- Myocardial infarction
- Unstable angina
- Rev. 4/08 New York Heart Association (NYHA) class II or greater congestive heart failure
- Grade II or greater peripheral vascular disease

NOTE: See Appendix X for NYHA classification and peripheral vascular disease grading criteria

Active at study entry

- Uncontrolled hypertension defined as SBP > 160 or DBP > 90
- Uncontrolled or clinically significant arrhythmia.

Rev. 7/09

NOTE: Blood pressure must be obtained within ≤ 8 weeks prior to randomization.

NOTE: Patients with controlled atrial fibrillation are eligible.

_____ 3.1.16 Patients who require full dose anticoagulation may enroll provided they meet the following criteria:

- the patient must have an in-range INR (usually between 2 and 3) on a stable dose of warfarin or be on stable dose of LMW heparin.
- the patient must not have active bleeding or pathological conditions that carry high risk of bleeding (e.g. varices)

NOTE: Prophylactic use of anticoagulants to maintain patency of a vascular access device is permitted.

_____ 3.1.17 Patients must not have a bleeding diathesis, hereditary or acquired bleeding disorder or coagulopathy.

_____ 3.1.18 Patients must not have a non-healing wound or fracture. Patients with an abdominal fistula, gastrointestinal perforation, or intra-abdominal abscess within 6 months prior to randomization are not eligible.

_____ 3.1.19 Patients must not have hypersensitivity to paclitaxel or drugs using the vehicle Cremophor, Chinese hamster ovary cell products or other recombinant human antibodies.

_____ 3.1.20 Male or female patients age ≥ 18 years of age are eligible.

_____ 3.1.21 Women must not be pregnant or breast-feeding due to the potential harmful effects of bevacizumab on the developing fetus. All females of childbearing potential must have a blood or urine test within 7 days prior to randomization to rule out pregnancy.

Is patient a woman of child-bearing potential? _____ (Yes/No)

If yes, date of blood or urine test: ____________

_____ 3.1.22 Women of childbearing potential and sexually active males must use an accepted and effective method of contraception.

3.2 STEP 2: Unblinding and Re-registration to Arm D

_____ 3.2.1 Only Step I patients treated on Arm C who have not ended treatment per section 5.7 are eligible to register to Step 2, Arm D.

Rev. 2/13

3.3 Step 3: Registration to Ancillary Study EL112LAB

All patients who meet the following criteria are eligible to participate in the ancillary study:

_____ 3.3.1 Patient was registered to Step 1 (Arms A, B, or C) at least 4.5 years (54 months) and no more than 7.5 years (90 months) prior to registration to Step 3. It is preferable for patients to be registered to Step 3 at 5 years (60 months +/- 6 months) after registration to Step 1.

_____ 3.3.2 Patient is disease free, with no prior recurrence, at time of registration to Step 3.

_____ 3.3.3 Primary tumor tissue (FFPE) if not previously submitted after randomization on Step 1 as outlined in Section 10 must be available for submission within 4 weeks following registration to Step 3.

Rev. 2/13

4. Randomization Procedures

Step 3 registration to ancillary research study EL112LAB will be via OPEN. See Section 4.11.

Rev. 1/15

CTEP Investigator Registration Procedures

Food and Drug Administration (FDA) regulations and National Cancer Institute (NCI) policy require all investigators participating in any NCI-sponsored clinical trial to register and to renew their registration annually.

Registration requires the submission of:

- a completed ***Statement of Investigator Form*** (FDA Form 1572) with an original signature
- a current Curriculum Vitae (CV)
- a completed and signed ***Supplemental Investigator Data Form*** (IDF)
- a completed ***Financial Disclosure Form*** (FDF) with an original signature

Fillable PDF forms and additional information can be found on the CTEP website at http://ctep.cancer.gov/investigatorResources/investigator_registration.htm. For questions, please contact the CTEP Investigator Registration Help Desk by email at pmbregpend@ctep.nci.nih.gov.

CTEP Associate Registration Procedures / CTEP-IAM Account

The Cancer Therapy Evaluation Program (CTEP) Identity and Access Management (IAM) application is a web-based application intended for use by both Investigators (i.e., all physicians involved in the conduct of NCI-sponsored clinical trials) and Associates (i.e., all staff involved in the conduct of NCI-sponsored clinical trials).

Associates will use the CTEP-IAM application to register (both initial registration and annual re-registration) with CTEP and to obtain a user account.

Investigators will use the CTEP-IAM application to obtain a user account only. (See CTEP Investigator Registration Procedures above for information on registering with CTEP as an Investigator, which must be completed before a CTEP-IAM account can be requested.)

An active CTEP-IAM user account will be needed to access all CTEP and CTSU (Cancer Trials Support Unit) websites and applications, including the CTSU members' website.

Additional information can be found on the CTEP website at http://ctep.cancer.gov/branches/pmb/associate_registration.htm. For questions, please contact the *CTEP Associate Registration Help Desk* by email at ctepreghelp@ctep.nci.nih.gov.

CTSU Registration Procedures

This study is supported by the NCI Cancer Trials Support Unit (CTSU).

IRB Approval:

Each investigator or group of investigators at a clinical site must obtain IRB approval for this protocol and submit IRB approval and supporting documentation to the CTSU Regulatory Office before they can be approved to enroll patients. Study centers can

check the status of their registration packets by querying the Regulatory Support System (RSS) site registration status page of the CTSU members' website by entering credentials at https://www.ctsu.org. For sites under the CIRB initiative, IRB data will automatically load to RSS.

Rev. 1/15 **Downloading Site Registration Documents:**

Site registration forms may be downloaded from the E5103 protocol page located on the CTSU members' website.

- Go to https://www.ctsu.org and log in to the members' area using your CTEP-IAM username and password
- Click on the Protocols tab in the upper left of your screen
- Click on the ECOG-ACRIN link to expand, then select trial protocol E5103
- Click on the Site Registration Documents link

Submitting Regulatory Documents

Before an ECOG-ACRIN Institution may enter patients, protocol specific regulatory documents must be submitted to the CTSU Regulatory Office at the following address:

CTSU Regulatory Office
Coalition of National Cancer Cooperative Groups
1818 Market Street, Suite 1100
Philadelphia, PA 19103
Phone: 1-866-651-2878
FAX: (215) 569-0206
Email: CTSURegulatory@ctsu.coccg.org (for regulatory document submission only)
FAX: (215) 569-0206

Required Protocol Specific Regulatory Documents

1. CTSU Regulatory Transmittal Form.
2. Copy of IRB Informed Consent Document.

   NOTE: Any deletion or substantive modification of information concerning risks or alternative procedures contained in the sample informed consent document must be justified in writing by the investigator and approved by the IRB.

3. A. CTSU IRB Certification Form.
   Or
   B. HHS 310 Form.
   Or
   C. IRB Approval Letter

NOTE: The above submissions must include the following details:

- Indicate all sites approved for the protocol under an assurance number.
- OHRP assurance number of reviewing IRB
- Full protocol title and number
- Version Date
- Type of review (full board vs. expedited)
- Date of review.

- Signature of IRB official

Rev. 1/15 **Checking Your Site's Registration Status:**

Check the status of your site's registration packets by querying the RSS site registration status page of the members' section of the CTSU website. (Note: Sites will not receive formal notification of regulatory approval from the CTSU Regulatory Office.)

- Go to https://www.ctsu.org and log in to the members' area using your CTEP-IAM username and password
- Click on the Regulatory tab at the top of your screen
- Click on the Site Registration tab
- Enter your 5-character CTEP Institution Code and click on Go

The CTSU encourages you to link to the following RSS2.0 webpage so that more information on RSS2.0 as well as the submission forms can be accessed http://www.ctsu.org/rss2_page.asp. If you have questions regarding regulatory document submission, please telephone the CTSU Help Desk at 1-888-823-5923 or E-mail CTSUContact@westat.com. Rev. 5/14

Patients must not start protocol treatment prior to randomization.

Treatment should start within ten working days after randomization.

NOTE: **No blinded starter supplies will be available for the study.** Blinded, patient-specific supplies will be shipped from the Pharmaceutical Management Branch (PMB) to the registering investigator at the time of patient randomization and should arrive within seven to ten days. (See Section 8.1.)

Institutions may register eligible patients to this study via the ECOG webpage 24 hours a day, 7 days a week, using the Web-based Patient Registration Program (https://webreg.ecog.org). If you need assistance or have questions, please telephone the Central Randomization Desk at the ECOG-ACRIN Operations Office - Boston at (617) 632-2022, Monday through Friday 9:00am – 5:00pm Eastern Time. Please note that a password is required to use this program. The following information will be requested:

4.1 <u>STEP 1: Registration to Arm A, Arm B or Arm C</u>

4.1.1 Protocol Number

4.1.2 Investigator Identification

- Institution and affiliate name
- Investigator's name
- NCI investigator ID

4.1.3 Patient Identification

4.1.3.1 Patient's initials and chart number

4.1.3.2 Patient's Social Security number

4.1.3.3 Patient demographics

- Sex
- Birth date (mm/yyyy)

- Race
- Ethnicity
- Nine-digit ZIP code
- Method of payment

NOTE: Patient body weight in kg must be collected at the time of randomization and MUST be transmitted to the Pharmaceutical Management Branch (PMB) as part of the initial order for each patient randomized on the E5103 protocol.

4.2 Eligibility Verification

Patients must meet all of the eligibility requirements listed in Section 3. An eligibility worksheet has been appended to the protocol. A confirmation of registration will be forwarded by the ECOG-ACRIN Operations Office - Boston.

Rev. 7/09

4.3 Stratification Factors

4.3.1 ER status

- ER positive
- ER negative

4.3.2 Lymph Node Involvement

- Negative
- 1-3
- ≥ 4

4.3.3 Type of surgery and planned Radiotherapy

- Breast conservation surgery (Whole breast radiation therapy)
- Breast conservation surgery (Accelerated partial breast radiation pre/post chemotherapy)
- Mastectomy without RT
- Mastectomy with either local or regional RT

4.3.4 AC Schedule Planned

- Classical – every 3 weeks
- Dose dense – every 2 weeks

4.4 Additional Requirements

4.4.1 Patients must provide a signed and dated, written informed consent form.

4.4.2 Biological materials for banking should be submitted as outlined in Section 10.

NOTE: ECOG-ACRIN requires all samples submitted be entered and tracked via the online ECOG-ACRIN Sample Tracking System (STS). See section 10.5.

4.5 Instructions for Patients who Do Not Start Assigned Protocol Treatment

If a patient does not receive any assigned protocol treatment, baseline and follow-up data will still be collected and must be submitted according to the instructions in the E5103 Forms Packet. Document the reason for not starting protocol treatment on the Off-Treatment form. Also report the date and type of the first non-protocol treatment that the patient receives.

4.6 Unblinding Procedures

All patients and treating physicians will be blinded as to assigned treatment group through Cycle 7. All patients and physicians will be unblinded on Day 1 of Cycle 8. Patients randomized to Arm A or Arm B will complete protocol therapy with Cycle 8. Patients randomized to Arm C should be registered to Arm D to continue maintenance bevacizumab treatment for Cycles 9-18. To unblind patients on Cycle 8, day 1, follow the instructions outlined in Appendix XI.

NOTE: Patients will not be unblinded prior to Cycle 8, Day 1.

NOTE: The E5103 Web Unblinding Program is not to be used for cases involving emergency unblinding. Please see Section 4.8 on information for emergency unblinding.

NOTE: No open-label starter supplies will be available for this study. For patients randomized to Arm D, open-label, patient-specific clinical supplies will be shipped from the Pharmaceutical Management Branch (PMB) to the registering investigator at the time patient is registered to Step 2, Arm D, and should arrive within seven to ten days. (See section 8.1.)

NOTE: Patient body weight in kg MUST be collected at the time of unblinding and MUST be transmitted to the Pharmaceutical Management Branch (PMB) as part of the open-label order for each patient scheduled to receive open-label therapy on the E5103 protocol.

4.7 STEP 2: Re-registration to Arm D for Unblinded Patients on Arm C

Only Step 1 patients treated on Arm C, who have not ended treatment per Section 5.7, are eligible to register to Step 2, Arm D, where they will receive an additional 10 doses of bevacizumab.

Rev. 7/09 **NOTE:** Step 2 Arm D treatment must begin no later than 2 months following the last dose of Step 1 bevacizumab.

Rev. 10/09 **NOTE:** For patients registering to Step 2, the PMB will recalculate the drug doses shipped based on the weight reported during the cycle in which the patient was unblinded (not based on the weight reported at baseline).

4.7.1 Protocol Number

4.7.2 Investigator Identification

- Institution and affiliate name
- Investigator’s name
- NCI investigator ID

4.7.3 Patient Identification

4.7.3.1 Patient’s initials and chart number

4.7.3.2 Patient's Social Security number

4.7.3.3 Patient demographics

- Sex
- Birth date (mm/yyyy)
- Race
- Ethnicity
- Nine-digit ZIP code
- Method of payment

NOTE: Patient body weight in kg must be collected at the time of randomization and MUST be transmitted to the Pharmaceutical Management Branch (PMB) as part of the initial order for each patient randomized on the E5103 protocol.

4.8 Emergency Unblinding

Rev. 5/08

NOTE: The information provided below is for the use by a physician, nurse, CRA or pharmacist treating the paitent. These contact numbers should not be used by patients. Patients should be instructed to call their doctor's office in the event of an emergency or adverse event that may result in the need to unblind the patient.

In the event of an emergency or severe adverse reaction necessitating identification of the medication for the welfare of the patient, please contact the Study Chair, Dr. Kathy Miller, at 317-274-1690, first to ensure the reason for unblinding is valid. Then call a member of the ECOG-ACRIN Operations Office - Boston drug team at 617-632-3610 Monday through Friday between 9:00AM and 5:00PM Eastern Time. For unblinding outside of these hours, contact AnswerConnect at 1-866-296-8940. This service will request the reason for unblinding and then page the on-call ECOG-ACRIN staff who will return your call and provide the unblinded treatment assignment if applicable. **Remember, AnswerConnect should only be contacted outside of normal business hours and only in the event of an emergency.** The ECOG-ACRIN Operations Office - Boston or AnswerConnect will require the protocol number (i.e., "E5103"), the patient ID number (e.g., "99999"), and the patient initials (e.g., "FL") to unblind the patient. Please note that if a patient is emergently unblinded he/she is considered to be off-therapy and must discontinue protocol treatment. However, follow-up according to the protocol schedule is still required.

4.9 Reimbursement Guidelines for MUGA/ECHO scans, EKG and Urine Protein Studies

Rev. 7/09

Genentech has agreed to pay for MUGA/ECHO scans, EKG and Urine Protein studies other than standard of care while patients are on protocol treatment. The time points are outlined below and in section 7.0.

NOTE: The baseline MUGA/ECHO and EKG are considered standard of care and should be submitted to the patient's insurance for reimbursement. Genentech will cover the cost for baseline scans for indigent patients.

For subsequent time points, institutions should not submit the bill to the patient or patient's insurer for payment if the invoice is submitted to Genentech for reimbursement.

Rev. 9/10

NOTE: MUGA, ECHOs, or EKGs done as a result of the 24 month post-registration cardiac assessment, as outlined in Sections 7.1.1-7.1.2, are not eligible for reimbursement.

MUGA/ECHO and EKG (baseline is considered standard of care):

Rev. 7/10

Arm	Baseline*	Cycle 5	Cycle 9	Cycle 15	Within 2 weeks of completing RX	1 Year from Step 1 Registration
A and B	X	X			X	X
C**/D	X	X	X	X	X	X**

Standard of care, should be submitted to the patient's insurance for reimbursement

Rev. 9/10

**Arm C patients who do not register to Arm D or who do not begin treatment on Arm D should have follow-up cardiac exams within 2 weeks of completing therapy and 1 year from study entry.

Urine Protein Studies (none are considered standard of care):

Rev. 5/08

Arm	Baseline	Cycle 3	Cycle 5	Cycle 7	Cycle 9	Cycle 12	Cycle 15	Cycle 18
A and B	X	X	X	X				
C/D	X	X	X	X	X	X	X	X

In order for Genentech to authorize payment, the results for the MUGA/ECHO, EKG and Urine Protein Studies must accompany the invoice and the substitute W-9 Tax Form (Appendix XII). These items should be sent to the ECOG-ACRIN Operations Office - Boston, Attn: Drug Orders (fax: 617-632-2063). The ECOG-ACRIN Operations Office - Boston will forward the invoices to Genentech on a quarterly basis.

When submitting an invoice please provide:

1. ECOG-ACRIN Protocol #
2. Provide patient initials and ECOG-ACRIN patient sequence number
3. Name and address of the physician
4. Date of service
5. Institution's CTEP ID code

If you have any questions about this process, please contact a member of the ECOG-ACRIN Drug Team at the ECOG-ACRIN Operations Office - Boston (617-632-3610).

Rev. 7/09

4.10 Guidelines for Technetium-99 Shortages

In the event that you are notified of a technetium-99 supply shortage, follow the instructions below.

1. For patients being worked up for E5103:
   - If a MUGA scan is the preferred method to determine left ventricular ejection fraction (LVEF) then every effort should be made to determine the availability of technetium-99 at your institution.
   - If your institution does not have enough Technetium-99 ECHO's should be used as the preferred assessment for new patients to avoid future changes (i.e. during follow up) in the assessment method.
   - When ECHO is used for baseline scanning, an ECHO must be performed at baseline and every subsequent time-point listed in section 7 of the protocol.
   - It is imperative that a patient does NOT switch from an ECHO to a MUGA during the trial.
   - And for added consistency in interpretation, it is recommended that the ECHOs are conducted at the same facility and read by the same cardiologist if possible.
2. For patients already registered to E5103 (who have already had a baseline MUGA scan):
   - ECOG-ACRIN will allow a +/- 14 day window to obtain the MUGA scans at each of the required time-points. If a follow-up MUGA cannot be obtained with this variance in the time requirement, an ECHO should be substituted.
   - If the ECHO indicates a significant decline in cardiac function, you must contact Kathy Miller, MD, Study Chair, at (317) 274-1690 or kathmill@iupui.edu for further instruction on how to proceed.
   - When the shortage is resolved, these patients can resume assessments using MUGA scans or continue using ECHO scans. This decision is up to the discretion of the Institution.

   Please note that: (1) the provisions for the substitution of ECHOs for MUGAs, and (2) the extended window of time to obtain required MUGAs apply only during the technetium shortage.

Rev. 2/13

4.11 STEP 3: Registration to Ancillary Study EL112LAB

All patients participating E5103 are eligible to participate in this the EL112LAB. Requirements for the ancillary study are outlined in Appendix XV.

Rev. 3/13

NOTE: The ECOG-ACRIN Operations Office - Boston will provide sites with a list of patients who have been identified as potential candidates for EL1112. The information provided will include the date range for which the patients would be eligible to participate, which is dependent on the date of registration to Step 1. Routine notifiers will also be utilized to serve as reminders to sites regarding the timeline of a patient's potential eligibility.

Patient registration can occur only after pre-treatment evaluation is complete, eligibility criteria have been met, and the study site is listed as 'approved' in the CTSU RSS. Patients must have signed and dated all applicable consents and authorization forms.

All site staff (Lead Group and CTSU Sites) will use OPEN to enroll patients to this study. OPEN can be accessed at https://open.ctsu.org or from the OPEN tab on the CTSU members' side of the website at https://www.ctsu.org.

Prior to accessing OPEN site staff should verify the following:

- All eligibility criteria has been met within the protocol stated timeframes. Site staff should use the registration forms provided on the group or CTSU web site as a tool to verify eligibility.
- All patients have signed an appropriate consent form and HIPAA authorization form (if applicable).

Access requirements for OPEN:

- Site staff will need to be registered with CTEP and have a valid and active CTEP-IAM account. This is the same account (user id and password) used for the CTSU members' web site.
- To perform registrations, the site user must have been assigned the 'Registrar' role on the relevant Group or CTSU roster.
- To perform registrations on protocols for which you are a member of the Lead Group, you must have an equivalent 'Registrar' role on the Lead Group roster. Role assignments are handled through the Groups in which you are a member
- To perform registrations to trials accessed via the CTSU mechanism (i.e., non-Lead Group registrations) you must have the role of Registrar on the CTSU roster. Site and/or Data Administrators can manage CTSU roster roles via the new Site Roles maintenance feature under RSS on the CTSU members' web site. This will allow them to assign staff the "Registrar" role.

NOTE: The OPEN system will provide the site with a printable confirmation of registration and treatment information. Please print this confirmation for your records.

Further instructional information is provided on the OPEN tab of the CTSU members' side of the CTSU website at https://www.ctsu.org or at https://open.ctsu.org. For any additional questions contact the CTSU Help Desk at 1-888-823-5923 or ctsucontact@westat.com.

The following information will be collected at time of registration to step 3

4.11.1 Protocol Number

4.11.2 Investigator Identification

- Institution and affiliate name
- Investigator's name
- NCI investigator ID

4.11.3 Patient Identification

4.11.3.1 Patient's initials and chart number

4.11.3.2 Patient's Social Security number

4.11.3.3 Patient demographics

- Sex

- Birth date (mm/yyyy)
- Race
- Ethnicity
- Nine-digit ZIP code
- Method of payment

4.11.4 Additional Requirements

4.11.4.1 Patients must provide a signed and dated, written informed consent form to participate in EL112LAB. The model consent for this ancillary study is provided in Appendix I-A.

4.11.4.2 Forms and biological materials are to be submitted as outlined in Appendix XV. A summy is provided in Section 7.2.2.

NOTE: ECOG-ACRIN requires all samples submitted to be entered and tracked via the online ECOG-ACRIN Sample Tracking System (STS). See Section 10.4.

5. Treatment Plan

Rev. 1/11

NOTE: Please see Sections 5.1.5 and 5.2.5 for information regarding the substitution of epirubicin for doxorubicin in the case of a shortage.

5.1 Administration Schedule – Classical Every 3 Week AC

NOTE: See Section 5.2 for patients receiving dose dense every 2 weeks AC.

NOTE: Patients should begin treatment within ten working days of randomization.

NOTE: **No blinded starter supplies will be available for this study.** Blinded, patient-specific clinical supplies will be shipped from the Pharmaceutical Management Branch (PMB) to the registering investigator at the time of patient randomization and should arrive within seven to ten days. (See Section 8.1.)

Rev. 7/09

NOTE: Treatment start date occurring more than 84 days or ≤ 28 days from date of last surgery (breast conservation surgery, mastectomy, sentinel mode biopsy, axillary dissection or re-excision of breast conservation surgery margins) is considered a protocol violation.

NOTE: If APBI was completed prior to study entry, day 1 of protocol therapy must be at least 4 weeks after the completion of APBI.

NOTE: All patients and treating physicians will be blinded as to assigned treatment group through Cycle 7. All patients and physicians will be unblinded on Day 1 of Cycle 8. (See Section 4.6 for information on Cycle 8, Day 1 unblinding.)

NOTE: See Section 4.9 for MUGA/ECHO, ECG and Urine Protein Studies reimbursement information.

Rev. 7/09

NOTE: The treatment window for both the H and P, labs, and administering treatment should be +/– 2 days from scheduled date. H+P must always be done prior to administering treatment. Patients that are on Dose Dense A/C + Bevacizumab/Placebo with Neulasta should not get their treatment any sooner than 12 days from the administration of Neulasta.

Doses should be based upon actual body weight and not ideal body weight. If subject's weight increases or decreases by ≥ 10% from baseline during the course of the study, the body surface area and drug dose should be recalculated.

5.1.1 STEP 1: ALL TREATMENT GROUPS: Cycles 1-8

Rev. 7/09

AC + Bevacizumab/Placebo (+/– 2 days)

Doxorubicin 60 mg/m^2 plus

Cyclophosphamide 600 mg/m^2 plus

Bevacizumab/Placebo 15 mg/kg **every 21 days x 4**

Followed by:

Rev. 7/09

T + Bevacizumab/Placebo (+/– 2 days)

Paclitaxel 80 mg/m^2 **every 7 days x 12 doses** plus

Bevacizumab/Placebo 15 mg/kg **every 21 days x 4**

Rev. 7/09

5.1.1.1 AC + Bevacizumab/Placebo Administration

AC + Bevacizumab/Placebo will be administered **every 21 days x 4**

Agent	Dose	Route	Day
Doxorubicin	60 mg/m^2	IV push through running IV of NS	1
Cyclophosphamide	600 mg/m^2	IV infusion in 250 mL NS over 20-30 minutes	1
Bevacizumab/Placebo	15 mg/kg	IV infusion	1

Rev. 4/08

NOTE: Patients receiving the classical (every 3 week) schedule of AC should begin paclitaxel 3 weeks after the last AC.

Bevacizumab/Placebo

Rev. 4/08, 5/08

Place the calculated dose of bevacizumab/placebo in 100 mL of 0.9% sodium chloride for injection.

Rate of infusion

The initial Bevacizumab/Placebo dose should be delivered over 90 minutes as a continuous IV infusion. If the first infusion is tolerated without infusion-associated adverse events (fever and/or chills), the second infusion may be delivered over 60 minutes. If the 60-minute infusion is well tolerated, all subsequent infusions may be delivered over 30 minutes.

A rate-regulating device should be used for all Bevacizumab/Placebo infusions. When the Bevacizumab/Placebo IV bag is empty, an additional 50 mL of 0.9% sodium chloride injection should be added to the IV bag and the infusion should be continued for a volume equal to that of the tubing to ensure complete delivery of the Bevacizumab/Placebo. An alternative method of flushing the infusion line would be to replace the empty Bevacizumab/Placebo infusion bag with a 50 mL bag of 0.9% sodium chloride injection and infuse a volume equal to that of the tubing to ensure complete delivery of the Bevacizumab/Placebo. The additional saline flush is not included in the specified infusion times.

If a patient experiences Bevacizumab/Placebo infusion-associated adverse events, the patient may receive premedication at the investigators discretion prior to the next Bevacizumab/Placebo infusion. If premedication is required, the infusion time may not be decreased for the subsequent infusion. However, if the next

infusion is well tolerated with premedication, the subsequent infusion time may then be decreased by 30 minutes per infusion to a minimum infusion time of 30 minutes as long as the subject continues to receive the same premedication.

If a premedicated subject experiences infusion-associated adverse events with the 60-minute infusion, all subsequent doses should be given over 90 minutes. Similarly, if a premedicated subject experiences infusion-associated adverse events with the 30-minute infusion, all subsequent doses should be given over 60 minutes.

Anaphylaxis Precautions

Anaphylaxis precautions should be observed during Bevacizumab/Placebo administration.

Rev. 7/09

- The patient's blood pressure and heartrate should be monitored every 15 minutes during the first infusion of bevacizumab/placebo. If a patient has a reaction, continue blood pressure and heart rate every 15 minutes until stable.
- Emergency agents including oxygen, oral and endotracheal airways, intubation equipment, epinephrine, antihistamines and corticosteroids should be available.
- In the event of a suspected anaphylactic reaction during infusion, stop the Bevacizumab/Placebo infusion and apply a tourniquet proximal to the injection site to slow systemic absorption of Bevacizumab/Placebo. Administer antihistamines, epinephrine, or other medications at the investigators discretion.

Bevacizumab/Placebo Infiltration

Should infiltration of the Bevacizumab/Placebo infusion occur, the following steps are to be taken:

- Discontinue the IV.
- If a significant volume of the Bevacizumab/Placebo infusion remains, restart the IV and complete the infusion.
- Treat the infiltration according to institutional guidelines for infiltration of a noncaustic agent.

5.1.1.2 T + Bevacizumab/Placebo Administration

Paclitaxel will be administered **every 7 days x 12 doses**

Bevacizumab/Placebo will be administered **every 21 days x 4**

NOTE: All patients will be unblinded on Day 1 of Cycle 8. (See section 4.6 for information on Cycle 8, Day 1 unblinding.)

Rev. 5/08

Agent	Time	Dose	Route	Day
Dexamethasone*	30-60 minutes prior to paclitaxel	20 mg	IV or po	Every 7 days
Diphenhydramine*		50 mg	IV or po	
Cimetidine**		300 mg	IV	
Paclitaxel****	NA	80 mg/m^2	IV infusion in 250 mL NS or D_5W over 1 hour	Every 7 days
Bevacizumab/Placebo***	NA	15 mg/kg	IV infusion	Every 21 days

Rev. 7/09

NOTE: Patients receiving the classical (every 3 weeks) schedule of AC should begin Paclitaxel 3 weeks after the last dose of AC.

*The doses of diphenhydramine and dexamethasone may be adjusted after the first dose, based on tolerability.

Rev. 5/08

**Ranitidine (50 mg IV) or famotidine (20 mg IV) may be substituted for cimetidine.

***Bevacizumab/placebo should not be initiated in patients with symptomatic cardiac dysfunction.

Hold bevacizumab/placebo in asymptomatic patients with a decrease in LVEF of a) 10% or more to a value > 6% below the institutions LLN, or b) 16% or more to a value below the institutions LLN. Begin paclitaxel as planned, repeat MUGA/echo in 6 weeks and institute bevacizumab/placebo based on the guidelines in Section 5.5.3.

Rev. 7/09

****Held doses of day 8 or day 15 paclitaxel are considered missed and will not be made up. This is independent from the bevacizumab/placebo dosing during this cycle.

<u>Paclitaxel</u>

Paclitaxel may be diluted in 0.9% sodium chloride injection, USP or 5% dextrose injection, USP. Paclitaxel must be prepared in glass, polypropylene or polyolefin containers and non-PVC-containing (nitroglycerin) infusion sets. In-line filtration with a 0.22 micron filter is required.

<u>Anaphylaxis Precautions</u>

Due to the known potential for allergic reactions to paclitaxel and/or the Cremophor vehicle, the following precautions should be taken to decrease the risk of anaphylaxis.

- 30-60 minutes prior to paclitaxel administration, the patient should be medicated with Dexamethasone 20 mg IV, Diphenhydramine 50 mg IV and Cimetidine (300 mg IV) or other H_2 receptor antagonist.

- Anaphylaxis precautions should be observed during paclitaxel administration. Emergency agents including oxygen, oral and endotracheal airways, intubation equipment, epinephrine, antihistamines and corticosteroids should be available.

Rev. 7/09

5.1.2 STEP 2: Arm D Only – Classical, Every 3 Weeks Bevacizumab Monotherapy (+/- 2 days)

Rev. 7/09

Step 2 Arm D treatment must begin no later than 2 months following the last dose of Step 1 bevacizumab

Bevacizumab will be administered **every 21 days x 10**

Rev. 10/09

NOTE: For patients registering to Step 2, the PMB will recalculate the drug doses shipped based on the weight reported during the cycle in which the patient was unblinded (not based on the weight reported at baseline).

Agent	Dose	Route	Day
Bevacizumab	15 mg/kg	IV infusion	1

5.1.3 Hormonal Therapy

NOTE: For patients in Arm D hormonal therapy will be administered concurrently with bevacizumab. As it is possible that concurrent tamoxifen and bevacizumab may increase the risk of thrombosis, patients should be followed closely. All symptoms and signs of thrombosis must be evaluated promptly.

Rev. 7/09

NOTE: Hormonal therapy must begin within 6 weeks of completing paclitaxel therapy.

Pre-menopausal women: Tamoxifen 20 mg daily for 5 years, should be used in women with ER positive or PR positive tumors. Tamoxifen therapy should be initiated at the time of radiation therapy or within 6 weeks after the completion of chemotherapy for patients not receiving radiation therapy. The use of ovarian ablation or suppression is not recommended but is allowed at the physician’s discretion.

NOTE: **Premenopausal patients may participate in the IBCSG SOFT trial.**

For patients who have received either tamoxifen or raloxifene as chemoprevention before enrolling on this study, the use of tamoxifen following chemotherapy will be at the physician’s discretion.

Post- menopausal women: Patients with ER positive or PR positive tumors may receive tamoxifen (20 mg daily), aromatase inhibitors (anastrozole 1 mg daily, letrozole 2.5 mg daily, or exemestane 25 mg daily), or tamoxifen followed by an aromatase inhibitor. The total duration of adjuvant anti-hormonal therapy should be no more than 10 years.

For patients who have received either tamoxifen or raloxifene as chemoprevention before enrolling on this study, the use of tamoxifen following chemotherapy will be at the physician's discretion.

5.1.4 Radiation Therapy

For all patients receiving whole breast or post-mastectomy radiation, the daily fraction size will be 1.8 or 2.0 grays (Gy) delivered daily Monday through Friday. CT-based treatment planning is strongly encouraged, but not required.

Rev. 7/09

For patients undergoing definitive breast radiation after breast conserving surgery, the timing and method of delivering radiation will be stratified at the time of patient entry into protocol. The options for definitive radiation treatment are: (a) whole breast radiation using standard (conventional) or accelerated fractionation; (b) accelerated partial breast irradiation (APBI) delivered before protocol entry; or (c) APBI delivered after protocol chemotherapy. For patients receiving definitive whole breast radiation treatment after breast conserving surgery, either of the following two dose fractionation schemes is allowed:

a) External beam irradiation to the whole breast to a dose of 45 – 50.4 Gy using daily fractions of 1.8 - 2.0 Gy, 5 days per week, Monday through Friday. A boost dose to the primary tumor bed is recommended to bring the total dose to 60 - 66 Gy.

b) External beam irradiation to the whole breast using accelerated fractionation of 42.56 Gy using daily fractions of 2.66 Gy, in 16 daily fractions, 5 days per week, Monday through Friday. A boost dose to the primary tumor bed of 10 Gy in 4 – 5 fractions is recommended.

For patients undergoing standard whole breast radiation, the initial target volume is the whole breast, which is treated to a dose of 45.0 – 50.4 Gy. A boost to the primary tumor bed is recommended for all patients, but is not required. The boost is typically given to bring the total dose (whole breast plus boost) to 60 – 66 Gy.

Rev. 7/09

For patients undergoing accelerated partial breast irradiation (APBI), enrollment in the randomized trial NSABP B-39/RTOG 0413 (or equivalent randomized clinical trial) is strongly recommended, but not required. If APBI is delivered not on NSABP/RTOG protocol study, it is recommended that the technical radiation treatment be delivered in accordance with this study.

Rev. 7/09

NOTE: APBI delivered prior to protocol entry should be completed at least 4 weeks prior to day 1.

For patients undergoing post mastectomy radiation treatment (PMRT), the minimum target volumes will include the chest wall and the supraclavicular fossa; additional nodal volumes (full axilla and/or internal mammary lymph nodes) may be radiated at the discretion of the individual physician.

Rev. 7/09

For all patients receiving radiation treatment of any kind (definitive whole breast, APBI, or post mastectomy), CT-based treatment planning is strongly encouraged, but not required.

Rev. 5/08

Post-mastectomy RT is required for all patients with a primary tumor of ≥ 5 cm or involvement of 4 or more lymph nodes. Post-mastectomy RT may be administered at the investigator's discretion for all other mastectomy patients

Rev. 7/09

NOTE: For patients randomized to Arm C and registered to Arm D, Radiation therapy is given concurrently wiith bevacizumab and must begin within 6 weeks of completing paclitaxel

Rev. 1/11

5.1.5 Doxorubicin Shortage Guidelines

The following are guidelines for managing the treatment of enrolled participants in E5103, as well as those individuals who have been screened, and future participants, when the doxorubicin supply is not adequate:

5.1.5.1 For enrolled patients (including those who are consented but not yet registered/treated)

Already enrolled participants at institutions who do not have any further doxorubicin can switch to using epirubicin immediately at a starting dose of 90 mg/m².

Dose modifications to epirubicin can be made using the percentage adjustments listed in the table found in Section 5.5.1 of the protocol.

Once an adequate supply of doxorubicin has been obtained the participant should be switched back to an equivalent dose of doxorubicin.

Any participant who underwent a dose modification while receiving epirubicin should resume doxorubicin at an equivalent dose level.

NOTE: As the participants in E5103 are receiving adjuvant therapy, and are potentially curable, and since epirubicin has been shown to be an effective drug as part of an adjuvant therapy regimen for breast cancer, this change can be made immediately as this is a safety issue for these participants. Doing so, however, constitutes an unanticipated event that must be reported to the IRB of record. It is very important to document in the research record that the substitution was due to the doxorubicin shortage.

5.1.5.2 For screened patients (not yet consented) and future patients:

An adequate supply of doxorubicin (defined as 2 cycles worth of doxorubicin) must be available for a potential participant prior to consenting and registering the patient to E5103. Institutions whose doxorubicin supply is not adequate to cover at least 2 cycles of therapy for a potential participant must not consent or register new patients into the study until the doxorubicin supply issue is resolved.

5.2 Administration Schedule – Dose Dense Every 2 Week AC

NOTE: Please see section 5.1 for patients receiving classical every 3 week AC.

NOTE: Patients should begin treatment within ten working days of randomization.

NOTE: **No blinded starter supplies will be available for this study.** Blinded, patient-specific clinical supplies will be shipped from the Pharmaceutical Management Branch (PMB) to the registering investigator at the time of patient randomization and should arrive within seven to ten days. (See section 8.1.)

Rev. 7/09 **NOTE:** Treatment start date occurring more than 84 days or ≤ 28 days from date of last surgery (breast conservation surgery, mastectomy, sentinel mode biopsy, axillary dissection or re-excision of breast conservation surgery margins) is considered a protocol violation.

NOTE: If APBI was completed prior to study entry, day 1 of protocol therapy must be at least 4 weeks after the completion of APBI.

NOTE: All patients and treating physicians will be blinded as to assigned treatment group through Cycle 7. All patients and physicians will be unblinded on Day 1 of Cycle 8. (See section 4.6 for information on Cycle 8, Day 1 unblinding).

NOTE: See section 4.9 for MUGA/ECHO, ECG, and Urine Protein Studies reimbursement information.

Rev. 7/09 **NOTE:** The treatment window for both the H and P, labs, and administering treatment should be +/– 2 days from scheduled date. H+P must always be done prior to administering treatment. Patients that are on Dose Dense A/C + Bevacizumab/Placebo with Neulasta should not get their treatment any sooner than 12 days from the administration of Neulasta.

Rev. 7/09 **Doses should be based upon actual body weight, not ideal body weight. If subject's weight increases or decreases by ≥ 10% from baseline during the course of the study, the body surface area and drug dose should be recalculated.**

Rev. 7/09 5.2.1 STEP I: ALL TREATMENT GROUPS: Cycles 1-8

Rev. 7/09 <u>AC + Bevacizumab/Placebo (+/– 2 days)</u>

Doxorubicin 60 mg/m^2 plus

Cyclophosphamide 600 mg/m^2 plus

Bevacizumab/Placebo 10 mg/kg **every 14 days x 4**

Rev. 7/09 Filgrastim (5 μg/kg SQ) days 2-11 or Pegfilgrastim (6 mg SQ) day 2 of each cycle (required for cycles 1-4 only).

Followed by:

Rev. 7/09 <u>T + Bevacizumab/Placebo (+/– 2 days)</u>

Paclitaxel 80 mg/m^2 **every 7 days x 12 doses** plus

Bevacizumab/Placebo <u>15 mg/kg</u> **every 21 days x 4**

5.2.1.1 AC + Bevacizumab/Placebo Administration

AC + Bevacizumab/Placebo will be administered **every 14 days x 4**

**Agent**	**Dose**	**Route**	**Day**
Doxorubicin	60 mg/m^2	IV push through running IV of NS	1
Cyclophosphamide	600 mg/m^2	IV infusion in 250 mL NS over 20-30 minutes	1
Bevacizumab/placebo	10 mg/kg	IV infusion	1
Pegfilgrastim **OR**	6 mg (regardless of BSA)	SQ	2
Filgrastim	5 μg/kg*		2-11

*Rounded to the nearer of 300 or 480 μg.

NOTE: In the event of a WBC > 50,000/μL or significant bone pain with a WBC > 20,000/μL, the dose of filgrastim may be reduced 50%.

Rev. 4/08 **NOTE:** Patients receiving the dose dense schedule of AC should receive their first dose of paclitaxel 2 weeks after the last AC.

<u>Bevacizumab/Placebo</u>

Rev. 4/08, 5/08 Place the calculated dose of bevacizumab/placebo in 100 mL of 0.9% sodium chloride for injection.

<u>Rate of infusion</u>

The initial Bevacizumab/Placebo dose should be delivered over 90 minutes as a continuous IV infusion. If the first infusion is tolerated without infusion-associated adverse events (fever and/or chills), the second infusion may be delivered over 60 minutes. If the 60-minute infusion is well tolerated, all subsequent infusions may be delivered over 30 minutes.

A rate-regulating device should be used for all Bevacizumab/Placebo infusions. When the Bevacizumab/Placebo IV bag is empty, an additional 50 mL of 0.9% sodium chloride injection should be added to the IV bag and the infusion should be continued for a volume equal to that of the tubing to ensure complete delivery of the Bevacizumab/Placebo. An alternative method of flushing the infusion line would be to replace the empty Bevacizumab/Placebo infusion bag with a 50 mL bag of 0.9% sodium chloride injection and infuse a volume equal to that of the tubing to ensure complete delivery of the Bevacizumab/Placebo. The additional saline flush is not included in the specified infusion times.

If a patient experiences Bevacizumab/Placebo infusion-associated adverse events, the patient may receive premedication at the investigators discretion prior to the next bevacizumab/placebo infusion. If premedication is required, the infusion time may not be decreased for the subsequent infusion. However, if the next infusion is well tolerated with premedication, the subsequent infusion time may then be decreased by 30 minutes per infusion to a minimum infusion time of 30 minutes as long as the subject continues to receive the same premedication.

If a premedicated subject experiences infusion-associated adverse events with the 60-minute infusion, all subsequent doses should be given over 90 minutes. Similarly, if a premedicated subject experiences infusion-associated adverse events with the 30-minute infusion, all subsequent doses should be given over 60 minutes.

Anaphylaxis Precautions

Anaphylaxis precautions should be observed during Bevacizumab/Placebo administration.

Rev. 7/09

- The patient's blood pressure and heartrate should be monitored every 15 minutes during the first infusion of bevacizumab/placebo. If a patient has a reaction, continue blood pressure and heart rate every 15 minutes until stable.
- Emergency agents including oxygen, oral and endotracheal airways, intubation equipment, epinephrine, antihistamines and corticosteroids should be available.
- In the event of a suspected anaphylactic reaction during infusion, stop the Bevacizumab/Placebo infusion and apply a tourniquet proximal to the injection site to slow systemic absorption of Bevacizumab/Placebo. Administer antihistamines, epinephrine, or other medications at the investigators discretion.

Bevacizumab/Placebo Infiltration

Should infiltration of the Bevacizumab/Placebo infusion occur, the following steps are to be taken:

- Discontinue the IV.
- If a significant volume of the Bevacizumab/Placebo infusion remains, restart the IV and complete the infusion.
- Treat the infiltration according to institutional guidelines for infiltration of a noncaustic agent.

Rev. 7/09 5.2.1.2 T + Bevacizumab/Placebo Administration

Paclitaxel will be administered **every 7 days x 12 doses**

Bevacizumab/Placebo will be administered **every 21 days x 4**

NOTE: All patients will be unblinded on Day 1 of Cycle 8. (See section 4.6 for information on Cycle 8, Day 1 unblinding.)

Rev. 5/08

Agent	Time	Dose	Route	Day
Dexamethasone*	30-60 minutes prior to paclitaxel	20 mg	IV or po	Every 7 days
Diphenhydramine*		50 mg	IV or po	
Cimetidine**		300 mg	IV	
Paclitaxel****	NA	80 mg/m^2	IV infusion in 250 mL NS or D_5W over 1 hour	Every 7 days
Bevacizumab/Placebo***	NA	15 mg/kg	IV infusion	Every 21 days

* The doses of diphenhydramine and dexamethasone may be adjusted after the first dose, based on tolerability.

Rev. 5/08

** Ranitidine (50 mg IV) or famotidine (20 mg IV) may be substituted for cimetidine.

*** Bevacizumab/placebo should not be initiated in patients with symptomatic cardiac dysfunction.

Hold bevacizumab/placebo in asymptomatic patients with a decrease in LVEF of a) 10% or more to a value > 6% below the institutions LLN, or b) 16% or more to a value below the institutions LLN. Begin paclitaxel as planned, repeat MUGA/echo in 6 weeks and institute bevacizumab/placebo based on the guidelines in Section 5.5.3.

Rev. 7/09

**** Held doses of day 8 (week 2) or day 15 (week 3) paclitaxel are considered missed and will not be made up. This is independent from the bevacizumab/placebo dosing during this cycle.

Paclitaxel

Paclitaxel may be diluted in 0.9% sodium chloride injection, USP or 5% dextrose injection, USP. Paclitaxel must be prepared in glass, polypropylene or polyolefin containers and non-PVC-

containing (nitroglycerin) infusion sets. In-line filtration with a 0.22 micron filter is required.

Anaphylaxis Precautions

Due to the known potential for allergic reactions to paclitaxel and/or the Cremophor vehicle, the following precautions should be taken to decrease the risk of anaphylaxis.

- 30-60 minutes prior to paclitaxel administration, the patient should be medicated with Dexamethasone 20 mg IV, Diphenhydramine 50 mg IV and Cimetidine (300 mg IV) or other H_2 receptor antagonist.
- Anaphylaxis precautions should be observed during paclitaxel administration. Emergency agents including oxygen, oral and endotracheal airways, intubation equipment, epinephrine, antihistamines and corticosteroids should be available.

5.2.2 STEP 2: Arm D Only: Dose Dense, Every 3 Week Bevacizumab Monotherapy (open label) (+/- 2 days)

Rev. 7/09

Step 2 Arm D treatment must begin no later than 2 months following the last dose of Step 1 bevacizumab

Bevacizumab will be administered **every 21 days x 10**

Rev. 10/09

NOTE: For patients registering to Step 2, the PMB will recalculate the drug doses shipped based on the weight reported during the cycle in which the patient was unblinded (not based on the weight reported at baseline).

Agent	Dose	Route	Day
Bevacizumab	15 mg/kg	IV infusion	1

5.2.3 Hormonal Therapy

NOTE: For patients in Arm D hormonal therapy will be administered concurrently with bevacizumab. As it is possible that concurrent tamoxifen and bevacizumab may increase the risk of thrombosis, patients should be followed closely. All symptoms and signs of thrombosis must be evaluated promptly.

Rev. 7/09

NOTE: Hormonal therapy must begin within 6 weeks of completing paclitaxel therapy.

Pre-menopausal women: Tamoxifen 20 mg daily for 5 years, should be used in women with ER positive or PR positive tumors. Tamoxifen therapy should be initiated at the time of radiation therapy or within 6 weeks of the completion of chemotherapy for patients not receiving radiation therapy. The use of ovarian ablation or suppression is not recommended but is allowed at the physician's discretion.

NOTE: **Premenopausal patients may participate in the IBCSG SOFT trial.**

For patients who have received either tamoxifen or raloxifene as chemoprevention before enrolling on this study, the use of tamoxifen following chemotherapy will be at the physician's discretion.

Post- menopausal women: Patients with ER positive or PR positive tumors may receive tamoxifen (20 mg daily), aromatase inhibitors (anastrozole 1 mg daily, letrozole 2.5 mg daily, or exemestane 25 mg daily), or tamoxifen followed by an aromatase inhibitor. The total duration of adjuvant anti-hormonal therapy should be no more than 10 years.

For patients who have received either tamoxifen or raloxifene as chemoprevention before enrolling on this study, the use of tamoxifen following chemotherapy will be at the physician's discretion.

Rev. 5/08, 7/09

5.2.4 Radiation Therapy

For all patients receiving whole breast or post-mastectomy radiation, the daily fraction size will be 1.8 or 2.0 grays (Gy) delivered daily Monday through Friday. CT-based treatment planning is strongly encouraged, but not required.

For patients undergoing definitive breast radiation after breast conserving surgery, the timing and method of delivering radiation will be stratified at the time of patient entry into protocol. The options for definitive radiation treatment are: (a) whole breast radiation using standard (conventional) or accelerated fractionation; (b) accelerated partial breast irradiation (APBI) delivered before protocol entry; or (c) APBI delivered after protocol chemotherapy. For patients receiving definitive whole breast radiation treatment after breast conserving surgery, either of the following two dose fractionation schemes is allowed:

c) External beam irradiation to the whole breast to a dose of 45 – 50.4 Gy using daily fractions of 1.8 - 2.0 Gy, 5 days per week, Monday through Friday. A boost dose to the primary tumor bed is recommended to bring the total dose to 60 - 66 Gy.

d) External beam irradiation to the whole breast using accelerated fractionation of 42.56 Gy using daily fractions of 2.66 Gy, in 16 daily fractions, 5 days per week, Monday through Friday. A boost dose to the primary tumor bed of 10 Gy in 4 – 5 fractions is recommended.

For patients undergoing standard whole breast radiation, the initial target volume is the whole breast, which is treated to a dose of 45.0 – 50.4 Gy. A boost to the primary tumor bed is recommended for all patients, but is not required. The boost is typically given to bring the total dose (whole breast plus boost) to 60 – 66 Gy.

For patients undergoing accelerated partial breast irradiation (APBI), enrollment in the randomized trial NSABP B-39/RTOG 0413 (or equivalent randomized clinical trial) is strongly recommended, but not required. If APBI is delivered not on NSABP/RTOG protocol study, it

is recommended that the technical radiation treatment be delivered in accordance with this study.

NOTE: APBI delivered prior to protocol entry should be completed at least 4 weeks prior to day 1.

For patients undergoing post mastectomy radiation treatment (PMRT), the minimum target volumes will include the chest wall and the supraclavicular fossa; additional nodal volumes (full axilla and/or internal mammary lymph nodes) may be radiated at the discretion of the individual physician.

For all patients receiving radiation treatment of any kind (definitive whole breast, APBI, or post mastectomy), CT-based treatment planning is strongly encouraged, but not required.

Post-mastectomy RT is required for all patients with a primary tumor of ≥ 5 cm or involvement of 4 or more lymph nodes. Post-mastectomy RT may be administered at the investigator's discretion for all other mastectomy patients

NOTE: For patients randomized to Arm C and registered to Arm D, Radiation therapy is given concurrently wiith bevacizumab and must begin within 6 weeks of completing paclitaxel

Rev. 1/11 5.2.5 Doxorubicin Shortage Guidelines

The following are guidelines for managing the treatment of enrolled participants in E5103, as well as those individuals who have been screened, and future participants, when the doxorubicin supply is not adequate:

5.2.5.1 For enrolled patients (including those who are consented but not yet registered/treated)

Already enrolled participants at institutions who do not have any further doxorubicin can switch to using epirubicin immediately at a starting dose of 90 mg/m².

Dose modifications to epirubicin can be made using the percentage adjustments listed in the table found in Section 5.5.1 of the protocol.

Once an adequate supply of doxorubicin has been obtained the participant should be switched back to an equivalent dose of doxorubicin.

Any participant who underwent a dose modification while receiving epirubicin should resume doxorubicin at an equivalent dose level.

NOTE: As the participants in E5103 are receiving adjuvant therapy, and are potentially curable, and since epirubicin has been shown to be an effective drug as part of an adjuvant therapy regimen for breast cancer, this change can be made immediately as this is a safety issue for these participants. Doing so, however,

constitutes an unanticipated event that must be reported to the IRB of record. It is very important to document in the research record that the substitution was due to the doxorubicin shortage.

5.2.5.2 For screened patients (not yet consented) and future patients:

An adequate supply of doxorubicin (defined as 2 cycles worth of doxorubicin) must be available for a potential participant prior to consenting and registering the patient to E5103. Institutions whose doxorubicin supply is not adequate to cover at least 2 cycles of therapy for a potential participant must not consent or register new patients into the study until the doxorubicin supply issue is resolved.

Rev. 5/14 5.3 Adverse Event Reporting Requirements

5.3.1 **Purpose**

Adverse event data collection and reporting, which are required as part of every clinical trial, are done to ensure the safety of patients enrolled in the studies as well as those who will enroll in future studies using similar agents. Adverse events are reported in a routine manner at scheduled times during a trial (please refer to the E5103 Forms Packet for the list of forms with directions for routine adverse event reporting). Additionally, certain adverse events must be reported in an expedited manner for more timely monitoring of patient safety and care. The following sections provide information about expedited reporting.

5.3.2 **Determination of Reporting Requirements**

Reporting requirements may include the following considerations: 1) whether the patient has received an investigational or commercial agent; 2) the characteristics of the adverse event including the grade (severity), the relationship to the study therapy (attribution), and the prior experience (expectedness) of the adverse event; 3) the phase (1, 2, or 3) of the trial; and 4) whether or not hospitalization or prolongation of hospitalization was associated with the event.

An investigational agent is a protocol drug administered under an Investigational New Drug Application (IND). In some instances, the investigational agent may be available commercially, but is actually being tested for indications not included in the approved package label.

Commercial agents are those agents not provided under an IND but obtained instead from a commercial source. The NCI, rather than a commercial distributor, may on some occasions distribute commercial agents for a trial.

When a study arm includes both investigational and commercial agents, the following rules apply.

Concurrent administration: When an investigational agent(s) is used in combination with a commercial agent(s), the combination is considered to be investigational and expedited reporting of adverse events would follow the guidelines for investigational agents.

Steps to determine if an adverse event is to be reported in an expedited manner:

Rev. 9/11

Step 1: *Identify the type of event:* The descriptions and grading scales found in the revised NCI Common Terminology Criteria for Adverse Events (CTCAE) version 3.0 will be utilized until September 30, 2011 for AE reporting. CTCAE version 4.0 will be utilized beginning October 1, 2011. All appropriate treatment areas should have access to a copy of the CTCAE version 4.0. A copy of the CTCAE version 4.0 can be downloaded from the CTEP web site (http://ctep.cancer.gov).

Step 2: *Grade the event using the NCI CTCAE version 4.0*.

Step 3: *Determine whether the adverse event is related to the protocol therapy (investigational or commercial).* Attribution categories are as follows: Unrelated, Unlikely, Possible, Probable, and Definite.

Step 4: *Determine the prior experience of the adverse event*. *Expected* events are those that have been previously identified as resulting from administration of the agent. An adverse event is considered *unexpected*, for expedited reporting purposes only, when either the type of event or the severity of the event is **NOT** listed in:

Rev. 12/11

- **Arm X (either Arm A B or C) and Arm D** – the current NCI Specific Protocol Exceptions to Expedited Reporting (SPEER) for Bevacizumab or package insert/protocol for the commercial agents

NOTE: The NCI SPEER for Bevacizumab is included in Section 5.4 of the protocol.

- **FOR THIS PROTOCOL**, events listed in the **SPEER** for Bevacizumab should be considered EXPECTED if the grade being reported is the same or lower than the grade noted in the parentheses next to the AE in the SPEER. Events listed in the SPEER column should be considered UNEXPECTED if the grade being reported exceeds the grade noted in parentheses next to the AE in the SPEER.
- If the event being reported is listed in **EITHER** the SPEER for Bevacizumab or the packageinsert/protocol for the commercial agents, then it is considered 'expected' for CTEP-AERS adverse event reporting purposes, regardless of the grade.
- The SPEER is presented in the last column of the CAEPR and identified with ***bold*** and ***italicized*** text.

Step 5: *Review the "Additional instructions, requirements, and exceptions for protocol E5103" table in section 5.3.6 for protocol and/or ECOG-ACRIN specific requirements for expedited reporting of specific adverse events that require special monitoring.*

NOTE: For general questions regarding expedited reporting requirements, please contact the AEMD Help Desk at aemd@tech-res.com or 301-897-7497.

Rev. 10/13
Rev. 1/15

5.3.3 **Reporting Methods**

Arm X (either Arm A B or C) and Arm D – This study requires that expedited adverse event reporting use the NCI's Adverse Reporting System (CTEP-AERS). CTEP's guidelines for CTEP-AERS can be found at http://ctep.cancer.gov. A CTEP-AERS report must be submitted electronically to ECOG and the appropriate regulatory agencies via the CTEP-AERS Web-based application located at http://ctep.cancer.gov.

In the rare event when Internet connectivity is disrupted a 24-hour notification is to be made by telephone to:

- the AE Team at ECOG-ACRIN (617-632-3610)
- the NCI (301-897-7497)

An electronic report MUST be submitted immediately upon re-establishment of internet connection.

Supporting and follow up data: Any supporting or follow up documentation must be faxed to ECOG-ACRIN (617 632 2990), Attention: AE within 48-72 hours. In addition, supporting or follow up documentation must be faxed to the NCI (301- 230-0159) in the same timeframe.

NCI Technical Help Desk: For any technical questions or system problems regarding the use of the CTEP-AERS application, please contact the NCI Technical Help Desk at ncictephelp@ctep.nci.nih.gov or by phone at 1-888-283-7457.

5.3.4 When to Report an Event in an Expedited Manner

Some adverse events require 24-hour notification (refer to Section 5.3.6). Please complete a 24-Hour Notification Report via the NCI
Rev. 1/15
CTEP-AERS website (http://ctep.cancer.gov) within 24 hours of learning of the event. The full CTEP-AERS report must be completed and submitted via CTEP-AERS within 5 calendar days.

If the CTEP-AERS system is down, a 24-hour notification call must be made to ECOG (617-632-3610) and to NCI (301-897-7497). Once the system is restored, a 24-hour Notification Report must be entered into the CTEP-AERS system by the original submitter of the report at the site.

When an adverse event requires expedited reporting, submit a full CTEP-AERS report within the timeframes outlined in Section 5.3.6.

NOTE: Adverse events that meet the reporting requirements in section 5.3.6 and occur within 30 days of the last dose of protocol treatment must be reported on an expedited adverse event report form (using CTEP-AERS). For any adverse events that occur more than 30 days after the last dose of treatment, only those that have an attribution of

possibly, probably, or definitely AND meet the reporting requirements in section 5.3.6 must be reported on an expedited adverse event report form (using CTEP-AERS).

5.3.5 Other Recipients of Adverse Event Reports

DCTD/NCI will notify ECOG-ACRIN/pharmaceutical collaborator(s) of all AE’s reported to the FDA. Any additional written AE information requested by ECOG-ACRIN must be submitted to BOTH the NCI and ECOG-ACRIN.

Adverse events determined to be reportable must also be reported by the institution, according to the local policy and procedures, to the Institutional Review Board responsible for oversight of the patient.

5.3.6 **Expedited Reporting for Investigational Agents**

Phase 2 and 3 Trials Utilizing an Agent under a CTEP IND: CTEP-AERS Expedited Reporting Requirements for Adverse Events That Occur Within 30 Days[1] of the Last Dose of Investigational Agent (Bevacizumab/Placebo) in this Study (Arm X [either Arm A B or C] and Arm D) OR Within 30 Days of the Last Dose of Any Protocol Treatment.

	Grade 1	Grade 2	Grade 2	Grade 3		Grade 3		Grades 4 & 5[2]	Grades 4 & 5[2]
				Unexpected		Expected			
Attribution	Unexpected and Expected	Unexpected	Expected	with Hospitali-zation	without Hospitali-zation	with Hospitali-Zation	without Hospitali-zation	Unexpected	Expected
**Unrelated Unlikely**	Not Required	Not Required	Not Required	10 Calendar Days	Not Required	10 Calendar Days	Not Required	10 Calendar Days	10 Calendar Days
**Possible Probable Definite**	Not Required	10 Calendar Days	Not Required	10 Calendar Days	10 Calendar Days	10 Calendar Days	Not Required	24-Hour; 5 Calendar Days	10 Calendar Days

[1] **Adverse events with attribution of possible, probable, or definite that occur <u>greater</u> than 30 days after the last dose of treatment with an agent under a CTEP IND require reporting as follows:**
CTEP-AERS 24-hour notification followed by complete report within 5 calendar days for:
- Grade 4 and Grade 5 unexpected events

CTEP-AERS 10 calendar day report:
- Grade 3 unexpected events with hospitalization or prolongation of hospitalization
- Grade 5 expected events

[2] Although an CTEP-AERS 24-hour notification is not required for death clearly related to progressive disease, a full report is required as outlined in the table.

Please see additional information below under section entitled “Additional instructions, requirements, and exceptions for protocol E5103”

March 2005

NOTE: All deaths on study require both routine and expedited reporting regardless of causality. Attribution to treatment or other cause should be provided.

- **Expedited AE reporting timelines**:
  - **24 Hours; 5 calendar days** – The investigator must initially report the AE via CTEP-AERS within 24 hours of learning of the event followed by a complete CTEP-AERS report within 5 calendar days of the initial 24-hour report.

- **10 calendar days** – A complete CTEP-AERS report on the AE must be submitted within 10 calendar days of the investigator learning of the event.

- Any medical event equivalent to CTCAE grade 3, 4, or 5 that precipitates **hospitalization* (or prolongation of existing hospitalization)** must be reported regardless of attribution and designation as expected or unexpected with the exception of any events identified as protocol-specific expedited adverse event reporting exclusions.
- Any event that results in **persistent or significant disability/incapacity, congenital anomaly, or birth defect** must be reported via CTEP-AERS if the event occurs following treatment with an agent under a CTEP IND
- Use the NCI protocol number and the protocol-specific patient ID provided during trial registration on all reports.

*Hospitalizations are defined as lasting 24 hours or longer and these events must be reported via CTEP-AERS.

Additional instructions, requirements and exceptions for protocol E5103

1. **Additional Instructions:**
   - With respect to determining the specific day by which the event must be reported, the day the reporter learns of the adverse event constitutes "Day 0."
   - For grade 2 and 3 unexpected events, CTEP-AERS reporting is only required if the event is related to the investigational agent(s); it is not required if the event is related only to the commercial agent(s) included in the protocol treatment.

     NOTE: For grade 3 unexpected events with hospitalization lasting ≥ 24 hours (or prolonged hospitalization), an CTEP-AERS report is required even if the event is unrelated to the investigational agent(s).
   - For instructions on how to specifically report events that result in persistent or significant disability/incapacity, congenital anomaly, or birth defect events via CTEP-AERS, please contact the AEMD Help Desk at aemd@tech-res.com or 301-897-7497.
2. **ECOG-ACRIN and Protocol Specific expedited reporting requirements:**

   The adverse events listed below also require expedited reporting for this trial:

   ECOG-ACRIN specific expedited reporting requirements:

   - **Hospitalizations:** Any grade 1 or 2 adverse event which precipitates a hospitalization lasting ≥ 24 hours (or prolongs hospitalization) must be reported via CTEP-AERS within 10 calendar days of learning of the event regardless of the attribution and designation as expected or unexpected.

   Protocol specific expedited reporting requirements:

   - **CHF:** Any ≥ grade 3 CHF must be reported via CTEP-AERS within 10 calendar days of learning of the event regardless of the attribution.
   - **LVEF:** Any decrease in LVEF to below the lower limit of normal from the baseline timepoint must be reported via CTEP-AERS within 10 calendar days of learning of the event regardless of attribution.

   RPLS or PRES: All occurrences of **Reversible Posterior Leukoencephalopathy Syndrome (RPLS) or Posterior Reversible Encephalopathy Syndrome (PRES)** and associated clinical presentations [please report under Neurology – Other (Leukoencephalopathy Syndrome)] must be submitted within 10 calendar days of learning of the event, regardless of attribution.
3. **Protocol specific expedited reporting exceptions:**

   For study arms x (either Arm A B or C) and Arm D, the adverse events listed below **do not** require expedited reporting via CTEP-AERS:

- Grade 4 expected **myelosuppression** (unless it results in a hospitalization, in which case, an CTEP-AERS report is required).
- ≥ Grade 3 Nausea, regardless of the need for hospitalization
- ≥ Grade 3 Vomiting, regardless of the need for hospitalization
- ≥ Grade 3 Dehydration, regardless of the need for hospitalization

Rev. 7/09
NOTE: Grade 4 **neutropenia** does NOT require expedited reporting via CTEP-AERS, even if the patient is hospitalized.

Rev. 10/13

5.3.7 Reporting Secondary AML/MDS/ALL

All cases of second primary cancers, including acute myeloid leukemia (AML) and myelodysplastic syndrome (MDS), that occur following treatment on NCI-sponsored trials must be reported to ECOG-ACRIN:

- **A second malignancy is a cancer that is UNRELATED to any prior anti-cancer treatment (including the treatment on this protocol). Second malignancies require ONLY routine reporting as follows:**

  1. Submit a completed Second Primary Form within 30 days to ECOG-ACRIN at

     ECOG-ACRIN Operations Office - Boston
     FSTRF
     900 Commonwealth Avenue
     Boston, MA 02215

  2. Submit a copy of the pathology report to ECOG-ACRIN confirming the diagnosis.
  3. If the patient has been diagnosed with AML/MDS, submit a copy of the cytogenetics report (if available) to ECOG-ACRIN

- **A secondary malignancy is a cancer CAUSED BY any prior anti-cancer treatment (including the treatment on this protocol). Secondary malignancies require both routine and expedited reporting as follows:**

  1. Submit a completed Second Primary Form within 30 days to ECOG-ACRIN at

     ECOG-ACRIN Operations Office - Boston
     FSTRF
     900 Commonwealth Avenue
     Boston, MA 02215

  2. Report the diagnosis via CTEP-AERS at http://ctep.cancer.gov

     Report under 'Secondary Malignancy – Other'

  3. Submit a copy of the pathology report to ECOG-ACRIN and NCI/CTEP confirming the diagnosis.

4. If the patient has been diagnosed with AML/MDS, submit a copy of the cytogenetics report (if available) to ECOG-ACRIN and NCI/CTEP.

NOTE: The Second Primary Form and the CTEP-AERS report should not be used to report recurrence or development of metastatic disease.

NOTE: If a patient has been enrolled in more than one NCI-sponsored study, the Second Primary Form must be submitted for the most recent trial. ECOG-ACRIN must be provided with a copy of the form and the associated pathology report and cytogenetics report (if available) even if ECOG-ACRIN was not the patient's most recent trial.

NOTE: Once data regarding survival and remission status are no longer required by the protocol, no follow-up data should be submitted via CTEP-AERS or by the Second Primary Form.

Rev. 9/10, 12/11

5.4 Comprehensive Adverse Events and Potential Risks list (CAEPR) for Bevacizumab (rhuMAb VEGF, NSC 704865)

The Comprehensive Adverse Event and Potential Risks list (CAEPR) provides a single list of reported and/or potential adverse events (AE) associated with an agent using a uniform presentation of events by body system. In addition to the comprehensive list, a subset, the Specific Protocol Exceptions to Expedited Reporting (SPEER), appears in a separate column and is identified with ***bold*** and ***italicized*** text.

Rev. 1/15

NOTE: FOR THIS PROTOCOL, events listed in the SPEER column should be considered EXPECTED if the grade being reported is the same or lower than the grade noted in the parentheses next to the AE in the SPEER. Events listed in the SPEER column should be considered UNEXPECTED if the grade being reported exceeds the grade noted in parentheses next to the AE in the SPEER.

Rev. 1/15

NOTE: If the event being reported is listed in **EITHER** the SPEER for Bevacizumab or the packageinsert/protocol for the commercial agents, then it is considered 'expected' for CTEP-AERS adverse event reporting purposes, regardless of the grade.

Version 2.3, August 1, 2013[1]

**Adverse Events with Possible Relationship to Bevacizumab (rhuMAb VEGF) (CTCAE 4.0 Term) [n= 3540]**			**Specific Protocol Exceptions to Expedited Reporting (SPEER)**
**Likely (>20%)**	**Less Likely (<=20%)**	**Rare but Serious (<3%)**	
BLOOD AND LYMPHATIC SYSTEM DISORDERS			
	Anemia		***Anemia (Gr 3)***
		Blood and lymphatic system disorders - Other (renal thrombotic microangiopathy)	
	Febrile neutropenia		***Febrile neutropenia (Gr 3)***
CARDIAC DISORDERS			
		Acute coronary syndrome[2]	
	Cardiac disorders - Other (supraventricular arrhythmias)[3]		***Cardiac disorders - Other (supraventricular arrhythmias)[3] (Gr 3)***
		Heart failure	
		Left ventricular systolic dysfunction	
		Myocardial infarction[2]	
		Ventricular arrhythmia	
		Ventricular fibrillation	

GASTROINTESTINAL DISORDERS			
	Abdominal pain		***Abdominal pain (Gr 3)***
	Colitis		***Colitis (Gr 3)***
	Constipation		***Constipation (Gr 3)***
	Diarrhea		***Diarrhea (Gr 3)***
	Dyspepsia		***Dyspepsia (Gr 2)***
		Gastrointestinal fistula[4]	
	Gastrointestinal hemorrhage[5]		***Gastrointestinal hemorrhage[5] (Gr 2)***
	Gastrointestinal obstruction[6]		
		Gastrointestinal perforation[7]	
		Gastrointestinal ulcer[8]	
	Ileus		
	Mucositis oral		***Mucositis oral (Gr 3)***
	Nausea		***Nausea (Gr 3)***
	Vomiting		***Vomiting (Gr 3)***
GENERAL DISORDERS AND ADMINISTRATION SITE CONDITIONS			
	Fatigue		***Fatigue (Gr 3)***
	Infusion related reaction		***Infusion related reaction (Gr 2)***
	Non-cardiac chest pain		***Non-cardiac chest pain (Gr 3)***
	Pain		***Pain (Gr 3)***
IMMUNE SYSTEM DISORDERS			
	Allergic reaction		***Allergic reaction (Gr 2)***
		Anaphylaxis	
INFECTIONS AND INFESTATIONS			
	Infection[9]		***Infection[9] (Gr 3)***
		Infections and infestations - Other (necrotizing fasciitis)	
	Infections and infestations - Other (peri-rectal abscess)		
INJURY, POISONING AND PROCEDURAL COMPLICATIONS			
		Injury, poisoning and procedural complications – Other (anastomotic leak)[10]	
	Wound complication		***Wound complication (Gr 2)***
	Wound dehiscence		***Wound dehiscence (Gr 2)***
INVESTIGATIONS			
	Alanine aminotransferase increased		***Alanine aminotransferase increased (Gr 3)***
	Alkaline phosphatase increased		***Alkaline phosphatase increased (Gr 3)***

	Aspartate aminotransferase increased		***Aspartate aminotransferase increased (Gr 3)***
	Blood bilirubin increased		***Blood bilirubin increased (Gr 2)***
	Cardiac troponin I increased		
Neutrophil count decreased			***Neutrophil count decreased (Gr 3)***
	Platelet count decreased		***Platelet count decreased (Gr 4)***
	Weight loss		***Weight loss (Gr 3)***
	White blood cell decreased		***White blood cell decreased (Gr 3)***
METABOLISM AND NUTRITION DISORDERS			
	Anorexia		***Anorexia (Gr 3)***
	Dehydration		***Dehydration (Gr 3)***
MUSCULOSKELETAL AND CONNECTIVE TISSUE DISORDERS			
	Arthralgia		***Arthralgia (Gr 3)***
	Musculoskeletal and connective tissue disorder - Other (bone metaphyseal dysplasia)[11]		
	Myalgia		***Myalgia (Gr 3)***
	Osteonecrosis of jaw[12]		
NERVOUS SYSTEM DISORDERS			
	Dizziness		***Dizziness (Gr 2)***
	Headache		***Headache (Gr 3)***
		Intracranial hemorrhage	
		Ischemia cerebrovascular[2]	
	Peripheral sensory neuropathy[13]		
		Reversible posterior leukoencephalopathy syndrome	
	Syncope		
RENAL AND URINARY DISORDERS			
		Acute kidney injury	
	Hematuria		***Hematuria (Gr 3)***
	Proteinuria		***Proteinuria (Gr 2)***
		Renal and urinary disorders - Other (Nephrotic Syndrome)	
		Urinary fistula	
REPRODUCTIVE SYSTEM AND BREAST DISORDERS			
Reproductive system and breast disorders - Other (ovarian failure)[14]			
		Vaginal fistula	
	Vaginal hemorrhage		***Vaginal hemorrhage (Gr 3)***

RESPIRATORY, THORACIC AND MEDIASTINAL DISORDERS			
	Allergic rhinitis		***Allergic rhinitis (Gr 3)***
		Bronchopleural fistula	
		Bronchopulmonary hemorrhage	
	Cough		***Cough (Gr 3)***
	Dyspnea		***Dyspnea (Gr 2)***
	Epistaxis		***Epistaxis (Gr 3)***
	Hoarseness		***Hoarseness (Gr 3)***
		Respiratory, thoracic and mediastinal disorders - Other (nasal-septal perforation)	
		Respiratory, thoracic and mediastinal disorders - Other (tracheo-esophageal fistula)	
SKIN AND SUBCUTANEOUS TISSUE DISORDERS			
	Pruritus		***Pruritus (Gr 2)***
	Rash maculo-papular		***Rash maculo-papular (Gr 2)***
	Urticaria		***Urticaria (Gr 2)***
VASCULAR DISORDERS			
Hypertension			***Hypertension (Gr 3)***
	Thromboembolic event		***Thromboembolic event (Gr 3)***
		Vascular disorders - Other (arterial thromboembolic event)[2,15]	

[1] This table will be updated as the toxicity profile of the agent is revised. Updates will be distributed to all Principal Investigators at the time of revision. The current version can be obtained by contacting PIO@CTEP.NCI.NIH.GOV. Your name, the name of the investigator, the protocol and the agent should be included in the e-mail.

[2] The risks of arterial thrombosis such as cardiac or CNS ischemia are increased in elderly patients and in patients with a history of diabetes.

[3] Supraventricular arrhythmias may include supraventricular tachycardia, atrial fibrillation and atrial flutter.

[4] Gastrointestinal fistula may include: Anal fistula, Colonic fistula, Duodenal fistula, Esophageal fistula, Gastric fistula, Gastrointestinal fistula, Rectal fistula, and other sites under the GASTROINTESTINAL DISORDERS SOC.

[5] Gastrointestinal hemorrhage may include: Colonic hemorrhage, Duodenal hemorrhage, Esophageal hemorrhage, Esophageal varices hemorrhage, Gastric hemorrhage, Hemorrhoidal hemorrhage, Intra-abdominal hemorrhage, Oral hemorrhage, Rectal hemorrhage, and other sites under the GASTROINTESTINAL DISORDERS SOC.

[6] Gastrointestinal obstruction may include: Colonic obstruction, Duodenal obstruction, Esophageal obstruction, Ileal obstruction, Jejunal obstruction, Rectal obstruction, Small intestinal obstruction, and other sites under the GASTROINTESTINAL DISORDERS SOC.

[7] Gastrointestinal perforation may include: Colonic perforation, Duodenal perforation, Esophageal perforation, Gastric perforation, Jejunal perforation, Rectal perforation, Small intestinal perforation, and other sites under the GASTROINTESTINAL DISORDERS SOC.

[8] Gastrointestinal ulcer may include: Duodenal ulcer, Esophageal ulcer, Gastric ulcer, and other sites under the GASTROINTESTINAL DISORDERS SOC.

[9] Infection may include any of the 75 infection sites under the INFECTIONS AND INFESTATIONS SOC.

[10] Anastomotic leak may include Gastric anastomotic leak; Gastrointestinal anastomotic leak; Large intestinal anastomotic leak; Rectal anastomotic leak; Small intestinal anastomotic leak; Urostomy leak; Vaginal anastomotic leak

[11] Metaphyseal dysplasia was observed in young patients who still have active epiphyseal growth plates.

[12] Cases of osteonecrosis of the jaw (ONJ) have been reported in cancer patients in association with bevacizumab treatment, the majority of whom had received prior or concomitant treatment with i.v. bisphosphonates.

[13] Increased rate of peripheral sensory neuropathy has been observed in trials combining bevacizumab and chemotherapy compared to chemotherapy alone.

[14] Ovarian failure, defined as amenorrhea lasting 3 or more months with follicle-stimulating hormone (FSH) elevation (≥30 mIU/mL), was increased in patients receiving adjuvant bevacizumab plus mFOLFOX compared to mFOLFOX alone (34% vs. 2%). After discontinuation of bevacizumab, resumption of menses and an FSH level <30 mIU/mL was demonstrated in 22% (7/32) of these women. Long term effects of bevacizumab exposure on fertility are unknown.

[15] Arterial thromboembolic event includes visceral arterial ischemia, peripheral arterial ischemia, heart attack and stroke.

Also reported on bevacizumab (rhuMAb VEGF) trials but with the relationship to bevacizumab (rhuMAb VEGF) still undetermined:

BLOOD AND LYMPHATIC SYSTEM DISORDERS - Blood and lymphatic system disorders - Other (idiopathic thrombocytopenia purpura); Bone marrow hypocellular; Disseminated intravascular coagulation; Hemolysis

CARDIAC DISORDERS - Atrioventricular block complete; Atrioventricular block first degree; Cardiac arrest; Myocarditis; Pericardial effusion; Restrictive cardiomyopathy; Right ventricular dysfunction

EAR AND LABYRINTH DISORDERS - Ear and labyrinth disorders - Other (tympanic membrane perforation); Hearing impaired; Tinnitus; Vertigo

ENDOCRINE DISORDERS - Hyperthyroidism; Hypothyroidism

EYE DISORDERS - Blurred vision; Cataract; Dry eye; Extraocular muscle paresis; Eye disorders - Other (blindness); Eye disorders - Other (conjunctival hemorrhage); Eye disorders - Other (corneal epithelial defect); Eye disorders - Other (floaters); Eye disorders - Other (ischemic CRVO); Eye disorders - Other (macular pucker); Eye disorders - Other (transient increased IOP > or =30 mm Hg); Eye disorders - Other (vitreous hemorrhage); Eye pain; Keratitis; Optic nerve disorder; Photophobia; Retinal detachment; Retinal tear; Retinopathy; Watering eyes

GASTROINTESTINAL DISORDERS - Ascites; Chelitis; Colonic stenosis; Dry mouth; Dysphagia; Enterocolitis; Esophageal pain; Esophageal stenosis; Flatulence; Gastrointestinal disorders - Other (peritonitis); Oral pain; Pancreatitis; Proctitis; Rectal mucositis; Rectal stenosis;Typhlitis

GENERAL DISORDERS AND ADMINISTRATION SITE CONDITIONS - Death NOS; Edema face; Edema limbs; Edema trunk; Facial pain; Fever; Flu like symptoms; Gait disturbance; Injection site reaction; Localized edema; Multi-organ failure; Sudden death NOS

HEPATOBILIARY DISORDERS - Cholecystitis; Gallbladder necrosis; Gallbladder obstruction; Hepatic failure; Hepatic necrosis

INFECTIONS AND INFESTATIONS - Infections and infestations - Other (aseptic meningitis)

INJURY, POISONING AND PROCEDURAL COMPLICATIONS - Arterial injury; Bruising; Burn; Dermatitis radiation; Fracture

INVESTIGATIONS - Activated partial thromboplastin time prolonged; Blood antidiuretic hormone abnormal; CD4 lymphocytes decreased; CPK increased; Carbon monoxide diffusing capacity decreased; Electrocardiogram QT corrected interval prolonged; Forced expiratory volume decreased; GGT increased; INR increased; Lipase increased; Lymphocyte count decreased; Serum amylase increased; Weight gain

METABOLISM AND NUTRITION DISORDERS - Acidosis; Hypercalcemia; Hyperglycemia; Hyperkalemia; Hypermagnesemia; Hypernatremia; Hypertriglyceridemia; Hyperuricemia; Hypoalbuminemia; Hypocalcemia; Hypokalemia; Hypomagnesemia; Hyponatremia; Hypophosphatemia

MUSCULOSKELETAL AND CONNECTIVE TISSUE DISORDERS - Arthritis; Back pain; Bone pain; Chest wall pain; Fibrosis deep connective tissue; Generalized muscle weakness; Head soft tissue necrosis; Joint effusion; Muscle weakness lower limb; Muscle weakness upper limb; Musculoskeletal and connective tissue disorder - Other (aseptic necrotic bone); Musculoskeletal and connective tissue disorder - Other (myasthenia gravis); Musculoskeletal and connective tissue disorder - Other (polymyalgia rheumatica); Neck pain; Pain in extremity; Pelvic soft tissue necrosis; Soft tissue necrosis lower limb

NEOPLASMS BENIGN, MALIGNANT AND UNSPECIFIED (INCL CYSTS AND POLYPS) - Tumor pain

NERVOUS SYSTEM DISORDERS - Arachnoiditis; Ataxia; Central nervous system necrosis; Cerebrospinal fluid leakage; Cognitive disturbance; Depressed level of consciousness; Dysesthesia; Dysgeusia; Dysphasia; Encephalopathy; Extrapyramidal disorder; Facial nerve disorder; Hydrocephalus; Leukoencephalopathy; Memory impairment; Nervous system disorders - Other (increased intracranial pressure); Paresthesia; Peripheral motor neuropathy; Pyramidal tract syndrome; Seizure; Somnolence; Tremor; Vasovagal reaction

PSYCHIATRIC DISORDERS - Agitation; Anxiety; Confusion; Depression; Insomnia; Libido decreased; Psychosis

RENAL AND URINARY DISORDERS - Bladder spasm; Chronic kidney disease; Cystitis noninfective; Renal and urinary disorders - Other (dysuria); Renal and urinary disorders - Other (ureterolithiasis); Renal hemorrhage; Urinary frequency; Urinary incontinence; Urinary retention; Urinary tract obstruction; Urinary tract pain

REPRODUCTIVE SYSTEM AND BREAST DISORDERS - Breast pain; Erectile dysfunction; Irregular menstruation; Pelvic pain; Vaginal discharge

RESPIRATORY, THORACIC AND MEDIASTINAL DISORDERS - Adult respiratory distress syndrome; Atelectasis; Hypoxia; Nasal congestion; Pulmonary fibrosis; Pulmonary hypertension; Respiratory failure; Respiratory, thoracic and mediastinal disorders - Other (dry nares); Respiratory, thoracic and mediastinal disorders - Other (pulmonary infarction)

SKIN AND SUBCUTANEOUS TISSUE DISORDERS - Alopecia; Dry skin; Hyperhidrosis; Nail loss; Pain of skin; Palmar-plantar erythrodysesthesia syndrome; Photosensitivity; Purpura; Rash acneiform; Skin and subcutaneous tissue disorders - Other (diabetic foot ulcer); Skin and subcutaneous tissue disorders - Other (skin breakdown/ decubitus ulcer); Skin hyperpigmentation; Skin induration; Skin ulceration; Stevens-Johnson syndrome

VASCULAR DISORDERS - Flushing; Hot flashes; Hypotension; Lymphocele; Phlebitis; Vasculitis

NOTE: Bevacizumab (rhuMAb VEGF) in combination with other agents could cause an exacerbation of any adverse event currently known to be caused by the other agent, or the combination may result in events never previously associated with either agent.

Rev. 7/09

5.5 Dose Modifications

Rev. 9/11

All toxicities should be graded according to the NCI Common Terminology Criteria for Adverse Events (CTCAE) version 4.0.

The CTCAE version 4.0 is identified and located on the CTEP website at http://ctep.cancer.gov. All appropriate treatment areas should have access to a copy of the CTCAE version 4.0.

If a patient develops multiple toxicities, delay treatment or modify dose based on the greatest indicated dose reduction or delay. Dose re-escalations are not allowed.

Rev. 1/11

NOTE: Dose modifications for patients on epirubicin can be made using the percentage adjustments in the table below.

5.5.1 AC Dose Modifications

**Event**	**Dose Modification**
**Neutropenia**	
> 1000/mm^3	No change
≤ 1000/mm^3	Hold until ANC >1000, resume based on timing of recovery:<br>≤ 1 week – no change<br>>1 but ≤ 3 weeks - reduce dose 20% for subsequent cycles<br>> 3 weeks – stop AC, proceed to paclitaxel
**Neutropenic Fever**	
ANC≤ 1000,<br>fever ≥ 38.5	Interrupt until resolved (ANC >1000, fever <38.5), resume according to number of episodes:<br>1st = no change<br>2nd = 20% dose reduction<br>3rd = stop AC, proceed with paclitaxel
**Thrombocytopenia**	
≥100,000/mm^3	No change
75-99,999/mm^3	Hold until ≥ 100,000, resume based on timing of recovery:<br>≤ 1 week – no change<br>>1 but ≤ 3 weeks - reduce dose 20% for subsequent cycles<br>> 3 weeks – stop AC, proceed to paclitaxel
<75,000	Hold until ≥100,000, Resume with 20% dose reduction for subsequent cycles. If > 3 weeks delay is required, stop AC and proceed with paclitaxel.
**Anemia**	
All grades	No change – erythropoietin or darbepoietin therapy may be initiated at the investigator's discretion.
**Hepatic**	
Grade 0 or 1	No change
≥ Grade 2	Interrupt until ≤ Grade 1, then resume previous dose. If > 3 weeks delay is required, stop AC and proceed with paclitaxel.
**Nausea/Vomiting**	

Grade 0 - 2	No change
≥ Grade 3	Hold until resolved to ≤ Grade 1, reduce dose 20% in subsequent cycles.
**Mucositis**	
Grade 0 - 2	No change
≥ Grade 3	Hold until resolved to ≤ Grade 1, reduce dose 20% in subsequent cycles.
**Cardiac**	
Grade 0 - 2	No change
≥ Grade 3	Discontinue AC if:<br>o a patient has symptoms of CHF and a diagnosis of CHF is confirmed<br>o a patient has a myocardial infarction<br>o >15% absolute decline in LVEF from baseline, or >10% decline in LVEF from baseline to below LLN<br>Patients who discontinue AC due to cardiac toxicity may receive paclitaxel at the investigator's discretion but bevacizumab/placebo should NOT be administered.<br>**NOTE:** PACs or PVCs without cardiac dysfunction (e.g., acute dysrhythmias) during and shortly after doxorubicin infusion are NOT an indication to permanently stop doxorubicin.
**Other clinically significant toxicity excluding fatigue, alopecia and leukopenia**	
Grade 0 or 1	No change
Grade 2	Hold until resolved to ≤ Grade 1, resume at previous dose. Increase supportive care measures if possible.
≥ Grade 3	Hold until resolved to ≤ Grade 1, resume with 20% dose reduction for subsequent cycles. If Grade 3 or greater toxicity recurs, stop AC and proceed with paclitaxel.

Rev. 5/08

5.5.2 Paclitaxel Dose Modifications

AC dose modification or delay will not impact paclitaxel therapy.

Rev. 7/09

NOTE: Day 1 of paclitaxel may be delayed. However, held doses on Day 8 (week 2) and Day 15 (week 3) are considered missed doses and will not be made up.

Rev. 7/09

NOTE: If paclitaxel is not administered for > 3 weeks due to toxicity, stop paclitaxel and continue with bevacizumab/placebo.

Rev. 7/10

Event	Dose Modification
**Neutropenia**	
> 1000/mm^3	No change
≤ 1000/mm^3	Hold until ANC >1000, resume based on timing of recovery:<br>≤ 1 week – no change<br>>1 but ≤ 3 weeks - reduce dose 20% for subsequent cycles<br>> 3 weeks – stop paclitaxel, continue bevacizumab/placebo
**Neutropenic Fever**	
ANC ≤ 1000,<br>fever ≥ 38.5	Interrupt until resolved (ANC >1000, fever <38.5), resume according to number of episodes:<br>1st = no change<br>2nd = 20% dose reduction<br>3rd = stop paclitaxel, continue bevacizumab/placebo
**Thrombocytopenia**	
≥ 100,000/mm^3	No change
75-99,999/mm^3	Hold until ≥ 100,000, resume based on timing of recovery:<br>≤ 1 week – no change<br>>1 but ≤ 3 weeks - reduce dose 20% for subsequent cycles<br>> 3 weeks – stop paclitaxel, continue bevacizumab/placebo
<75,000	Hold until ≥ 100,000, Resume with 20% dose reduction for subsequent cycles. If > 3 weeks delay is required, stop paclitaxel and continue bevacizumab/placebo
**Anemia**	
All grades	No change – erythropoietin or darbepoietin therapy may be initiated at the investigator’s discretion.
**Hepatic**	
Grade 0 or 1	No change
≥ Grade 2	Interrupt until ≤ Grade 1, then resume previous dose. If > 3 weeks delay is required, stop paclitaxel and continue bevacizumab/placebo
**Nausea/Vomiting**	
Grade 0 - 2	No change
≥ Grade 3	Hold until resolved to ≤ Grade 1, reduce dose 20% in subsequent cycles.

**Mucositis**	
Grade 0 - 2	No change
≥ Grade 3	Hold until resolved to ≤ Grade 1, reduce dose 20% in subsequent cycles.
**Neurotoxicity**	
Grade 0 - 1	No change
Grade 2	If Grade 2 toxicity has resolved to ≤ Grade 1 on the day of treatment, proceed with treatment at the previous dose.<br>If Grade 2 toxicity is present on the day of treatment, reduce dose 20% for all subsequent cycles.
Grade 3	Hold until resolved to ≤ Grade 1, reduce dose 20% in all subsequent cycles. If > 3 weeks delay is required, stop paclitaxel and continue bevacizumab/placebo.
Grade 4	Discontinue paclitaxel and continue bevacizumab/placebo.
**Anaphylaxis/Hypersensitivity**	
Mild (e.g., mild flushing, rash, pruritis)	No treatment needed. Supervise at bedside and complete paclitaxel infusion.
Moderate (e.g., moderate flushing, rash, mild dyspnea, chest discomfort)	Stop paclitaxel. Administer diphenhydramine 25 mg and dexamethasone 10 mg IV. After recovery, resume infusion at half the previous rate for 15 minutes. If no further symptoms occur, complete the infusion at the full dose rate. If symptoms recur, stop paclitaxel. Patients may continue bevacizumab/placebo.
Severe (e.g., hypotension requiring pressors, angioedema, respiratory distress requiring bronchdilators)	Stop paclitaxel. Administer diphenhydramine 25 mg and dexamethasone 10 mg IV. Add epinephrine or bronchodilators as needed. Patients may continue bevacizumab/placebo. Do not restart paclitaxel.
**Other clinically significant toxicity excluding fatigue, alopecia and leukopenia**	
Grade 0 or 1	No change
Grade 2	Hold until resolved to ≤ Grade 1, resume at previous dose. Increase supportive care measures if possible.
≥ Grade 3	Hold until resolved to ≤ Grade 1, resume with 20% dose reduction for subsequent cycles. If Grade 3 or greater toxicity recurs, stop paclitaxel and continue bevacizumab/placebo.

Rev. 5/08

5.5.3 Bevacizumab/Placebo Dose Modifications

Dose reduction of AC and/or paclitaxel will not impact bevacizumab/placebo dose. However, if a chemotherapy cycle is delayed, bevacizumab/placebo therapy should be delayed to maintain concurrent administration. If chemotherapy is permanently discontinued due to toxicity, patients may complete therapy with bevacizumab/placebo alone.

There are no reductions in the bevacizumab/placebo dose. If adverse events occur that require holding bevacizumab/placebo, the dose will remain the same once treatment resumes. Treatment may be held on more than one occasion, but regardless of the reason for holding study drug treatment, the maximum allowable length of continuous treatment interruption is 2 months.

NOTE: Held doses of bevacizumab/placebo are considered missed and will not be made up.

Rev. 7/09

Rev. 7/09

<table>
<tr><th>Event</th><th colspan="3">Dose Modification</th></tr>
<tr><td colspan="4">Asymptomatic Decrease in LVEF</td></tr>
<tr><td colspan="4">The decision to continue or hold therapy is based on the ejection fraction as it relates to the institutions LLN and change in ejection fraction from screening (LVEF as measured at registration) according to the following table:</td></tr>
<tr><td>Relationship of LVEF to institution's LLN</td><td>Decrease < 10%</td><td>Decrease 10-15%</td><td>Decrease ≥ 16%</td></tr>
<tr><td>Normal</td><td>Continue</td><td>Continue</td><td>Continue and repeat MUGA/echo in 6 weeks</td></tr>
<tr><td>1-5% below LLN</td><td>Continue and repeat MUGA/echo in 6 weeks</td><td>Continue and repeat MUGA/echo in 6 weeks</td><td>Hold and repeat MUGA/echo in 6 weeks</td></tr>
<tr><td>≥ 6% below LLN</td><td>Continue and repeat MUGA/echo in 6 weeks</td><td>Hold and repeat MUGA/echo in 6 weeks</td><td>Hold and repeat MUGA/echo in 6 weeks</td></tr>
<tr><td colspan="4">Discontinue bevacizumab/placebo if:
• two consecutive “hold” categories occur
• three intermittent “hold” categories occur. (At the discretion of the investigator, bevacizumab/placebo may also be permanently discontinued prior to the occurrence of three intermittent “hold” categories.)
If LVEF is maintained at a “continue and repeat MUGA/echo” or improves from a “hold” to a “continue and repeat MUGA/echo” category, additional MUGA scans/echocardiogram prior to the next scheduled MUGA scan/echo will be at the discretion of the investigator.</td></tr>
<tr><td colspan="4">Symptomatic Cardiac Events</td></tr>
<tr><td colspan="4">Discontinue bevacizumab/placebo if:
o a patient has symptoms of CHF and a diagnosis of CHF is confirmed
o a patient has a myocardial infarction
Patients who discontinue bevacizumab/placebo due to cardiac toxicity may continue</td></tr>
</table>

paclitaxel at the investigator's discretion.	
**Proteinuria**	
UPC < 3.5	No change
UPC ≥ 3.5	Hold until UPC < 3.5. If treatment is delayed > 2 months due to proteinuria, bevacizumab/placebo should be permanently discontinued.
Nephrotic syndrome	Bevacizumab/placebo should be permanently discontinued
**Hypertension**	
Hypertension should be treated with anti-hypertensive medications as per standard practice.	
Controlled HTN	Continue therapy.
Uncontrolled or symptomatic HTN	Hold bevacizumab/placebo and add/adjust anti-hypertensives. If treatment is delayed > 4 weeks, due to uncontrolled hypertension, bevacizumab/placebo should be permanently discontinued.
Grade 4	Bevacizumab/placebo should be permanently discontinued.
**Hemorrhage/Bleeding**	
Grade 3	Bevacizumab/placebo should be discontinued in patients who are receiving full-dose anticoagulation.<br>For all other patients, hold bevacizumab/placebo until all of the following criteria are met:<br>• The bleeding has resolved and hemoglobin is stable.<br>• There is no bleeding diathesis that would increase the risk of therapy.<br>• There is no anatomic or pathologic condition that significantly increases the risk of hemorrhage recurrence.<br>Bevacizumab/placebo should be discontinued in patients who experience a repeat Grade 3 hemorrhagic event.
Grade 4	Bevacizumab/placebo should be permanently discontinued.
**Venous Thrombosis**	
Grade 3 or asymptomatic Grade 4	Hold bevacizumab/placebo treatment. If the planned duration of full-dose anticoagulation is < 2weeks, bevacizumab/placebo should be held until the full-dose anticoagulation period is complete. If the planned duration of full-dose anticoagulation is > 2 weeks, bevacizumab/placebo may be resumed during the period of full-dose anticoagulation if all of the following criteria are met:<br>• The patient must have an in-range INR (usually between 2 and 3) on a stable dose of warfarin (or other anticoagulant) prior to restarting bevacizumab/placebo treatment.<br>• The patient must not have pathological conditions that carry high risk of bleeding<br>• The patient must not have had Grade 3 hemorrhagic events while on study
Symptomatic Grade 4 or recurrent/worsening thromboembolic events after	Bevacizumab/placebo should be permanently discontinued.

resumption of bevacizumab/placebo	
**Arterial Thrombosis**	
Bevacizumab/placebo should be permanently discontinued in any patient who develops an arterial thromboembolic event (angina, myocardial infarction, transient ischemic attack, stroke or other arterial thromboembolic event)	
**Hepatic**	
Grade 0 - 2	No change
≥ Grade 3	Interrupt until ≤ Grade 1, then resume previous dose. If ≥ Grade 3 LFT elevations recur, bevacizumab/placebo should be permanently discontinued.
**Bowel Perforation/Anastomotic Dehiscence**	
Bevacizumab/placebo should be permanently discontinued in any patient who develops a bowl perforation or anastomotic dehiscence.	
**Anaphylaxis/Hypersensitivity**	
Mild (e.g., mild flushing, rash, pruritis)	No treatment needed. Supervise at bedside and complete bevacizumab/placebo infusion.
Moderate (e.g., moderate flushing, rash, mild dyspnea, chest discomfort)	Stop bevacizumab/placebo. Administer diphenhydramine 25 mg and dexamethasone 10 mg IV. After recovery, resume infusion at half the previous rate for 15 minutes. If no further symptoms occur, complete the infusion at the full dose rate. If moderate symptoms recur, bevacizumab/placebo should be permanently discontinued.
Severe (e.g., hypotension requiring pressors, angioedema, respiratory distress requiring bronchodilators)	Permanently discontinue bevacizumab/placebo. Administer diphenhydramine 25 mg and dexamethasone 10 mg IV. Add epinephrine or bronchodilators as needed.
**Other**	
Grade 0 - 2	No change
≥ Grade 3	Hold until resolved to ≤ Grade 1, resume at previous dose. If Grade 3 or greater toxicity recurs, bevacizumab/placebo should be permanently discontinued.
Bevacizumab (BV)/placebo should be held in patients with symptoms/signs suggestive of RPLS, pending work-up and management, including control of blood pressure. Bevacizumab/placebo should be discontinued upon diagnosis of RPLS.	

5.6 Supportive Care

- It is anticipated that nausea and vomiting may be a significant side effect of the treatment regimen. Therefore, the following combination regimen may be used: Dexamethasone, 10 mg IV or PO, plus a 5-HT3 receptor antagonist prior to AC chemotherapy. Prochlorperazine or another antiemetic at the physician’s discretion may be used before paclitaxel treatment.

- No cardioprotectant drugs may be used.
- All supportive measures consistent with optimal patient care will be given throughout the study.
- Pegfilgrastim or filgrastim are required after each AC treatment (cycle 1-4) for patients receiving dose dense AC (see section 5.2). Otherwise, hematologic growth factors are not required but may be used in accordance with the ASCO guidelines.

5.7 Duration of Therapy

Patients who have not recurred or developed a second primary (other than squamous or basal cell carcinoma of the skin, carcinoma *in situ* of the cervix, or lobular carcinoma *in situ* of the breast) may continue treatment according to the schema unless excessive toxicity occurs.

Patients will receive protocol treatment unless:

- Disease recurs. (If disease recurs locally or distantly, protocol treatment should be discontinued.)
- A second primary develops (including contralateral breast cancer but not including squamous or basal cell carcinoma of the skin, in situ of the cervix, or lobular carcinoma in situ of the breast).
- Extraordinary Medical Circumstances: If, at any time, the constraints of this protocol are detrimental to the patient's health, protocol treatment should be discontinued. In this event, submit forms according to the instructions in the E5103 Forms Packet.
- Patient withdraws consent.

5.8 Duration of Follow-up

For this protocol, all patients must also be followed through completion of all protocol therapy. All patients, including those who discontinue protocol therapy early, will be followed for recurrence and survival for 15 years from randomization. Patients who develop local recurrence will continue follow-up for distant recurrence. Patients who develop treatment related toxicities will continue follow-up until the toxicity resolves or is deemed irreversible.

6. Measurement of Effect

6.1 Local, Regional Recurrence

The development of a local or regional recurrence of breast cancer.

6.2 Distant Recurrence

The development of a distant recurrence of breast cancer.

6.3 Invasive Disease-Free Survival

Date of randomization to the date of first treatment failure (invasive ipsilateral, local/regional invasive or distant recurrence, invasive contralateral breast cancer, invasive non-breast second primary or death from any cause, whichever occurs first). Cases with incomplete follow-up, without documented IDFS event including those who develop squamous or basal cell skin cancers or in situ carcinomas of any site as their only event will be censored at the date of last disease evaluation (55).

Rev. 7/09

6.4 Survival

Date of randomization to date of death.

Rev. 3/13

6.5 Evidence of Recurrence

Recurrence must be documented by biopsy and/or evidence of disease on radiologic studies. Abnormal blood studies alone (e.g., elevated transaminases or alkaline phosphatase) are not sufficient evidence of relapse. Whenever possible, histologic proof of recurrence and estrogen and progesterone receptor data should be obtained.

Rev. 10/09, 7/10

6.6 Quality of Life Measurement

NOTE: The accrual goal for the QOL assessment has been met and this component has closed.

The quality of life and decision making correlative will be performed by the Dana Farber Cancer Center. Consecutive patients consenting to participate in E5103 will be asked to participate in this longitudinal decision-making and quality of life survey component of the clinical trial. At baseline, the institutional clinic research coordinator will hand patients a copy of the questionnaires to complete either in clinic or at home. This will be followed by a call from the centralized telephone research interviewer (based at Dana-Farber Cancer Institute) who will then interview the patients, collecting their answers over the phone. For all follow-up assessments, patients will be mailed a copy of the questionnaires 7-10 days prior to their scheduled interview, with a request to complete them prior to the arranged time of their interview. Patients who have not filled out the questionnaires can still be easily interviewed over the phone. The telephone interview can also overcome any literacy problems, as patients are read the questions. Those patients who are hard of hearing or deaf will be mailed the questionnaires with a stamped self-addressed envelope to be sent to the research team after they have completed the questionnaires. In clinic and telephone reminders will be utilized to enhance response rates.

Patients will be interviewed by telephone at baseline, 6 weeks (mid-treatment), 18 or 22 weeks (when treatment is unblinded for the AC q 2weeks or AC q 3 weeks patients, respectively), 12 months (completion of all treatment for all patients) and 18 months. The telephone interview will take approximately 30 minutes. The telephone interview is a well-established and validated method for obtaining quality of life information and has been demonstrated to be equivalent to data obtained through face-to-face interviews. The use of a centralized telephone assessment method has been established as a feasible, valid method for quality of life research, improving the quantity and quality of psychosocial data [Kornblith and Holland, 1996]. Studies since 1969 have demonstrated the equivalence of telephone interviews to in-person interviews (56, 62, 68, 70, 73, 92, 94, 66, 86, 97). The telephone interview has been successfully used in a cooperative clinical trials group setting in studies of cancer survivors (67, 78, 65), women with advanced breast cancer during treatment [Kornblith et al., 1993], in a study of social support models in breast cancer patients (76), patients with myelodysplastic syndrome treated with 5-azacytidine (77), prostate cancer patients with advanced stage disease [Kornblith et al., 2001], and patients participating in an intervention in which patient's distress was monitored and referred for treatment if reporting significant distress (79).

6.6.1 Measures

1) Overall Quality of Life: Functional Assessment of Cancer Therapy Scale – Breast Cancer [FACT-B + 4]

The FACT-B (57) includes the FACT-G [version 4.0], developed by Cella and colleagues as an overall cancer-specific quality of life questionnaire, with a 9 item subscale concerning breast cancer specific issues. The FACT-G consists of a 27 item 'core' quality of life measure grouped into four subscales: physical well-being, social/family well-being, emotional well-being, and functional well-being. Almost all items are rated on a 5 item Likert scale, from 0, 'not at all' to 4, 'very much'. The FACT-G has been tested on 545 patients with mixed cancer diagnoses. The internal consistency of the subscales ranges from acceptable to excellent, from .65-.82, with excellent internal consistency of the total score, with an alpha coefficient of .89. Test-retest reliability is also excellent within a 7 day period, with correlations ranging from .82-.92. Convergent validity has been demonstrated, with the FACT-G correlating significantly with other quality of life measures (FLIC, r=.79), and related constructs of psychological distress (e.g. Brief POMS, r=-.68) and the ECOG performance rating (PSR) (r=-.52) (59). The FACT-G has been able to distinguish between patients with metastatic and non-metastatic disease. The difference of 5 points on the FACT-G has been determined to be a minimally important difference.(MID) (58). The Breast cancer module consists primarily of physical symptoms, body image and sexual issues. The internal consistency of the FACT-B total score is excellent (alpha coefficient = .90) as well as the test-retest reliability (3-7 days) (r = .89). The FACT-B was demonstrated to be sensitive to changes in the ECOG Performance Status measure. Further evidence of validity was obtained by comparing groups differing in the ECOG Performance Status rating and disease stage,

with all subscales found to be significantly worse with worse performance status and more advanced disease, respectively. Four additional items were added to assess the impact of arm morbidity on patients following surgery. In terms of validity, lymphedema patients reported significantly greater arm problems than a matched sample of pre-operative trial participants (63). The FACT-B + 4 demonstrated sensitivity to change over time. Arm problems significantly increased from the pre-operative assessment and post-operative assessment at 1 month. (63). The FACT-G and FACT-B + 4 are frequently used in many clinical trials and studies of cancer patients' quality of life. It takes approximately 10 minutes to complete.

2) Physical and Emotional Symptoms: Memorial Symptom Assessment Scale (MSAS), Modified

The Memorial Symptom Assessment Scale (MSAS) (88) is designed to provide a comprehensive assessment of physical and psychological symptomatology experienced by cancer patients, in terms of three dimensions: severity, frequency and distress. The MSAS consists of 32 items, grouped into three subscales: physical symptoms occurring with greater frequency, physical symptoms occurring with less frequency and psychological symptoms. Twenty four of the 32 symptoms are rated in terms of three dimensions: frequency, severity, and distress, with frequency and severity rated on a 4 point Likert scale, from 1-4, and distress, on a 5 point scale, from 0-4. Eight items are rated only in terms of severity and distress, because frequency was not considered an appropriate dimension for certain symptoms (e.g. hair loss). The MSAS was validated on a sample of 246 ovarian, colon, breast and prostate cancer patients, and further tested in a study of 151 ovarian cancer patients (81). Internal consistency of the subscales was excellent with alpha coefficients of .84 and .88, respectively. All three dimensions were significantly interrelated: severity and frequency (r=.80); severity and distress (r=.70); frequency and distress (r= .43). The distress and frequency dimensions were the most predictive of other quality of life measures. In terms of validity, highly significant correlations were found between the MSAS and measures of psychological distress (MHI Psychological Distress subscale: r= .65), overall quality of life (FLIC : r= -.78), and Karnofsky Performance Status Scale (r= - .58). The Physical and Psychological subscales were found to differentially correlate with related measures, evidence of discriminant validity (87). Further, MSAS subscale and total scores were significantly worse in ovarian patients with Karnofsky scores of 80 or less vs. 90 or greater (81), and the MSAS Physical subscale significantly predicted survival in a mixed cancer population [Chang et al., 1998]. A 12 item brief version of the MSAS has been tested in 479 cancer patients and found to significantly correlate with overall quality of life (61). In order to assess symptoms specific to the treatment regimens in this trial, the following symptoms have been added to the MSAS, under the 'Other Symptoms' category**:** headache, chest pain, muscle or joint pain, abdominal pain, sore throat, heartburn, and frequent minor bleeding (e.g. nosebleeds). It takes approximately 10 minutes to complete.

3) Quality-Adjusted Life Years: EuroQol: EQ-5D

EQ-5D (89) is a measure of health status for use in evaluating health and healthcare. It provides a simple descriptive profile of 5 functional dimensions and generates a single index value for heath status on which full health is assigned a value of 1 and death a value of 0. Thus, the index can be used to obtain a utility for these dimensions for use in economic analyses. The EQ-5D has been specially designed to complement other quality of life measures such as the SF-36, or cancer-specific measures. Descriptively, the EQ-5D consists of 5 dimensions, mobility, self-care, usual activity, pain/discomfort, and anxiety/depression. Each dimension has 3 levels designated simply as ‘no problem’, ‘some problem’, or ‘extreme problem’, with patients checking the level most descriptive of their current level of function on each dimension. Five dimensions, each with three levels, yield 243 possible distinct health states comprising the classification system. The classification system has been assigned different standardized scores derived through U.S. population-based samples of respondents who assign values to subsets of the 243 states using the anchoring labels noted above (91). EQ-5D is designed for self-completion by patients and has been used extensively in mailed surveys. Quality-adjusted life years can be calculated from using the EQ-5D. It is cognitively simple, taking no more than a few minutes to complete.

4) Fear of Recurrence Scale

The Fear of Recurrence Scale developed by Kornblith, consists of 5 items, measuring a patient's beliefs and anxieties concerning their disease recurring, relevant to cancer survivor populations, as well as those with early stage disease, and patients who are being followed after treatment completion. All items are rated on a 5 item Likert scale, from 'strongly agree’ to ‘strongly disagree’. The Fear of Recurrence Scale has been used in four cancer survivor studies involving childhood survivors who had participated in a randomized trial of either prophylactic cranial radiation vs. systemic chemotherapy (CALGB 7611) (69), adult leukemia survivors (CALGB 8963) (67), endometrial and breast cancer survivors (82), and ovarian cancer survivors [Matulonis et al., 2008]. The Fear of Recurrence Scale meets the basic criterion of internal consistency: the alpha coefficient was .69 (for standardized variables) for childhood leukemia survivors (N=110), approximately 14 years post-treatment, .73 in the adult survivor study, (N=203) (67) .78 for the breast and endometrial cancer survivor study (82), and .83 for the ovarian cancer survivor study [Matulonis, unpublished data, 2007]. Evidence of convergent validity was provided by the Fear of Recurrence Scale significantly correlating with relevant emotional states: the Depression (r=.24, p=.01) and Paranoid Ideation subscales (r=.19, p< .05) of the Brief Symptom Inventory [BSI], and the Derogatis Body Image subscale (r=.31, p< .001) from the childhood leukemia survivors study; and the Psychosocial Adjustment to Illness total score (r=.33, p< .001), the BSI total score (.36, p< .001), and Body Image subscale (r=.30, p<

.001), in the adult leukemia survivor study. Further evidence of validity was found in Mehta et al.'s [2003] study in which general health perceptions correlated significantly with fear of recurrence: the higher the ratings of their health, the less fear of recurrence was reported ($p < .001$). It takes approximately 1-2 minutes to complete.

5) Sociodemographic Characteristics

Sociodemographic characteristics will be assessed using a modified version of the CALGB Background Information Form (71). It consists of 9 items querying patients about their age, race, Hispanic origin, marital, educational and employment status, income, and household composition. It takes approximately a minute to complete.

6) Decision Making and Risk/Benefit Evaluation

As part of the baseline assessment, we will ask patients about their perceptions of the magnitude of their cancer risk and of the likelihood that treatments will cause a serious problem (96, 98). We will also ask about their perceptions of how informed they are and their confidence in the treatment decision (99). We will ask patients to reevaluate their perceptions after they have been unblinded to the treatment arm, and again at 18 months after they have completed treatment. At the 18 months assessment, we will also survey patients to determine their willingness to accept the toxicities of bevacizumab, and the minimal acceptable benefit required to make treatment worthwhile. To do this we will use a modified version of the threshold technique that has been used in early stage breast cancer and other conditions (90, 74). The health state descriptions will be accompanied by visual graphics depicting likelihood of the benefits and toxicities of bevacizumab (100, 101). The descriptions will be reviewed by two medical oncologists for accuracy and then piloted with 10 breast cancer survivors to ensure patient comprehension. The resulting distribution of benefit to toxicity as determined by patients will be used to estimate the potential value of introducing a biomarker, in terms of the percentage of patients for whom it would likely change treatment decisions.

Timing of Measures

	**Measure**	**Timepoints**				
		**Baseline**	**6 weeks**	**18 or 22 weeks (post-unblinding)***	**12 months****	**18 months**
	**FACT-B + 4**	**X**	**X**	**X**	**X**	**X**
	**MSAS, modified**	**X**	**X**	**X**	**X**	**X**
	**EQ-5D**	**X**	**X**	**X**	**X**	**X**
	**Fear of Recurrence Scale**	**X**	**X**	**X**	**X**	**X**
Rev. 1/11	**Sociodemographics**	**X**		**X (modified)**	**X (modified)**	**X (modified)**
	**Decision-Making Survey**	**X**		**X**		**X (modified)**

*When treatment is unblinded for the AC every 2 weeks or AC every 3 weeks.

**Completion of treatment for all patients.

7. Study Parameters

7.1 Therapeutic Parameters

1. Prestudy (pre-Step 1) CBC (with differential and platelet count) should be done ≤ 8 weeks prior to randomization.
2. All required prestudy chemistries, as outlined in Section 3, should be done ≤ 8 weeks prior to randomization.
3. Required prestudy cardiac evaluation (MUGA or ECHO), as outlined in Section 3, should be done ≤ 8 weeks prior to randomization.

NOTE: When recording prestudy results on the Case Report Form, please make sure that ALL relevant dates are clearly given. Do NOT put all the results under the date for Day 1 of protocol treatment unless they were actually done that day. *Record the actual dates.*

NOTE: See section 4.9 for MUGA/echocardiogram, ECG and Urine Protein Studies reimbursement information.

7.1.1 STEP 1: ALL TREATMENT GROUPS (Arms A, B, C): Cycle 1-8

			AC + B/P		T + B/P			**Follow-up** (Arms A and B)
		Baseline[9]	C1[1] & C3 (day 1)	C2 & C4 (day 1)	C5 (day 1)	C6, C7 & C8 (day 1)	C5-C8 (days 8 & 15)	
Rev. 7/09	**Physical[11]**							
Rev. 10/09 7/10	H & P	X	X	X	X	X		Q3 mos. for 0-2 yrs., Q6 mos. for 2-5 yrs., and annually from 5-15 yrs.
	ECOG PS	X	X	X	X	X		
	Weight	X	X	X	X	X		
	Height	X						
	**Laboratory[11]**							
	Hematology (WBC, ANC, Hgb, Plts)	X	X	X	X	X	X	X[10]
	Chemistry (Cr, T. bili, AST, Na)	X	X	X	X	X		X[10]
	Coagulation (PT/INR, PTT)[2]	X			X			
Rev. 7/09	Urine protein:creatinine ratio[3]	X	C3 only		X	C7 only		
	Serum or Urine HCG[4]	X						
	**Imaging**							
	Chest x-ray[5]	X						
	Bone scan[6]	X						
	Mammogram							Annually to 15 years
	**Cardiac[7,12]**							
	ECG	X			X			Within 2 weeks of completing therapy and 1 year from registration
Rev. 7/09, 9/10	MUGA or Echocardiogram[13]	X			X			
Rev. 9/10	Cardiac symptoms assessment[15]							At 2 years (24 months) post-registration
Rev. 7/09	**Treatment (+/- 2 days)**							
	Doxorubicin		X	X				
	Cyclophosphamide		X	X				
	Pegfilgrastim or Filgrastim[8]		X	X				
	Paclitaxel				X	X	X	
	Bevacizumab/Placebo		X	X	X	X		
	Unblind treatment assignment					C8 only		
	**Sample Submissions**	X	See section 7.2 or section 10					
Rev. 10/09, 9/10	QOL Assessment[14]	QOL to be administered at baseline and 6 weeks, 18 or 22 weeks, 12 months, and 18 months post-registration.						

1. Pre-study evaluations performed within 7 days of Day 1 do not need to be repeated.
2. Additional monitoring required for patients who require full dose anticoagulation. See sections 5.5.3 and 8.1.13 for details
3. See Appendix V for procedure for obtaining urine protein:creatinine ratio
4. Required in women of child-bearing potential only, within 7 days prior to randomization.
5. Chest CT can be substituted at investigator's discretion.
6. If clinically indicated.
7. An echocardiogram (2-D only) to measure ejection fraction (LVEF) or MUGA are adequate for this study. Use same method for each evaluation at the same radiology facility where the baseline was done whenever possible.
8. Pegfilgrastim or filgrastim required only in patients receiving dose dense every 2 week AC. See section 5.2 for details.
9. (Rev. 7/09) Baseline scans, x-rays, ECG, blood pressure, and MUGA or echocardiogram must be performed within ≤ 8 weeks prior to randomization.
10. Hematology and chemistry labs should be collected 3 months after completing protocol therapy.
11. (Rev. 7/09) Physical and laboratory tests may be done up to 72 hours prior to the scheduled day of treatment.
12. ECG and MUGA/echocardiogram may be done up to 14 days prior to the scheduled day of treatment.
13. (Rev. 7/09) In the event that you are notified of a technetium-99 supply shortage, follow the instructions below.
    1. For patients being worked up for E5103:
        - If a MUGA scan is the preferred method to determine left ventricular ejection fraction (LVEF) then every effort should be made to determine the availability of technetium-99 at your institution.
        - If your institution does not have enough Technetium-99 ECHOs should be used as the preferred assessment for new patients to avoid future changes (i.e. during follow up) in the assessment method.
        - When ECHO is used for baseline scanning, an ECHO must be performed at baseline and every subsequent time-point listed in section 7 of the protocol.
        - It is imperative that a patient does NOT switch from an ECHO to a MUGA during the trial.
        - And for added consistency in interpretation, it is recommended that the ECHOs are conducted at the same facility and read by the same cardiologist if possible.
    2. For patients already registered to E5103 (who have already had a baseline MUGA scan):
        - ECOG-ACRIN will allow a +/- 14 day window to obtain the MUGA scans at each of the required time-points. If a follow-up MUGA cannot be obtained with this variance in the time requirement, an ECHO should be substituted.
        - If the ECHO indicates a significant decline in cardiac function, you must contact Kathy Miller, MD, Study Chair, at (317) 274-1690 or kathmill@iupui.edu for further instruction on how to proceed.
        - When the shortage is resolved, these patients can resume assessments using MUGA scans or continue using ECHO scans. This decision is up to the discretion of the Institution.

    Please note that: (1) the provisions for the substitution of ECHOs for MUGAs, and (2) the extended window of time to obtain required MUGAs apply only during the technetium shortage.
14. (Rev. 10/09, 7/10, 9/10) QOL assessment should be completed on the first 500 patients entered onto E5103 who receive protocol treatment at: baseline (prior to starting treatment) and at 6 weeks, 18 or 22 weeks, 12 months and 18 months post-registration.

    NOTE: The accrual goal for the QOL assessment has been met and this component has closed
15. (Rev. 9/10) MUGAs, ECHOs, or EKGs done as a result of the 24-month post-registration cardiac assessment are not eligible for reimbursement.

7.1.2 STEP 2: Arm D only: Bevacizumab Monotherapy (Cycles 9-18) (open label)

		Bevacizumab										**Follow-up** (Arm C/Arm D)
		C9 (day 1)	C10 (day 1)	C11 (day 1)	C12 (day 1)	C13 (day 1)	C14 (day 1)	C15 (day 1)	C16 (day 1)	C17 (day 1)	C18 (day 1)	
Rev. 7/09	**Physical[5]**											
Rev. 10/09	H & P	X			X			X			X	Q3 mos. for 0-2 yrs., Q6 mos. for 2-5 yrs., and annually from 5-15 yrs.
	ECOG PS	X			X			X			X	
	Weight	X	X	X	X	X	X	X	X	X	X	
	**Laboratory[5]**											
	Hematology (WBC, ANC, Hgb, Plts)	X	X	X	X	X	X	X	X	X	X	X[4]
	Chemistry (Cr, T. bili, AST, Na)	X	X	X	X	X	X	X	X	X	X	X[4]
	Coagulation (PT/INR, PTT)[1]	X										
Rev. 7/09	Urine protein:creatinine ratio[2]	X			X			X			X	
	**Imaging**											
	Mammogram											Annually to 15 years
	**Cardiac[3,6]**											
	ECG	X						X				Within 2 weeks of completing therapy[7]
Rev. 7/09	MUGA or Echocardiogram[8]	X						X				
Rev. 9/10	Cardiac symptoms assessment[10]											At 2 years (24 months) post-registration)
	**Treatment (+/- 2 days)**											
	Bevacizumab	X	X	X	X	X	X	X	X	X	X	
Rev. 10/09	QOL Assessment[9]	QOL to be administered at baseline and 6 weeks, 18 or 22 weeks, 12 months, and 18 months post-registration.										

1. Additional monitoring required for patients who require full dose anticoagulation. See sections 5.5.3 and 8.1.13 for details
2. See Appendix V for procedure for obtaining urine protein:creatinine ratio
3. An echocardiogram (2-D only) to measure ejection fraction (LVEF) or MUGA are adequate for this study. Use same method for each evaluation at the same radiology facility where the baseline was done whenever possible.
4. Hematology and chemistry labs should be collected 3 months after completing protocol therapy.

Rev. 7/09

5. Physical and laboratory tests may be done up to 72 hours prior to the scheduled day of treatment
6. ECG and MUGA/echocardiogram may be done up to 14 days prior to the scheduled day of treatment.

Rev. 9/10

7. Arm C patients who do not re-register to Arm D or who do not begin treatment to Arm D should have follow-up cardiac exams within 2 weeks of completing therapy and 12 months post-registration. Arm D patients who do not complete Step 2 should have a follow-up cardiac exam at 12 months post-registration.

Rev. 7/09

8. In the event that you are notified of a technetium-99 supply shortage, follow the instructions below.
   1. For patients being worked up for E5103:
      - If a MUGA scan is the preferred method to determine left ventricular ejection fraction (LVEF) then every effort should be made to determine the availability of technetium-99 at your institution.
      - If your institution does not have enough Technetium-99 ECHOs should be used as the preferred assessment for new patients to avoid future changes (i.e. during follow up) in the assessment method.
      - When ECHO is used for baseline scanning, an ECHO must be performed at baseline and every subsequent time-point listed in section 7 of the protocol.
      - It is imperative that a patient does NOT switch from an ECHO to a MUGA during the trial.
      - And for added consistency in interpretation, it is recommended that the ECHOs are conducted at the same facility and read by the same cardiologist if possible.
   2. For patients already registered to E5103 (who have already had a baseline MUGA scan):
      - ECOG-ACRIN will allow a +/- 14 day window to obtain the MUGA scans at each of the required time-points. If a follow-up MUGA cannot be obtained with this variance in the time requirement, an ECHO should be substituted.
      - If the ECHO indicates a significant decline in cardiac function, you must contact Kathy Miller, MD, Study Chair, at (317) 274-1690 or kathmill@iupui.edu for further instruction on how to proceed.
      - When the shortage is resolved, these patients can resume assessments using MUGA scans or continue using ECHO scans. This decision is up to the discretion of the Institution.

   Please note that: (1) the provisions for the substitution of ECHOs for MUGAs, and (2) the extended window of time to obtain required MUGAs apply only during the technetium shortage.

Rev. 10/09, 7/10

9. QOL assessment should be completed on the first 500 patients entered onto E5103 who receive protocol treatment at: baseline (prior to starting treatment) and at 6 weeks, 18 or 22 weeks, 12 months and 18 months post-registration.

   NOTE: The accrual goal for the QOL assessment has been met and this component has closed.

Rev. 9/10

10. MUGAs, ECHOs, or EKGs done as a result of the 24-month post-registration cardiac assessment are not eligible for reimbursement.

Rev. Add14 7.2 Biological Sample Submissions

Rev. 10/09 Samples are being requested for banking for embedded correlative and future studies and should be submitted only from patients who have given written

Rev. 12/07 consent for the use of their samples for these purposes. Samples should be submitted as outlined in section 10.

NOTE: ECOG-ACRIN requires all samples submitted to be entered and tracked via the online ECOG-ACRIN Sample Tracking System (STS). See section 10.4.

7.2.1 Baseline Samples

Rev. 10/09 Please submit all samples for patients consenting to banking. A portion of the plasma sample will be used for the correlative study added in Addendum #4. if the plasma sample is unavailable, tumor blocks may be used.

- Rev. 7/10 One (1) block of primary tumor
- Rev. 7/10 One (1) block of lymph node positive tumor
- Rev. 12/07 Serum from one (1) 6-10 mL tubes, no anticoagulant (e.g. red top or SST tube)
- Rev. 12/07 Plasma and cells from two (2) 5 mL EDTA tubes

Rev. 1/15 **NOTE:** If blocks are unavailable contact the ECOG-ACRIN CBPF at (844) 744-2420 to obtain alternative sample submission guidelines.

Rev. 2/13
Rev. Add14

7.2.2 Requirements for Ancillary Study EL112LAB

For all patients who have registered to Step 3, forms and samples are to be submitted as outlined in Appendix XV. A schedule summary is provided below.

Kits for sample collection and shipment are available for sites in the United Stated and Canada. To order kits, follow the instructions in Appendix XIV.

Questions regarding kits can be directed to projectmanagement@cenetron.com or call the Cenetron clinical trials group at 512-439-2000.

Institutions outside the United States and Canada who wish participate in this ancillary study are to contact the ECOG-ACRIN CBPF to discuss the logistics of specimen submissions.

NOTE: ECOG-ACRIN requires all samples submitted to be entered and tracked via the online ECOG-ACRIN Sample Tracking System (STS). See section 10.4.

	**Registration to Step 3**	**At 1, 2, 3, 4, & 5 years After Step 3 Registration**	**Tumor Recurrence**
Health Questionnaires	X	X	
Fasting Blood Samples[2]	X	X	X
Tumor Sample	X[1]		X

1. Primary tumor tissue (FFPE), if not submitted previously, must be submitted within 4 weeks following registration to Step 3. If previously submitted materials are inadequate for the purposes of this ancillary study, additional materials will be requested. Questions may be directed to ecog-acrin.tst@jimmy.harvard.edu. Rev. 1/15
2. Fasting draws are strongly encouraged. Provide time of last caloric intake in STS.

8. Drug Formulation and Procurement

Rev. 12/11
Rev. 1/15

8.1 Bevacizumab (NSC 704865, IND 113919)

All investigators who receive a copy of the protocol should also obtain a copy of the Investigator's Brochure (IB). IB's are available from the Pharmaceutical Management Branch, CTEP, DCTD, NCI and may be obtained by emailing the IB Coordinator (ibcoordinator@mail.nih.gov) or by calling the IB Coordinator at 240-276-6575.

The bevacizumab to be supplied for this protocol is intended for clinical trial use only and is not the commercially available Avastin®. Investigational bevacizumab and commercially available Avastin® may be produced at separate facilities and some differences may exist between the two products, although both are required to meet similar product testing criteria and are expected to be very similar in safety and activity. For further details and molecule characterization, see the updated bevacizumab Investigator Brochure.

Clinical Supplies: Bevacizumab (NSC 704865) and matching placebo will be provided free of charge by Genentech and distributed by the Pharmaceutical Management Branch (PMB), Cancer Therapy Evaluation Program (CTEP), Division of Cancer Treatment and Diagnosis (DCTD), National Cancer Institute (NCI).

Rev. 7/10

Bevacizumab and matching placebo will be supplied in 4 mL fill glass vials each containing 100 mg (Bevacizumab) or 0 mg (Placebo for Bevacizumab) of bevacizumab. In the future, only 400 mg vials of bevacizumab or placebo for 400 mg bevacizumab will be available. At that time, bevacizumab and matching placebo will be supplied in 16 mL fill glass vials each containing 400 mg (bevacizumab) or 0 mg (placebo for bevacizumab) of bevacizumab. The blinded, patient-specific vials will be sealed in a cardboard box with a tamper-evident seal.

For **Cycles 1 through 8 (CLASSICAL or DOSE DENSE / Arms A, B, and C)**, each box of bevacizumab / placebo will be labeled with

- The protocol number (i.e., "E5103")
- The box number (e.g., "Box 1 of 3", "Box 2 of 3", and "Box 3 of 3")
- The number of vials (e.g., "60 vials")
- The patient ID number (e.g., "99999", where "99999" represents the unique patient ID number assigned by ECOG-ACRIN at the time of patient registration)
- The patient initials (i.e., first initial, last initial [e.g., "FL"])

Rev. 7/10

- The agent identification (i.e., "Bevacizumab 100 mg or Placebo" or "Bevacizumab 400 mg or Placebo")
- A blank line for the pharmacist to enter the patient's name
- Storage instructions (i.e., "Store in refrigerator [2 – 8°C]. Do not freeze. Do not shake.")
- Emergency contact instructions
- A Julian date

For **Cycles 9 through 18 (CLASSICAL or DOSE DENSE / Arm D ONLY),** each box of open-label bevacizumab will be labeled with

- The protocol number (i.e., "E5103")
- The box number (e.g., "Box 1 of 3", "Box 2 of 3", and "Box 3 of 3")
- The number of vials (e.g., "75 vials")
- The patient ID number (e.g., "99999", where "99999" represents the unique patient ID number assigned by ECOG-ACRIN at the time of patient registration)
- The patient initials (i.e., first initial, last initial [e.g., "FL"])
- Rev. 7/10 The agent identification (i.e., "Bevacizumab 100 mg" or "Bevacizumab 400 mg")
- A blank line for the pharmacist to enter the patient's name
- Storage instructions (i.e., "Store in refrigerator [2 – 8°C]. Do not freeze. Do not shake.")
- Emergency contact instructions
- A Julian date

The Julian date indicates the day the box was labeled and shipped and is composed of the last two digits of the calendar year (e.g., 2006 = 06, 2007 = 07) and a day count (e.g., January 1 = 001, December 31 = 365). For example, a box labeled and shipped on January 1, 2006 would have a Julian date of '06001' and a box labeled and shipped on December 31, 2007 would have a Julian date of '07365'. The Julian date will be used by PMB for recalls. When a lot expires, PMB will determine the last date the expired lot was shipped and will recall all vials (i.e., both Bevacizumab and Placebo) shipped on or before that date thus eliminating any chance of breaking the blind.

Rev. 1/15 **Questions about drug orders, transfers, returns, or accountability should be addressed to the PMB by calling 240-276-6575 Monday through Friday between 8:30am and 4:30pm Eastern Time.**

Drug Ordering:

E5103 Shipment Schedule

**Patient Randomized with ECOG-ACRIN**	**Initial e-Order Transmitted by ECOG-ACRIN**	**Initial e-Order Received and Approved by PMB**	**Initial Order Shipped By PMB**	**Initial Order Received at Site ***
Monday	Monday	Tuesday	Wednesday	Thursday
Tuesday	Tuesday	Wednesday	Thursday	Friday
Wednesday	Wednesday	Thursday	Monday	Tuesday
Thursday	Thursday	Friday	Monday	Tuesday
Friday	Friday	Monday	Tuesday	Wednesday

***arrival time approximate / shipments sent by Federal Express**

CLASSICAL Arms A, B, and C (Cycles 1 through 8)

No blinded starter supplies will be available for this phase. Blinded, patient-specific clinical supplies will be sent to the registering investigator at the time of randomization. This randomization will be performed by the ECOG-ACRIN Operations Office - Boston in Boston, MA. The patient ID number assigned by the ECOG-ACRIN Operations Office - Boston must be recorded by the registering institution for proper study medication dispersion. Once a patient has been registered with the ECOG-ACRIN Operations Office - Boston, the ECOG-ACRIN Operations Office - Boston will electronically transmit a clinical drug request for that patient to the PMB. This request will be entered and transmitted by the ECOG-ACRIN Operations Office - Boston the day the patient is registered and will be processed by the PMB the next business day and shipped the following business day. All shipments will be sent on blue ice by FedEx (generally one to two day delivery). Thus, if a patient is registered on Monday, ECOG-ACRIN would enter a clinical drug request for that patient on Monday and PMB would process the request on Tuesday and ship the drug on Wednesday. Both United States and Canadian sites could expect to receive their order either Thursday or Friday. Note that PMB will only send blue ice shipments on Monday through Thursday for delivery on Tuesday through Friday. Thus, if a patient is registered on Wednesday, the order will be processed on Thursday and shipped the following Monday for delivery on Tuesday or Wednesday. The initial request will be for a sufficient number of vials to complete **4 cycles** (i.e., **a 12 week supply at 15mg/kg IV every three weeks**) based on the patient's weight in "kg" provided by ECOG-ACRIN at the time of patient registration. When cycle 4 is administered (i.e., three weeks before cycle 5 is needed), sites may reorder an additional **4 cycles** (i.e., **a 12 week supply at 15mg/kg IV every three weeks**) by completing an NCI Clinical Drug Request form and faxing it to the PMB at
Rev. 1/15 240-276-7893. The NCI Clinical Drug Request form is available on the CTEP home page (http://ctep.cancer.gov) or by calling the PMB at 240-276-6575. The protocol number (i.e., E5103), the assigned patient ID number (e.g., "99999"), the patient initials (e.g., "FL"), the number of vials remaining from the initial shipment, and the patient's weight (in "kg") should be entered on each order. All drug orders will be shipped directly to the physician registering the patient.

NOTE: At the completion of cycle 8, ALL remaining clinical supplies of bevacizumab / placebo should be returned to PMB (see "Drug Returns" below).

DOSE DENSE Arms A, B, and C (Cycles 1 through 8)

No blinded starter supplies will be available for this phase. Blinded, patient-specific clinical supplies will be sent to the registering investigator at the time of randomization. This randomization will be performed by the ECOG-ACRIN Operations Office - Boston in Boston, MA. The patient ID number assigned by the ECOG-ACRIN Operations Office - Boston must be recorded by the registering institution for proper study medication dispersion. Once a patient has been registered with the ECOG-ACRIN Operations Office - Boston, the ECOG-ACRIN Operations Office - Boston will electronically transmit a clinical drug request for that patient to the PMB. This request will be entered and transmitted by the ECOG-ACRIN Operations Office - Boston the day the patient is registered and will be processed by the PMB the next business day and shipped the

following business day. All shipments will be sent on blue ice by FedEx (generally one to two day delivery). Thus, if a patient is registered on Monday, ECOG-ACRIN would enter a clinical drug request for that patient on Monday and PMB would process the request on Tuesday and ship the drug on Wednesday. Both United States and Canadian sites could expect to receive their order either Thursday or Friday. Note that PMB will only send blue ice shipments on Monday through Thursday for delivery on Tuesday through Friday. Thus, if a patient is registered on Wednesday, the order will be processed on Thursday and shipped the following Monday for delivery on Tuesday or Wednesday. The initial request will be for a sufficient number of vials to complete **4 cycles** (i.e., **an 8 week supply at 10mg/kg IV every two weeks**) based on the patient's weight in "kg" provided by ECOG-ACRIN at the time of patient registration. When cycle 4 is administered (i.e., two weeks before cycle 5 is needed), sites may reorder an additional **4 cycles** (i.e., **a 12 week supply at 15mg/kg IV every three weeks**) by completing an NCI Clinical Drug Request form and faxing it to the PMB at
Rev. 1/15
240-276-7893. The NCI Clinical Drug Request form is available on the CTEP home page (http://ctep.cancer.gov) or by calling the PMB at 240-276-6575. The protocol number (i.e., E5103), the assigned patient ID number (e.g., "99999"), the patient initials (e.g., "FL"), the number of vials remaining from the initial shipment, and the patient's weight (in "kg") should be entered on each order. All drug orders will be shipped directly to the physician registering the patient.

NOTE: At the completion of cycle 8, ALL remaining clinical supplies of bevacizumab / placebo should be returned to PMB (see "Drug Returns" below).

CLASSICAL or DOSE DENSE / Arm D ONLY (Cycles 9 through 18)

Rev. 5/08
Rev. 7/09

In order to obtain open – label clinical supplies, patients must be unblinded with the ECOG-ACRIN Operations Office - Boston at the completion of cycle 8 / day 1 (see section 4.6) and must be re-registered to Arm D (see section 4.7). Registration to Step 2 Arm D should occur during Cycle 8 to allow time for drug ordering and shipping so patients receive their next dose of bevacizumab as scheduled on Cycle 9 Day 1. Step 2 Arm D treatment must begin no later than 2 months following the last dose of Step 1 bevacizumab.

Rev. 10/09

NOTE: For patients registering to Step 2, the PMB will recalculate the drug doses shipped based on the weight reported during the cycle in which the patient was unblinded (not based on the weight reported at baseline).

No open label starter supplies will be available for this phase. Once the patient has been registered to Step 2, Arm D, the ECOG-ACRIN Operations Office - Boston will electronically transmit a clinical drug request to the PMB for open-label patient specific supplies. **The patient ID number will NOT change.** This request will be entered and transmitted by the ECOG-ACRIN Operations Office - Boston the day the patient is registered to step 2 and will be processed by the PMB the next business day and shipped the following business day. All shipments will be sent on blue ice by FedEx (generally one to two day delivery). Thus, if a patient is registered to step 2 on Monday, ECOG-ACRIN would enter a clinical drug request for that patient on Monday and PMB would process the request on Tuesday and ship the drug on Wednesday. Both United States and

Canadian sites could expect to receive their order either Thursday or Friday. Note that PMB will only send blue ice shipments on Monday through Thursday for delivery on Tuesday through Friday. Thus, if a patient is registered to step 2 on Wednesday, the order will be processed on Thursday and shipped the following Monday for delivery on Tuesday or Wednesday. The initial request will be for a sufficient number of vials to complete **5 cycles** (i.e., **a 15 week supply at 15mg/kg IV every three weeks**) based on the patient's weight in "kg" provided by ECOG-ACRIN at the time the patient is unblinded. When cycle 13 is administered (i.e., three weeks before cycle 14 is needed), sites may reorder an additional **5 cycles** (i.e., **a 15 week supply at 15mg/kg IV every three weeks**) by completing an NCI Clinical Drug Request form and faxing it to the PMB at
Rev. 1/15 240-276-7893. The NCI Clinical Drug Request form is available on the CTEP home page (http://ctep.cancer.gov) or by calling the PMB at 240-276-6575. The protocol number (i.e., E5103), the assigned patient ID number (e.g., "99999"), the patient initials (e.g., "FL"), the number of vials remaining from the previous shipment, and the patient's weight (in "kg") should be entered on each order. All drug orders will be shipped directly to the physician registering the patient.

NOTE: At the completion of cycle 18, ALL remaining clinical supplies of bevacizumab should be returned to PMB (see "Drug Returns" below).

8.1.1 Drug Transfers

Vials **MAY NOT** be transferred from one patient to another patient or from one protocol to another protocol. All other transfers (e.g., a patient moves from one participating clinical site to another participating clinical site, the responsible investigator at a given clinical site changes) must be approved **in advance** by the PMB. To obtain an approval for transfer, investigators should complete and
Rev. 1/15 submit to the PMB (fax number 240-276-7893) a Transfer Investigational Agent Form available on the CTEP home page (http://ctep.cancer.gov) or by calling the PMB at 240-276-6575. The patient ID number (e.g., "99999") and the patient initials (e.g., "FL") should be entered in the "Received on NCI Protocol No." and the "Transferred to NCI Protocol No." fields in addition to the protocol number (i.e., "E5103").

8.1.2 Drug Returns

Only unopened clinical supplies should be returned to the PMB. When it is necessary to return study drug (e.g., sealed vials remaining when a patient completes cycle 8, sealed vials remaining when a patient completes cycle 18, sealed vials remaining when a patient permanently discontinues protocol treatment, expired vials recalled by the PMB), investigators should return the study drug to the PMB using the NCI Return Drug List available on the CTEP home page
Rev. 1/15 (http://ctep.cancer.gov) or by calling the PMB at 240-276-6575. The patient ID number (e.g., "99999") and the patient initials (e.g., "FL") should be entered in the "Lot Number" field. A separate line item is required for each patient ID (e.g., "99999") and for each agent (e.g., "bevacizumab / placebo", open label "bevacizumab") being returned.

Opened vials should be destroyed on site in accordance with institutional policy.

8.1.3 Drug Accountability

Rev. 7/09
Rev. 1/15

The investigator, or a responsible party designated by the investigator, must maintain a careful record of the receipt, disposition, and return of all drugs received from the PMB using the NCI Investigational Agent Accountability Record available on the CTEP home page (http://ctep.cancer.gov) or by calling the PMB at 240-276-6575.

Rev. 7/09

For Arms A,B, and C, a patient-specific DARF (Drug Accountability Record Form) should be kept for each cycles 1-8 (blinded phase). In addition, a separate patient-specific DARF should be kept for each case on Arm D Cycle 9-18 (open label).

8.1.4 Emergency Unblinding

Rev. 5/08

NOTE: The information provided below is for the use by a physician, nurse, CRA or pharmacist treating the paitent. These contact numbers should not be used by patients. Patients should be instructed to call their doctor's office in the event of an emergency or adverse event that may result in the need to unblind the patient.

In the event of an emergency or severe adverse reaction necessitating identification of the medication for the welfare of the patient, please contact the Study Chair, Dr. Kathy Miller at kathmill@iupui.edu (317-274-1690) first to ensure the reason for unblinding is valid. Then contact a member of the ECOG-ACRIN Operations Office - Boston Drug Team at 900.drugorder@jimmy.harvard.edu (617-632-3610) Monday through Friday between 9:00am and 5:00pm Eastern Time. For unblinding outside of these hours, contact AnswerConnect at 1-866-296-8940. This service will request the reason for unblinding and then page the on-call ECOG-ACRIN staff who will return your call and provide the unblinded treatment assignment if applicable. **Remember, AnswerConnect should only be contacted outside of normal business hours and only in the event of an emergency.** The ECOG-ACRIN Operations Office - Boston or AnswerConnect will require the protocol number (i.e., "E5103"), the patient ID number (e.g., "99999"), and the patient initials (e.g., "FL") to unblind the patient. Please note that, if a patient is emergently unblinded, he/she is considered to be off-therapy and must discontinue protocol treatment. Be sure to include patient's ID number and initials in all emails.

8.1.5 Description

Bevacizumab is a recombinant humanized anti-VEGF monoclonal antibody, consisting of 93% human and 7% murine amino acid sequences. The agent is composed of human IgG framework and murine antigen-binding complementarity-determining regions.

Bevacizumab blocks the binding of vascular endothelial growth factor (VEGF) to its receptors resulting in inhibition of angiogenesis.

Rev. 7/10

8.1.6 How Supplied

"Bevacizumab" and "placebo" are supplied as a clear to slightly opalescent, sterile liquid ready for parenteral administration. For "bevacizumab," each 100mg (25mg/mL – 4mL fill) and 400 mg (25 mg/mL – 16 mL) glass vial contains bevacizumab with phosphate, trehalose, polysorbate 20, and Sterile Water for Injection, USP. For "placebo," each 0mg (0mg/mL – 4mL fill or 16 mL fill) glass vial contains phosphate, trehalose, polysorbate 20, and Sterile Water for Injection, USP. **DO NOT USE COMMERCIAL BEVACIZUMAB OR AVASTIN**.

The bevacizumab to be supplied for this protocol is intended for clinical trial use only and is not the commercially available Avastin®. Investigational bevacizumab and commercially available Avastin® may be produced at separate facilities and some differences may exist between the two products, although both are required to meet similar product testing criteria and are expected to be very similar in safety and activity. For further details and molecule characterization, see the updated bevacizumab Investigator Brochure.

8.1.7 Storage and Stability

Bevacizumab is shipped on blue ice for next day delivery. On receipt, bevacizumab should be stored in the refrigerator (2º to 8ºC) and should remain refrigerated until just prior to use. Do not freeze. Do not shake. Shelf-life studies of bevacizumab are continuing. Investigators will be notified when lots have expired. The sterile single use vials contain no antibacterial preservatives; therefore, vials should be discarded eight hours after initial entry.

8.1.8 Preparation

Rev. 5/08

Vials contain no preservative and are intended for single use only. Place the calculated dose of bevacizumab in 100 mL of 0.9% sodium chloride for injection. Once diluted in 0.9% sodium chloride for injection, the bevacizumab solution must be administered within 8 hours.

8.1.9 Administration

Bevacizumab is administered intravenously as a continuous infusion. The initial dose should be administered over a minimum of 90 minutes. If no adverse reactions occur after the initial dose, the second dose should be administered over a minimum of 60 minutes. If no adverse reactions occur after the second dose, all subsequent doses should be administered over a minimum of 30 minutes. If infusion-related adverse reactions occur, all subsequent infusions should be administered over the shortest period that was well tolerated.

To ensure complete delivery of bevacizumab, the IV infusion line must be flushed with 0.9% Sodium Chloride for Injection. Please note that

this flush is not included in the infusion times. The following are two recommended methods for flushing the bevacizumab IV infusion line:

- When the bevacizumab infusion is complete, add an additional 50mL of 0.9% Sodium Chloride for Injection to the bevacizumab infusion bag. Continue the infusion until a volume equal to that of the volume contained in the tubing has been administered.
- Replace the empty bevacizumab infusion bag with a 50mL bag of 0.9% Sodium Chloride for Injection and infuse a volume equal to the volume contained in the tubing.

8.1.10 Kinetics

Estimated half-life of Bevacizumab is approximately 20 days (range 11-50 days).

The clearance of bevacizumab was higher in males and in patients with a higher tumor burden.

8.1.11 Drug Interactions

Bevacizumab may increase the concentration of SN38 (the active metabolite of irinotecan) by as much as 33%. This may potentially increase the incidence of irinotecan-induced side effects such as diarrhea and leucopenia.

8.1.12 Adverse Effects

Allergy/Immunology:	Allergic reaction/hypersensitivity. Infusion-related reactions.
Blood/Bone Marrow:	Leukopenia, neutropenia, thrombocytopenia.
Cardiac:	Hypertension/hypertensive crisis, cardiac ischemia/infarction, supraventricular arrhythmia, left ventricular dysfunction (congestive heart failure), hypotension, syncope.
Constitutional symptoms:	Asthenia, fever, rigors/chills, weight loss.
Dermatology/skin:	Exfoliative dermatitis, complications with wound healing, rash, skin ulceration, urticaria
Gastrointestinal:	GI perforation and wound dehiscence, sometimes complicated by intra-abdominal abscesses. Large bowel leakage, GI fistula, intestinal obstruction, intestinal necrosis, mesenteric venous occlusion, colitis, mucositis/stomatitis, nausea, vomiting, anorexia, constipation, diarrhea,

	heartburn/dyspepsia, dry mouth, taste disturbance.
Hemorrhage/Bleeding:	Life-threatening or fatal pulmonary hemorrhage (primarily in lung cancer patients), CNS bleeding, GI hemorrhage, subarachnoid hemorrhage, hemorrhagic stroke, epistaxis (nose bleeds), vaginal bleeding, gum bleeding.
Infection:	Infection with normal ANC.
Metabolic/Laboratory:	Increased: alkaline phosphatase, ALT (SGPT), AST (SGOT), Bilirubin, serum creatinine. Hyponatremia and hypokalemia.
Neurology:	Cerebrovascular ischemia, RPLS or PRES, dizziness, abnormal gait, confusion.
Ocular:	Excessive lacrimation.
Pain:	Abdominal pain, chest/thoracic pain, headache, arthralgias, myalgias, generalized.
Pulmonary/ Upper Respiratory:	Dyspnea, cough, bronchospasm/wheezing, voice changes (hoarseness).
Renal/Genitourinary:	Proteinuria, nephrotic syndrome.
Vascular:	Life-threatening and potentially fatal arterial thromboembolic events: cerebral infarction, transient ischemic attacks, myocardial infarction, angina. Venous thromboembolic events: deep vein thrombosis, intra-abdominal thrombosis.

8.1.13 Patient Care Information

Monitor CBC and platelets. For patients taking warfarin for thrombosis, monitor PT or INR closely (weekly until two stable therapeutic levels attained). For patients on warfarin for venous access prophylaxis, routine PT monitoring.

Monitor patient closely during infusion, for infusion related events and for bleeding.

Monitor blood pressure prior to each dose to assess for development of hypertension.

Instruct patient to monitor and report signs/symptoms of: bleeding (nose bleeds, blood in sputum), wound healing problems, abdominal pain, thromboembolic problems (chest or leg pain, dyspnea, vision changes, severe headache, cough, swelling)

Baseline urine protein:creatinine ratio (UPC) must be performed and repeated every 2-3 bevacizumab doses. If UPC ≥ 3.5, see Section 5.5.3.

Therapy should be suspended several weeks before elective surgery and should not restart until surgical incision is fully healed.

Treat pain, arthralgias, etc. with acetaminophen, or other pain relief strategies that do not interfere with the clotting cascade.

Interval between last major surgery and day 1 of treatment must be > 28 days. (Non-operative biopsy or placement of a vascular device is not considered major surgery).

8.1.14 References:

Bevacizumab (Avastin™) Full Prescribing Information. Genentech, Inc. December 2004.

Bevacizumab Investigators Brochure, Genentech, December 2003.

8.1.15 Date/Reviewer:

Updated February, 2005/ Helen McFarland, PharmD (410)-502-1036.

8.2 Doxorubicin

NOTE: Please refer to the commercial package insert for more information.

8.2.1 Other Names

Adriamycin, Rubex, Adriamycin RDF, Adriamycin PFS, hydroxydaunorubicin, hydroxydaunomycin, ADR

8.2.2 Classification

Anthracycline antibiotic

8.2.3 Mode of Action

Intercalation between adjoining nucleotide pairs in the DNA helix causes inhibition of DNA and DNA-dependent RNA synthesis. Free radical generation is responsible for cardiac toxicity. Doxorubicin also inhibits topoisomerase II.

8.2.4 Storage and Stability

Rubex or Adriamycin RDF intact vials are stable protected from light at room temperature. Adriamycin PFS vials must be refrigerated. Reconstituted solutions are stable for 24 hours at room temperature and 48 hours under refrigeration. The Adriamycin RDF 150 mg multidose vial is stable after reconstitution for 7 days at room temperature or 15 days if refrigerated and protected from sunlight.

8.2.5 Dose Specifics

60 mg/m² for the first 4 cycles of treatment only.

8.2.6 Preparation

Add 5, 10, 25, 50, or 75 mL of preservative-free normal saline to the 10, 20, 50, 100, or 150 mg vial to produce a solution containing 2 mg/mL.

8.2.7 Administration

Intravenously, as a bolus injection (IV pushing through running IV of NS).

8.2.8 Incompatibilities

Physically incompatible with heparin, fluorouracil, aminophylline, cephalothin, dexamethasone, diazepam, hydrocortisone, and furosemide.

8.2.9 Compatibilities

Stable with vincristine in normal saline for 5 days at room temperature protected from light. Also compatible in solution with cyclophosphamide.

8.2.10 Availability

Commercially available as powder for injection in 10, 20, 50, 100, 150 mg vials, and as 2 mg/mL solution for injection in 10, 20, 50, and 200 mg vials.

8.2.11 Side Effects

Hematologic:	Leukopenia (dose-limiting), also thrombocytopenia and anemia.
Dermatologic:	Alopecia, usually complete; hyperpigmentation of nailbeds and dermal creases; radiation recall; facial flush.
Gastrointestinal:	Nausea and vomiting, sometimes severe; anorexia, diarrhea; mucositis.
Cardiovascular:	Arrhythmias, ECG changes; rarely sudden death. Congestive heart failure due to cardiomyopathy related to total cumulative dose; risk is greater with doses greater than 550 mg/m², mediastinal irradiation, pre-existing cardiac disease, advanced age; risk is reduced with weekly or continuous infusion regimens.
Other:	Red discoloration of urine; fever; anaphylactoid reaction; may enhance cyclophosphamide cystitis or mercaptopurine hepatotoxicity;
Local effects:	Vesicant if extravasated; flush along vein.

8.2.12 Nursing/Patient Implications

Monitor CBC, platelet counts.

Vesicant - do not extravasate. Refer to extravasation protocol if inadvertent infiltration occurs.

Advise patient of alopecia. Instruct on how to obtain wig, hairpiece, etc. Hair loss generally occurs 2-4 weeks after injection and is usually complete.

Advise patient of red discoloration of urine for 24 hours after administration of the drug.

Administer antiemetics as indicated.

Assess for stomatitis and treat symptomatically. Generally occurs 7-10 days after injection.

Be aware of "Adria" flare -most common reaction consists of an erythematous streak up the vein. It is associated with urticaria and pruritus. Occasionally the use of corticosteroids and/or antihistamines has been useful.

Monitor for signs and symptoms of cardiomyopathy. Calculate total cumulative dose with each administration.

8.2.13 References

Speth PA. Clinical pharmacokinetics of doxorubicin. Clin Pharmacokinetics 1988; 15:51-31.

Von Hoff DD, *et al.* Risk factors for doxorubicin-induced congestive heart failure. Ann Intern Med 1979; 91:710-717.

Lum BL, *et al.* Doxorubicin: Alteration of dose scheduling as a means of reducing cardiotoxicity. Drug Intell Clin Pharm 1985; 19:259-264.

8.3 Cyclophosphamide

NOTE: Please refer to the commercial package insert for more information.

8.3.1 Other Names

Cytoxan, Neosar, CTX, CPM

8.3.2 Classification

Cyclophosphamide is a prodrug biotransformed to active alkylating metabolites by a mixed function microsomal oxidase system.

8.3.3 Mode of Action

Cyclophosphamide metabolites are thought to disrupt cell division primarily by cross-linking DNA strands. Cyclophosphamide is considered cell cycle phase non-specific.

8.3.4 Storage and Stability

Tablets and injectable powder are stored at room temperature 25°C (77°F). The temperature is not to exceed 30°C (90 F°). Reconstituted parenteral solutions are stable for 24 hours at room temperature for 6-14 days if refrigerated.

8.3.5 Dose Specifics

600 mg/m² for the first 4 cycles of treatment only.

8.3.6 Preparation

Dissolve the 100 mg, 200 mg, 500 mg, 1 g, and 2 g vials in 5, 10, 25, 50, and 100 mL of sterile water, respectively, resulting in a solution of 20 mg/mL. Shake vials vigorously and warm slightly in lukewarm water to facilitate dissolution. The lyophilized form is more easily solubilized.

Reconstituituted solutions may be further diluted in D5W, D5W/NS, D5W/Ringer's Injection, Lactated Ringer's Injection, and ½ NS.

8.3.7 Administration

IV infusion, in 250 mL NS over 20-30 minutes.

8.3.8 Compatibilities

Numerous compatibility studies have been published. For specific details refer to handbook on injectable drugs by Lawrence A. Trissel.

8.3.9 Availability

Cyclophosphamide is commercially available for parenteral injection as 100 mg,
200 mg, 500 mg, 1 g, and 2 g vials.

8.3.10 Side Effects

Side effects vary significantly based on the specific dose and duration of cyclophosphamide.

8.3.10.1 Incidence More Frequent (>5%)

Anemia, leukopenia (usually asymptomatic; less frequently fever and/or chills)

Thrombocytopenia (usually asymptomatic; less frequently unusual bleeding or bruising; black tarry stools; blood in urine or stools; pinpoint red spots on skin). Nadir counts usually occur 7 to 12 days after administration and recovery usually compete by day 17 to 21.

Alopecia

Anorexia, nausea and vomiting

Gonadal suppression (azoospermia, missed menstrual periods) resulting in infertility. Return of normal gonadal function and fertility occurs with time in many younger men and women.

Hemorrhagic cystitis

8.3.10.2 Incidence Less Frequent (1-5%)

Stomatitis

8.3.10.3 Incidence Rare (1%)

Anaphylaxis (tachycardia, shortness of breath, wheezing, tightness in throat)

Flushing or redness of face

Diarrhea

Skin rash

Pneumonitis or interstitial pulmonary fibrosis\

Syndrome of inappropriate antidiuretic hormone (siadh)

Chemical phlebitis (redness, swelling or pain at site of injection)

Secondary malignancies

Blurred vision, cardiac toxicity presenting as congestive heart failure

Hemorrhagic mycodarditis

Cardiac necrosis

Pericarditis (seen with high dose regimens used with bone marrow transplantation)

8.3.11 Drug Interactions

8.3.11.1 Digoxin

Several studies conducted in lymphoma patients receiving combination chemotherapy including cyclophosphamide revealed a 20–50% reduction in digoxin absorption when digoxin tablets were administered. When digoxin capsules were administered no significant decrease in digoxin absorption occurred. To avoid decreased serum digoxin levels the use of digoxin in liquid form (liquid or capsules containing liquid digoxin) instead of tablets is recommended.

8.3.11.2 Pentostatin

Two case reports describe fatal cardiac toxicity in patients receiving CTX 6.4 g/m² over 4 days and pentostatin 4 mg/m² over 4 hours on day 3. Until additional data from clinical trials demonstrate the safety of concurrent use of these drugs concurrent administration is not recommended.

8.3.11.3 Succinylcholine

Cyclophosphamide may prolong the effects of succinylcholine by irreversibly inhibiting the enzyme pseudocholinesterase. Limited clinical observations and *in vitro* studies suggest that prolonged apnea might result when succinylcholine is administered to some patients also receiving cyclophosphamide. Management options include avoiding concurrent therapy or if concurrent therapy can not be avoided, to monitor for prolonged succinylcholine effect in patients receiving both drugs. If cyclophosphamide has been administered within 10 days

of succinylcholine, extreme caution should be used after succinylcholine administration. The anesthesiologist should be informed of the potential for succinylcholine-induced apnea and appropriate precautions and monitoring should be implemented.

8.3.11.4 Trastuzumab

In early clinical trials the concurrent administration of cyclophosphamide and trastuzumab increased the incidence and severity of cardiac dysfunction. Until additional data from clinical trials demonstrate the safety of concurrent use of these drugs concurrent administration is not recommended.

8.3.12 Nursing Implications

Monitor CBC, platelet count. Advise patients of increased risk of infection with absolute neutrophil count less than 500 cells/mm³ and increased risk of bleeding with platelet counts less than 20,000 cells/ mm³. Advise patients to call the clinic if they develop a fever above 101°F or notice any easy bruising, petechiae (pinpoint red spots on skin), or prolonged bleeding.

Advise patient of possible alopecia. Instruct how to obtain wig, hairpiece, etc.

Assess hydration and fluid balance. Patients receiving larger doses should force fluids up to 2 liters above normal intake for 72 hours after administration. Instruct patients to void more frequently to minimize occurrence of hemorrhagic cystitis.

Premedicate with antiemetics.

Observe for possible phlebitis at injection site.

Administer antiemetics as indicated.

8.3.13 References

American Hospital Formulary Service 99 – Drug Information; 832-837.

Cytoxan Package Insert, Princeton, NJ: Mead Johnson Oncology Products 1998; July Micromedex Inc. Vol. 101; 1999.

USPDI Volume 1 1999; 1128-1134.

Trissel L.A, Handbook on injectable drugs (8th Ed), Bethesda, MD: American Society of Hospital Pharmacists, 1994, Pp. 287-295.

Cazin B, Gorin NC, Laporte JP. Cardiac complications after bone marrow transplantation: A report of a series of 63 consecutive transplants. Cancer 1986; 57:2061-2069.

Gryn *et. al.* Pentostatin increases the acute toxicity of high dose cyclophosphamide. Bone Marrow Transplantation 1993; 12:217-220.

Pedersen-Bjergaard J, Ersboll J, Sorenson HM. Risk of acute nonlymphocytic leukemia and preleukemia in patients treated with

cyclophosphamide for Non-Hodgkin's lymphoma. Ann Intern Med 1985; 103:195-200.

Stillwell TJ, Benson RJ. Cyclophosphamide-induced hemorrhagic cyctitis: A review of 100 patients. Cancer 1988; 61:451-457.

Zucchero FJ, Ed. Evaluation of drug interactions. St. Louis: Professional Drug Systems, 1997; 2/35, 12/21.

Hansten Pd, Ed. Drug interactions analysis and management. Applied Therapeutics, Inc., Vancouver, WA 1998; 185-186.

Trastuzumab Package Insert, South San Francisco, CA Genentech, Inc. 1998; September.

8.4 Paclitaxel

NOTE: Please refer to the commercial package insert for more information.

8.4.1 Other Names

Taxol, NSC 673089.

8.4.2 Classification

Antimicrotubule agent.

8.4.3 Mode of Action

Promotes microtubule assembly and stabilizes tubulin polymers by preventing their depolarization, resulting in the formation of extremely stable and nonfunctional microtubules, and consequently inhibition of many cell functions.

8.4.4 Storage and Stability

The intact vials are stored under refrigeration. Freezing does not adversely affect the product. Solutions diluted to a concentration of 0.3 to 1.2 mg/mL in normal saline, 5% dextrose, 5% dextrose and normal saline, or 5% dextrose in Ringer's solution are stable for up to 27 hours when stored at room temperature and normal room light.

8.4.5 Dose Specifics

80 mg/m^2

8.4.6 Preparation

The concentrated solution must be diluted prior to use in normal saline, 5% dextrose, 5% dextrose and normal saline, or 5% dextrose in Ringer's solution to a concentration of 0.3 -1.2 mg/mL. Solutions exhibit a slight haze, common to all products containing non-ionic surfactants. Glass, polypropylene, or polyolefin containers and non-PVC-containing (nitroglycerin) infusion sets should be used. A small number of fibers (within acceptable limits established by the USP) have been observed after dilution. Therefore, a hydrophilic 0.22 micron in-line filter should be used. Analyses of solutions filtered through IVEX-2 and IVEX-HP (Abbott) 0.2 micron filters showed no appreciable loss of potency.

Solutions exhibiting excessive particulate formation should not be used.

8.4.7 Administration

Administered as an intravenous infusion over 1 hour.

8.4.8 Incompatibilities

Avoid the use of PVC bags and infusion sets due to leaching of DEHP (plasticizer). Ketoconazole may inhibit paclitaxel metabolism, based on *in vitro* data.

8.4.9 Availability

A concentrated solution of 6 mg/mL in polyoxyethylated castor oil (Cremophor EL) 50% and dehydrated alcohol 50% is commercially available in 5 mL vials.

8.4.10 Side Effects

Hematologic: Myelosuppression (neutropenia, leukopenia, thrombocytopenia, anemia).

Hypersensitivity: Thought to be caused by the Cremophor vehicle. Minor symptoms include hypotension, flushing, chest pain, abdominal or extremity pain, skin reactions, pruritus, dyspnea, and tachycardia. More severe reactions include hypotension requiring treatment, dyspnea with bronchospasm, generalized urticaria, and angioedema. The majority (53%) of the reported reactions occurred within 2-3 minutes of initiation of treatment and 78% occurred within the first 10 minutes. Reactions usually occurred with the first and second doses.

Cardiovascular: Atrial arrhythmia (sinus bradycardia [usually transient and asymptomatic], sinus tachycardia, and premature beats); significant events include syncope, hypotension, other rhythm abnormalities (including ventricular tachycardia, bigeminy, and complete heart block requiring pacemaker placement), and myocardial infarction. Hypertension (possibly related to concomitant medication -- Dexamethasone) may also occur.

Neurologic: Sensory (taste changes); peripheral neuropathy; arthralgia and myalgia (dose-related, more common when colony-stimulating factors are also administered); seizures; mood alterations; neuroencephalopathy; hepatic encephalopathy; motor neuropathy; and autonomic neuropathy (paralytic ileus and symptomatic hypotension).

Dermatologic: Alopecia (universal, complete and often sudden, between days 14-21); injection site reactions (erythema, induration, tenderness, skin

	discoloration); infiltration (phlebitis, cellulitis, ulceration, and necrosis, rare); radiation recall; and rash.
Gastrointestinal:	Nausea, vomiting, diarrhea, stomatitis, mucositis, pharyngitis, typhlitis (neutropenic enterocolitis), ischemic colitis, and pancreatitis.
Hepatic:	Increased AST, ALT, bilirubin, alkaline phosphatase; hepatic failure, and hepatic necrosis.
Other:	Fatigue, headache, light-headedness, myopathy, elevated serum creatinine, elevated serum triglycerides, and visual abnormalities (sensation of flashing lights, blurred vision).

8.4.11 Nursing/Patient Implications

Monitor CBC and platelet count prior to drug administration.

Symptom management of expected nausea, vomiting, and stomatitis.

Monitor for and evaluate abdominal pain occurring after paclitaxel administration (especially in severely neutropenic patients and in those receiving G-CSF) due to the risk of ischemic and neutropenic enterocolitis.

Advise patients of possible hair loss.

Cardiac monitoring for assessment of arrhythmias in patients with serious conduction abnormalities.

Monitor liver function tests.

Advise patient of possible arthralgias and myalgias which may occur several days after treatment. Monitor for symptoms of peripheral neuropathy.

Monitor for signs and symptoms of hypersensitivity reactions. Insure that the recommended premedications have been given. Premedications (diphenhydramine, steroids, and H2 blocker) appear to reduce the incidence and severity of hypersensitivity reactions but do not provide complete protection. Emergency agents (diphenhydramine and epinephrine) should be available.

Evaluate IV site regularly for signs of infiltration. It is not known if paclitaxel is a vesicant; however, the CremophorEL vehicle for this drug can cause tissue damage.

In-line filtration with a 0.22 micron filter should be used.

8.4.12 References

Rowinsky EK, Casenave LA, Donehower RC. Taxol: A novel investigational microtubule agent. J Natl Cancer Inst 1990; 82:1247-1259.

Gregory RE, DeLisa AF. Paclitaxel: A new antineoplastic agent for refractory ovarian cancer. Clin Pharm 1993; 12: 401-415.

Rowinsky EK, Eisenhauer EA, Chaudry V, *et al.* Clinical toxicities encountered with paclitaxel. Semin Oncology 1993; 20:1-15.

Walker FE. Paclitaxel: Side effects and patient education issues. Semin Oncology Nurs 1993; 9(suppl 2):6-10.

8.5 Filgrastim

8.5.1 Other Names

G-CSF, Neupogen, recombinant-rnethionyl human granulocyte-colony stimulating factor, granulocyte colony-stimulating factor, r-metHuG-CSF.

8.5.2 Classification

Colony stimulating factor; cytokine.

8.5.3 Mode of Action

Hematopoietic regulator with effects on both immature bone marrow progenitors and mature myeloid cells; it acts by supporting growth of human bone marrow- derived colony forming units and enhancing neutrophil-mediated antibody dependent cellular toxicity.

8.5.4 Storage and Stability

Filgrastim should be refrigerated and not allowed to freeze. It is stable for 24 hours at room temperature if the solution remains clear. At a concentration of *5* mcg/mL or greater in D5W, filgrastim is stable for 7 days at room or refrigerator temperatures. At dilutions from 5 to 14 mcg/mL, albumin in a final concentration of 2 mcg/mL should be added to protect against adsorption. Addition of albumin is unnecessary when the drug is diluted to a concentration greater than or equal to 15 mcg/mL in D5W. Concentrations of less than 5 mcg/mL should not be used. Dilutions in D5W are stable in glass bottles, polyvinyl chloride, polyolefin, or polypropylene bags and IV sets, and Travenol Infusors.

Undiluted filgrastim injection is stable in BD tuberculin syringes for up to 24 hours at 15-30ºC or for up to 7 days when refrigerated at 2-8ºC.

8.5.5 Dose Specifics

5 mcg/kg once a day, days 2-11.

8.5.6 Preparation

Draw appropriate dose into syringe for subcutaneous injection. May be further diluted in DSW for continuous infusion. Albumin, if required, is added before filgrastim.

8.5.7 Route of Administration

Subcutaneously or intravenous bolus. Has also been given by intravenous or subcutaneous continuous infusion.

8.5.8 Incompatibilities

Normal saline.

8.5.9 Availability

Rev. 5/08

Commercially available as a 300 mcg/mL solution in 1 mL and 1.6 mL vials.

8.5.10 Side Effects

1. Musculoskeletal: Mild to moderate medullary bone pain in 20% to 25% of patients.
2. Dermatologic and hypersensitivity: Redness, swelling, itching, and pain may occur at the injection site. Transient, generalized rash has been reported occasionally. Anaphylactoid and allergic reactions have been reported rarely.
3. Hematologic: Leukocytosis occurs occasionally.
4. Other: Less frequently reported side effects include transient supraventricular arrhythmia, splenomegaly, and vasculitis. Transient increases in serum concentrations of uric acid, LDH, alkaline phosphatase and leucocyte alkaline phosphatase have been reported after cytotoxic chemotherapy.

8.5.11 Nursing Implications

1. Patients may need help to deal with financial concerns due to the expense of this drug.
2. Patients or care givers must be instructed and be able to demonstrate their ability to properly measure and administer the drug parenterally. Filgrastim should be kept in the refrigerator until needed and the vials should not be shaken.
3. Instruct patients to administer the drug at the same time each day. Vials of filgrastim are single-dose and remaining drug should be discarded.
4. Refer to protocol for information regarding requirements for patient documentation of doses administered, temperatures, side effects, etc.
5. Administration of filgrastim is usually started 24 hours after the end of chemotherapy. Refer to protocol for specific information.
6. Acetaminophen is the recommended analgesic for mild bone pain.
7. Duration of therapy will be determined by the return of blood counts (WBC/ANC) to specified values. Refer to protocol for specific information regarding duration of therapy.
8. Discuss with the patient proper methods of disposal of syringes, needles, vials, etc.

8.5.12 References

Takads M, *et al*. Recombinant human G-CSF (rGt-CSF) in patients with non-small cell lung cancer (NSCLC) treated with combination chemotherapy (CT) of mitomycin, vindesine and cisplatin (MVP). Proc Am Soc Clin Oncol 1990.; 9:224.

Crawford J, *et al.* G-CSF: Prevention of chemotherapy induced febrile neutropenia in patients with small cell lung cancer. Proc Am Soc Clin Oncol 1990; 9:229.

Sheridan W, Morstyn G. Phase II study of granulocyte colony-stimulating factor in autologous bone marrow transplantation. Proc Am Soc Clin Oncol 1989; 8:178.

Morstyn G, *et al*. Effect of granulocyte colony stimulating factor on neutropenia induced by cytotoxic chemotherapy. Lancet 1988; 1:667-72.

Neidhart J, *et al*. (Granulocyte colony-stimulating factor stimulates recovery of granulocytes in patients receiving dose-intensive chemotherapy without bone marrow transplantation. J Clin Oncol 1989; 7:1685-92.

Gabrilove JL, *et al.* Effect of granulocyte colony-stimulating factor on neutropenia and associated morbidity due to chemotherapy for transitional-cell carcinoma of the urothelium. N Engl J Med 1988; 318:1414-22.

Morstyn G, *et al*. Treatment of chemotherapy-induced neutropenia by subcutaneously administered granulocyte colony-stimulating factor with optimization of dose and duration of therapy, J Clin Oncol 1989; 7:1554-62.

Personal communication, Michael Pecsok, Pharm. D. Amgen, May 25, 1994.

8.6 Pegfilgrastim

8.6.1 Other Names

Peg G-CSF, Neulasta®.

8.6.2 Classification

Colony stimulating factor; cytokine.

8.6.3 Mode of Action

Hematopoietic regulator with effects on both immature bone marrow progenitors and mature myeloid cells; it acts by supporting growth of human bone marrow- derived colony forming units and enhancing neutrophil-mediated antibody dependent cellular toxicity. Pegfilgrastim is filgrastim that is covalently bound to monomethoxypropylene glycol to reduce renal clearance and extend the duration of action.

8.6.4 Storage and Stability

Filgrastim should be refrigerated and not allowed to freeze. It is stable for 48 hours at room temperature if the solution remains clear.

8.6.5 Dose Specifics

6 mg subcutaneously, day 2.

8.6.6 Preparation

Pegfilgrastim is available in pre-filled syringes.

8.6.7 Administration

Subcutaneous administration only.

8.6.8 Incompatibilities

None.

8.6.9 Availability

Pegfilgrastim is commercially available and is supplied as a preservative-free solution containing 6 mg (0.6 mL) of pegfilgrastim (10 mg/mL) in a single-dose syringe with a 27 gauge, 1/2 inch needle with an UltraSafe® Needle Guard. Pegfilgrastim is provided in a dispensing pack containing one syringe.

8.6.10 Side Effects

Musculoskeletal: Mild to moderate medullary bone pain in 20% to 25% of patients.

Dermatologic and hypersensitivity: Redness, swelling, itching, and pain may occur at the injection site. Transient, generalized rash has been reported occasionally. Anaphylactoid and allergic reactions have been reported rarely.

Hematologic: Leukocytosis occurs occasionally.

Other: Less frequently reported side effects include transient supraventricular arrhythmia, splenomegaly, and vasculitis. Transient increases in serum concentrations of uric acid, LDH, alkaline phosphatase and leucocyte alkaline phosphatase have been reported after cytotoxic chemotherapy.

8.6.11 Nursing Implications

Patients may need help to deal with financial concerns due to the expense of this drug.

Patients or caregivers must be instructed and be able to demonstrate their ability to properly administer the drug subcutaneously. Pegfilgrastim should be kept in the refrigerator until needed and the prefilled syringe should not be shaken. Syringes of pegfilgrastim are single-dose and remaining drug should be discarded.

Refer to protocol for information regarding requirements for patient documentation of dose administered, temperatures, side effects, etc.

Administration of pegfilgrastim is usually started 24 hours after the end of chemotherapy. Refer to protocol for specific information.

Acetaminophen is the recommended analgesic for mild bone pain.

Discuss with the patient proper methods of disposal of syringes.

Do not confuse Neupogen® (filgrastim) and Neulasta®. Do not use Neupogen® and Neulasta® concurrently.

Neulasta® has not been studied for use in acute leukemia chemotherapy treatment programs, high-dose chemotherapy for bone marrow transplant, or for peripheral blood stem cell mobilization.

8.6.12 References

Neulasta® package insert. Amgen; Thousand Oaks, CA1/31/02.

Holmes FA, O'Shaughnessy JA, Vukelja S, et al. Blinded randomized multicenter study to evaluate single administration pegfilgrastim once per cycle versus daily filgrastim as an adjunct to chemotherapy in patients with high-risk stage II or stage III/IV breast cancer. Journal of Clinical Oncology. 2002;20(3)727-31.

Johnston E, Crawford J, Blackwell S, et al. Randomized dose escalation study of SD/01 compared with daily filgrastim in patients receiving chemotherapy. Journal of Clinical Oncology. 2000;18(13)2522-8.

8.7 Epirubicin

NOTE: Please refer to the commercial package insert for more information.

8.7.1 Other Names

Ellence®

8.7.2 Classification

Anthracycline antibiotic

8.7.3 Mode of Action

Intercalation between adjoining nucleotide pairs in the DNA helix causes inhibition of

DNA and DNA-dependent RNA synthesis. Such intercalation triggers DNA cleavage by topoisomerase II, resulting in cytocidal activity. Epirubicin also inhibits DNA helicase activity, preventing the enzymatic separation of double-stranded DNA and interfering with replication and transcription. Free radical generation is responsible for cardiac toxicity.

8.7.4 Storage and Stability

8.7.4.1 Ready to use solution

Store unopened vials refrigerated between 2ºC and 8ºC (36ºF and 46ºF). Do not freeze. Protect from light. Storage of the solution for injection at refrigerated conditions can result in the formation of a gelled product. This gelled product will return to a slightly viscous to mobile solution after 2 to a maximum of 4 hours equilibration at controlled room temperature (15–25°C). Epirubicin should be used within 24 hours of first penetration of the rubber stopper. Discard any unused solution.

8.7.4.2 Lyophilized powder for solution

Store unopened vials at 25°C (77°F); excursions permitted to 15-30°C (59-86°F). Protect from light. Discard unused portion. Store Upright.

8.7.5 Dose Specifics

8.7.5.1 Classical AC Schedule (q21day cycles)

For patients receiving the classical AC regimen using a 21 day cycle, if doxorubicin is not available, epirubicin is administered at 90 mg/m² day 1 q21 days as a substitute for doxorubicin. If the dose of doxorubicin has been or is to be modified due to toxicity, the dose of epirubicin is reduced by the same percentage dose reduction as indicated for the doxorubicin using 90 mg/m^2 as the full calculated dose of epirubicin. (Ex. For a patient 1.5 m^2, a 20% dose reduction of doxorubicin would equal 72 mg (90 x 0.8). For the same patient, an equivalent 20% dose reduction for epirubicin would equal 108 mg (135 x 0.8).

8.7.5.2 Dose Dense AC Schedule (q14day cycle)

For patients receiving the dose dense AC regimen using a 14 day cycle, if doxorubicin is not available, epirubicin is administered at 90 mg/m² day 1 q14 days as a substitute for doxorubicin. If the dose of doxorubicin has been or is to be modified due to toxicity, the dose of epirubicin is reduced by the same percentage dose reduction indicated for the doxorubicin using 90 mg/m^2 as the full calculated dose of epirubicin. (Ex. For a patient 1.5 m^2, a 20% dose reduction of doxorubicin would equal 72 mg (90 x 0.8). For the same patient, a 20% dose reduction for epirubicin would equal 108 mg (135 x 0.8).

8.7.6 Preparation

8.7.6.1 Ready to use solution.

Epirubicin injection is available in polypropylene single-use vials containing 2 mg epirubicin hydrochloride per ml as a sterile, preservative-free, ready-to-use solution in the following strengths: 50 mg/25 ml single-use vial and 200 mg/100 ml single-use vial.

8.7.6.2 Lyophilized powder for solution.

Epirubicin injection, powder, lyophilized, for solution is also available in single-use vials containing 50 mg and 200 mg epirubicin hydrochloride.

Prior to use, epirubicin lyophilized powder for Injection 50 mg and 200 mg vials must be reconstituted with 25 ml and 100 ml, respectively, of Sterile Water for Injection, USP, resulting in a solution concentration of 2 mg/ml with a pH of 4.7 to 5.0. Shake vigorously. It may take up to 4 minutes

for epirubicin hydrochloride to completely dissolve. Reconstituted solutions are stable for 24 hours when stored at 2 to 8°C (36 to 46°F) and protected from light, or 25°C (77°F) in normal lighting conditions. Epirubicin for Injection can be further diluted with Sterile Water for Injection, USP

8.7.7 Administration

Epirubicin is administered by intravenous infusion. Venous sclerosis may result from an injection into a small vessel or from repeated injections into the same vein. Extravasation of epirubicin during the infusion may cause local pain, severe tissue lesions (vesication, severe cellulitis) and necrosis. It is recommended that epirubicin be slowly administered into the tubing of a freely running intravenous infusion. Patients receiving initial therapy at the recommended starting doses of 90 mg/m2 should generally have epirubicin infused over 15-20 minutes. If possible, veins over joints or in extremities with compromised venous or lymphatic drainage should be avoided. A burning or stinging sensation may be indicative of perivenous infiltration, and the infusion should be immediately terminated and restarted in another vein. Perivenous infiltration may occur without causing pain. Facial flushing, as well as local erythematous streaking along the vein, may be indicative of excessively rapid administration. It may precede local phlebitis or thrombophlebitis.

8.7.8 Incompatibilities

Prolonged contact of epirubicin with any solution of an alkaline pH should be avoided as it will result in hydrolysis of the drug. Epirubicin should not be mixed with heparin or fluorouracil due to chemical incompatibility that may lead to precipitation.

8.7.9 Compatibilities

Epirubicin can be used in combination with other antitumor agents, but it is not recommended that it be mixed with other drugs in the same syringe.

8.7.10 Availability

8.7.10.1 Ready to use solution (Ellence® Pfizer).

Epirubicin injection is commercially available in polypropylene single-use vials containing 2 mg epirubicin hydrochloride per ml as a sterile, preservative-free, ready-to-use solution in the following strengths: 50 mg/25 ml single-use vial and 200 mg/100 ml single-use vial.

8.7.10.2 Lyophilized powder for solution (Multiple generic suppliers).

Epirubicin injection, powder, lyophilized, for solution is also commercially available in single-use vials containing 50 mg and 200 mg epirubicin hydrochloride.

8.7.11 Side Effects

Hematologic: Leukopenia (dose-limiting), also thrombocytopenia and anemia. Secondary acute myelogenous leukemia (AML) has been reported in patients with breast cancer treated with anthracyclines, including epirubicin. The occurrence of refractory secondary leukemia is more common when such drugs are given in combination with DNA-damaging antineoplastic agents, when patients have been heavily pretreated with cytotoxic drugs, or when doses of anthracyclines have been escalated. The cumulative risk of developing treatment-related AML or myelodysplastic syndrome (MDS), in 7110 patients with breast cancer who received adjuvant treatment with epirubicin-containing regimens, was estimated as 0.27% at 3 years, 0.46% at 5 years and 0.55% at 8 years

Cardiovascular: Arrhythmias, ECG changes; rarely sudden death. Myocardial toxicity, manifested in its most severe form by potentially fatal congestive heart failure (CHF), may occur either during therapy with epirubicin or months to years after termination of therapy. The probability of developing clinically evident CHF is estimated at approximately 0.9% at a cumulative dose of 550 mg/m2, 1.6% at 700 mg/m2, and 3.3% at 900 mg/m2. In the adjuvant treatment of breast cancer, the maximum cumulative dose used in clinical trials was 720 mg/m2. The risk of developing CHF increases rapidly with increasing total cumulative doses of epirubicin in excess of 900 mg/m2; this cumulative dose should only be exceeded with extreme caution. Active or dormant cardiovascular disease, prior or concomitant radiotherapy to the mediastinal/pericardial area, previous therapy with other anthracyclines or anthracenediones, or concomitant use of other cardiotoxic drugs may increase the risk of cardiac toxicity

Local effects: Severe local tissue necrosis will occur if there is extravasation during administration. Epirubicin must not be given by the intramuscular or subcutaneous route.

Dermatologic and Hypersensitivity Reactions: Alopecia occurs frequently, but is usually reversible, with hair regrowth occurring within 2 to 3 months from the termination of therapy. Flushes, skin and nail hyperpigmentation, photosensitivity, and hypersensitivity to irradiated skin (radiation-recall reaction) have been observed. Urticaria and anaphylaxis have been reported in patients treated with epirubicin; signs and symptoms of these reactions may vary from skin rash and pruritus to fever, chills, and shock.

Gastrointestinal: A dose-dependent mucositis (mainly oral stomatitis, less often

esophagitis) may occur in patients treated with epirubicin. Clinical manifestations of mucositis may include a pain or burning sensation, erythema, erosions, ulcerations, bleeding, or infections. Mucositis generally appears early after drug administration and, if severe, may progress over a few days to mucosal ulcerations; most patients recover from this adverse event by the third week of therapy. Hyperpigmentation of the oral mucosa may also occur. Nausea,

vomiting, and occasionally diarrhea and abdominal pain can also occur. Severe vomiting and diarrhea may produce dehydration. Antiemetics may reduce nausea and vomiting; prophylactic use of antiemetics should be considered before therapy.

Liver function: The major route of elimination of epirubicin is the hepatobiliary system. Serum total bilirubin and AST levels should be evaluated before and during treatment with epirubicin. Patients with elevated bilirubin or AST may experience slower clearance of drug with an increase in overall toxicity. Lower doses are recommended in these patients

Other: Red discoloration of urine; fever; anaphylactoid reaction; may enhance cyclophosphamide cystitis or mercaptopurine hepatotoxicity

8.7.12 Nursing/Patient Implications

Monitor CBC, platelet counts. Vesicant - do not extravasate. Refer to extravasation protocol if inadvertent infiltration occurs. Advise patient of alopecia. Instruct on how to obtain wig, hairpiece, etc. Hair loss generally occurs 2-4 weeks after injection and is usually complete. Advise patient of red discoloration of urine for 24 hours after administration of the drug. Administer antiemetics as indicated. Assess for stomatitis and treat symptomatically. Generally occurs 7-10 days after injection. Be aware of possible “flare” -most common reaction consists of an erythematous streak up the vein. It is associated with urticaria and pruritus. Occasionally the use of corticosteroids and/or antihistamines has been useful. Monitor for signs and symptoms of cardiomyopathy. Calculate total cumulative dose with each administration.

8.7.13 References

1. Epirubicin (Ellence®) NDA 050778, FDA Approved labeling information 03/02/2005. Accessed at http://www.accessdata.fda.gov/drugsatfda_docs/label/2005/50778s008lbl.pdf
2. [PACKAGE INSERT DATA] : ELLENCE (epirubicin hydrochloride) injection, solution. [Pharmacia and Upjohn Company] Division of Pfizer Inc, NY, NY 10017. Revised: 08/2009

9. Statistical Considerations

9.1 Primary and Secondary Efficacy Evaluations

This trial involves a randomization to a control treatment arm and two experimental treatment arms. The primary objective of this phase III trial is to determine whether the addition of bevacizumab improves disease-free survival (DFS). A two-step hierarchical approach will be used to assess this objective. In the first step, the addition of 10 cycles of maintenance bevacizumab following AC+Bevacizumab->Paclitaxel+Bevacizumab (Arm C) will be compared to AC+placebo->Paclitaxel+placebo(Arm A) (as detailed below). If Arm C significantly improves DFS relative to Arm A, then in the second step, a comparison of Arm B (AC+Bevacizumab->Paclitaxel+ Bevacizumab) to Arm A will be performed.

Secondarily, if the treatment in both Arm C and Arm B significantly improve DFS relative to Arm A, then a comparison of Arm C to Arm B will be performed with respect to DFS (as detailed below).

Disease-free survival, defined as Invasive disease-free survival (IDFS), is calculated from the date of randomization to the date of first treatment failure (invasive ipsilateral, local/regional invasive, or distant recurrence, invasive contralateral breast cancer, invasive non-breast second primary, or death from any cause, whichever occurs first). Cases with incomplete follow up, without documented IDFS event including those who develop squamous or basal cell
Rev. 7/09 skin cancers or in situ carcinomas of any site as their only event will be censored at the date of last disease evaluation.

A total accrual of 4950 patients over the 3 arms is planned where patients are randomized to arms A, B, and C in a 1:2:2 fashion (990 patients randomized to Arm A, 1980 patients randomized to Arm B and 1980 randomized to Arm C).
Rev. 7/09 Treatments will be assigned using permuted blocks within strata. Institutions obtain treatment assignments through the ECOG-ACRIN web registration program. Institutions from other participating groups register through the CTSU who contact the ECOG-ACRIN office to obtain the treatment assignment. Assuming a monthly accrual of 200 patients, 2.0625 years (approximately 25
Rev. 7/09 months) of accrual will be needed. Comparisons will be intention-to-treat analyses among all patients using stratified log rank tests (stratified on the factors outlined in Section 4.)

Based on results from E1199, a 5 year DFS of 80% is assumed for the AC+placebo->Paclitaxel+placebo alone arm (Arm A). Allowing for the interim analysis plan discussed below, with an accrual of 2970 patients to Arms A and C,
Rev. 7/09 there is 80% power using a one sided 0.025 stratified log rank test to detect a 25% reduction in the failure hazard rate. Assuming an exponential distribution, this difference corresponds to an improvement in 5-year DFS from 80% on Arm A to 84.6% in Arm C.

Interim analyses of DFS will be performed for all semi-annual ECOG-ACRIN Data Monitoring Committee (DMC) meetings beginning when approximately 54% (230 failures among all patients within Arms A and C) of the planned full information has been observed, continuing until either the criteria for early stopping are met or full information is reached (426 failures within Arms A and

C). To preserve the overall type I error rate, critical values at the interim analysis will be determined using a truncated version of the Lan-Demets error spending rate function corresponding to the O'Brien-Fleming boundary. Boundary values at a nominal significance level less than 0.0005 will be truncated at 0.0005 with the boundary also adjusted to preserve the overall type I error rate of 2.5%. Under the accrual and failure rate assumptions mentioned above, interim analyses would be expected to occur beginning at approximately 3.2 years after activation when it is anticipated, under the assumption of 200 patients being entered per month, that all patients will have been accrued and completed the chemotherapy/bevacizumab treatment. Interims will then be performed approximately every 6 months until approximately 5.2 years after study activation (for a total of 4 interim analyses and the final analysis). Due to delays in initiation of accrual and delays in data submission and processing, it is likely that the actual analysis times will be 6-12 months later. At full information, there would also be at least 85% power to detect a 26.5% reduction in the failure hazard rate, corresponding to an improvement in 5-year DFS from 80% to 84.9%.

This study will also be monitored for early stopping in favor of the null hypothesis using Jennison-Turnbull repeated confidence interval (RCI) methodology. At each interim analysis, the RCI on the observed hazard ratio will be calculated using the critical value from the error spending rate function. If this RCI does not include the target alternative hazard ratio of 1.33 (Arm A/Arm C) then, assuming that all patients have already been accrued, the ECOG-ACRIN DMC may consider releasing results of lack of benefit from this comparison, but follow-up would continue until the scheduled end of the study.

If criteria for early stopping in favor of the alternative or null hypotheses are not met, then the final analysis for Arm C vs. Arm A will be performed when 426 failures have been observed among all patients within Arm C and Arm A.

Rev. 7/09

If the treatment in Arm C results in significantly improved DFS relative to Arm A, at an interim or at the final analysis, then interim monitoring would begin and be performed approximately twice yearly to assess if Arm B significantly improves DFS relative to Arm A. At each interim, boundaries from the O'Brien-Fleming use function will be used to preserve the overall type I error rate. With an accrual of 2970 patients to Arms A and B, there is 80% power using a one sided 0.025 stratified log rank test to detect a 25% reduction in the failure hazard rate. Assuming an exponential distribution, this difference corresponds to an improvement in 5-year DFS from 80% on Arm A to 84.6% in Arm B. At each interim analysis a RCI will also be calculated to monitor for early stopping in favor of the null hypothesis. If criteria for early stopping in favor of the alternative or null hypothesis are met, then the ECOG-ACRIN DMC may consider releasing results of superiority or lack of benefit from this comparison, but follow-up would continue until the scheduled end of the study. If criteria for early stopping are not met, full information for this comparison also corresponds to 426 failures and is estimated to be reached at 5.2 years from study activation.

To look at duration of bevacizumab, a comparison between Arms C and B with respect to DFS will be performed as a secondary endpoint of interest. This comparison will be performed only after a final determination has been made at an interim or final analysis of significant improvement in DFS in Arm C over Arm A and in Arm B over Arm A. Interim monitoring would begin and be performed

twice yearly to assess if the additional maintenance Bevacizumab in Arm C provides improved DFS.

Rev. 7/09 At each interim, boundaries from the O'Brien-Fleming use function will be used to preserve the overall type I error rate. With 3960 patients, there is 82% power using a one sided 0.025 stratified log rank test to detect a 20% decrease in the failure hazard rate. This difference corresponds to an improvement in 5-year DFS from 84.6% on Arm B to 87.5% on Arm C. Full information corresponds with 702 DFS failures and is expected to be available 7.5 years after activation.

Rev. 7/09 A secondary endpoint for the Arm C vs. A and Arm B vs. A comparisons is survival. The comparison between arms will be made using a stratified log rank test among all randomized patients. Interim analyses for survival for the Arm C vs. A comparison would begin after early stopping or full information had been reached for the Arm C vs. A DFS comparison. Interim analyses for the survival comparison of Arm B to Arm A will be performed twice yearly and would begin only after early stopping or full information for the Arm B vs. A DFS comparison had been reached. Boundaries from the O'Brien-Fleming use function will be used to control the overall type I error rate at a one-sided 0.025 level. Total information for the survival comparison will be 450 deaths within Arm C (Arm B) and Arm A. This will provide 82% power to detect a 25% reduction in the mortality hazard rate, corresponding to a difference in 5-year survival rates of 89% and 91.6%. Under this alternative, full information is expected to be available 9.5 years after activation.

9.2 Safety Monitoring

Expedited adverse event reports will be reviewed continuously by the study chair and by the staff at the ECOG-ACRIN Operations Office - Boston. Summary listings of expedited reports will be reviewed monthly by the study chair, study statistician, and the Breast Committee toxicity monitor. The ECOG-ACRIN Data Monitoring Committee (DMC) will review aggregate summaries of adverse event data at its regular semi-annual meetings. Tabulations of toxicity data will be made available to participating sites twice each year.

Rev. 7/09 In addition to the routine safety monitoring described above, the following specific stopping rules for cardiac toxicity will be used and will also be monitored by the ECOG-ACRIN DMC. Both the decrease in left ventricular ejection fraction (LVEF) of >10% from baseline to post AC +/- Bevacizumab (Cycle 4) with a corresponding Cycle 4 LVEF value below the LLN and the incidence of treatment related clinical CHF (grade 3 or higher) will be monitored formally. LVEF is also scheduled to be assessed at the completion of Paclitaxel +/-Bevacizumab (Cycle 8) and according to the other timepoints as outlined in section 7. Clinical CHF includes patients with symptomatic decline in LVEF to ≤ LLN or symptomatic diastolic dysfunction.

A primary analysis comparing the proportion of patients with both an absolute decrease in LVEF from baseline of >10% and a corresponding Cycle 4 LVEF value below the LLN will be performed when baseline and post Cycle 4 LVEF data are available for the first 300 patients (60 on Arm A, 120 on Arm B, 120 on Arm C) entered who received protocol treatment irrespective of whether classical or dose dense regimen was received. The trial will be suspended if after review of the data there is a significant difference between Arms A vs. C or between

Arms A and B+C with respect to this type of drop in LVEF. Using a one-sided 0.025 level Fisher's Exact Test, there is 80% power to detect a difference of 9% vs. 27% or 18% and 39% between Arms A and C or 80% power to detect a difference of 9% and 25% or 18% and 37% between Arms A vs B+C. At the time of this analysis, a secondary analysis of all patients with available LVEF from baseline and post Cycle 4 will also be performed. Another secondary analysis will look at the baseline and post Cycle 4 LVEF data among the patients who received the dose dense (dd) AC. If only a small proportion of patients have received the dd AC, then an analysis of the first 300 patients who have received dd AC will be performed when those LVEF data become available.

The trial will also be suspended if after review, 4 or more of the first 100 or 6 or more of the first 200 on the combined Arms B and C experience treatment related clinical CHF. If the true rate of clinical CHF is 1% , then the probability of suspending the study is <3%, while if the true rate of clinical CHF is 5%, then the probability of suspending the study is 95%.

Accrual will continue during the evaluation of these stopping rules.

Again, expedited adverse event reports will be reviewed continuously by the study chair and by the staff at the ECOG-ACRIN Operations Office - Boston and monthly summaries reviewed additionally by the study statistician and the Breast Committee toxicity monitor. The ECOG-ACRIN DMC will also review the LVEF data in addition to the general toxicity summaries at the semi annual DMC meetings or between the regularly scheduled meetings as necessary to evaluate the formal stopping rules. Reviews by the DMC of toxicity data for patient safety will continue for the other timepoints that LVEF is scheduled to be assessed as those data become available.

9.3 Gender and Ethnicity

Based on previous data from E2197, the anticipated accrual in subgroups defined by gender and race is:

**Ethnic Category**	**Gender**		
	**Females**	**Males**	**Total**
Hispanic or Latino	113	0	113
Not Hispanic or Latino	4837	0	4837
**Ethnic Category: Total of all subjects**	4950	0	4950
**Racial Category**			
American Indian or Alaskan Native	12	0	12
Asian	65	0	65
Black or African American	368	0	368
Native Hawaiian or other Pacific Islander	2	0	2
White	4390	0	4390
Unknown	113	0	113
**Racial Category: Total of all subjects**	4950	0	4950

The accrual targets in individual cells are not large enough for definitive treatment comparisons to be made within these subgroups. Therefore, overall

accrual to the study will not be extended to meet individual subgroup accrual targets.

Rev. 10/09

9.4 QOL Analysis Plan

The average total score for the FACT-B will be compared between arms at the timepoints outlined in Sections 6.6 and 7 using a T-Test (two-sided, α=0.05). Assuming a standard deviation in the total score of 6 (based on QoL data from e2100), if differences in the average total score between arms are as small as 2.4, there is 82%(89%) power to detect a difference between Arms B and A (Arms B+C vs. A) if QoL assessments are available for 80, 160, and 160 patients on Arms A, B, and C respectively at a given timepoint. If smaller numbers of patients have QOL assessments available at a given timepoint, then only larger differences in the total scores between arms will be detected with at least 80% power. The Wilcoxon Rank Sum test will be used to compare the arms if the distributions of scores are not normally distributed. Longitudinal modeling will be used to look at changes in the scores over time across arms. Utility data derived from the EQ-5D will be used to compute QALYs per patient. Average QALY scores will be compared between (across) arms using t-tests (ANOVA). We will also use estimation approaches that account for the censoring of survival time (102-104). Anticipating compliance rates to vary over the course of the trial at the various timepoints of assessment, QOL assessments will be collected on 500 (n=100, 200,200 on Arms A, B and C) patients who receive treatment and consent to this study. During the 18 month biomarker evaluation, if 20% of 400 patients change their preferred treatment (treatment recommendations), there is 86% power to say that the addition of biomarker information had an impact if there is at least 7% difference between the discordant cell rates of those who would no longer use the regimen and those who now would (two-sided, α=0.05 McNemar's Test). We will also explore the effects of unblinding on breast cancer patients' perception of risk of recurrence and fear of recurrence for those treated by AC/Paclitaxel and bevacizumab or placebo. We hypothesize that after treatment arms are unblinded (17 weeks for dose-dense AC, and 21 weeks for q 3 week AC), patients' perception of risk of recurrence and fear of recurrence will be greater for those in the AC/Paclitaxel and placebo arm vs. AC/Paclitaxel and bevacizumab arm, at the post-unblinding assessment (approximately 18 or 22 weeks post-randomization), resolving by the 54-56 week assessment post-baseline.

9.5 Study Monitoring

This study will be monitored by the ECOG-ACRIN Data Monitoring Committee (DMC). The DMC meets twice each year. For each meeting, all monitored studies are reviewed for safety and progress toward completion. When appropriate, the DMC will also review interim analyses of outcome data. Copies of the toxicity reports prepared for the DMC meetings are included in the study reports prepared for the ECOG-ACRIN group meeting (except that for double blind studies, the DMC may review unblinded toxicity data, while only pooled or blinded data will be made public). These Group Meeting reports are made available to the local investigators, who may provide them to their IRBs. Only the study statistician and the DMC members will have access to interim analyses of outcome data. Prior to completion of this study, any use of outcome data will require approval of the DMC. Any DMC recommendations for changes to this

study will be circulated to the local investigators in the form of addenda to this protocol document. A complete copy of the ECOG-ACRIN DMC Policy can be obtained from the ECOG-ACRIN Operations Office - Boston.

Rev. 10/09

9.6 Pharmacogenetics/Pharmacogenomics Data Analysis

When pharmacogenetics/pharmacogenomics data are available, they will be used first for population stratification assessment. Then they will be correlated with efficacy and toxicity outcomes. The goal of the data analysis is to identify SNP/treatment interactions, i.e. SNPs that predict bevacizumab's efficacy and toxicity. The data analysis will be accomplished through Cox regression model for efficacy variables and the generalized linear model for toxicity outcome variables. Additional gene ontology analyses will be performed to evaluate the functions of significant genes.

Analysis for the association of toxicity with genotype will occur as these results are reported. Genetic effect on efficacy will first be analyzed with the reporting of the first interim analysis as well as with subsequent updates (i.e. other interim analyses and/or final analysis).

10. Correlative Studies

Rev. 2/13
Rev. 10/09

NOTE: REQUIREMENTS FOR PATIENTS PARTICIPATING IN EL112LAB (REGISTERED TO STEP 3), SEE Appendix XV.

Samples are being requested for banking for embedded correlative and future studies and should be submitted only from patients who have given written consent for the use of their samples for these purposes.

The serum and DNA samples will be an incredibly valuable resource for future correlative studies using the emerging genomic, proteomic and pharmacogenomic technologies. Examples of future potential studies include validation of potential predictive markers currently being studied in E2100 (serum VCAM1, VEGF polymorphisms), serum proteomics, analysis of Fc receptor polymorphisms, etc. The samples can't be replaced if not collected prospectively.

NOTE: ECOG-ACRIN requires all samples submitted be logged into the online ECOG-ACRIN Sample Tracking System (STS) as outlined in Section 10.4.

Rev. 12/07

NOTE: Institutions outside of the United States and Canada are not required to submit fresh samples because of the costs and problems associated with international shipping.

Rev. Add14

10.1 Sample Submission Schedule

- All materials are to be collected at baseline, prior to treatment

Rev. 12/07

- Kits for sample collection and shipment are available for sites in the United Stated and Canada. To order kits, follow the instructions in Appendix XIV.Questions regarding kits can be directed to projectmanagement@cenetron.com or call the Cenetron clinical trials group at 512-439-2000.

10.2 Sample Preparation Guidelines

Rev. 12/07

All samples must be labeled with the ECOG-ACRIN protocol number E5103, the patient's ECOG-ACRIN sequence (case) number, patient's initials, collection date and sample type (e.g., serum, plasma EDTA).

Rev. 12/07

10.2.1 Serum, No Anticoagulant (e.g. red top or SST)

- Draw 6-10 mL of blood into one (1) red-top or serum separator vacutainer tube.
- Allow to clot on ice for 30 minutes. Centrifuge for 10 minutes.

Rev. 12/07

- Carefully draw off the serum and place equal aliquots in at least 2 cryovials.
- Freeze and store samples at < -20°C or lower as soon as possible. Samples must be kept frozen until shipping.

Rev. 12/07

10.2.2 Plasma and Cells, EDTA tubes

- Draw one (1) 10 mL or two (2) – 5 mL EDTA tubes, invert 8-10 times
- Centrifuge at 1500 xg (2700 – 3000 rpm) for 15 minutes

- Pipette plasma into 2-4 cryotubes from the kit and store at -20ºC (-70ºC preferred) or lower until time of shipment.
- Remaining Cells: Replace the stopper on the EDTA tube containing the cells. Place **plastic** tube in plastic bag and freeze for shipping with serum and plasma. If plastic vacutainer tubes are not available, ship at ambient temperature on the day of collection.

10.2.3 Tumor Samples

Pathologists and CRAs may also refer to Appendix II "Pathology Submission Guidelines" for the materials required for these samples.

10.2.3.1 Forms and Reports

Rev. 1/15

- STS-generated shipping manifest
- Copy of the Surgical Pathology Report
- In addition to the surgical pathology report, it is necessary that the report documenting the home/reference lab data be forwarded, as well.

10.2.3.2 Biological Material Submissions

- One (1) block of primary tumor
- One (1) block of lymph node positive tumor

Rev. 1/15

NOTE: If blocks are unavailable, contact the ECOG-ACRIN CBPF at (844) 744-2420 to obtain alternative sample submission guidelines.

10.3 <u>Shipping Procedures</u>

A shipping manifest generated from the ECOG-ACRIN STS must accompany all submissions.

Rev. 1/15

Tissue blocks must be submitted at ambient temperature with the pathology/surgical reports. Frozen blood samples must be shipped overnight on dry ice. If dry ice is not available, ship on an adequately frozen Kool-pack

It is recommended that multiple patient samples be batched and shipped together on a quarterly basis. Tissue blocks must be packed so they will NOT freeze if they are submitted with the frozen blood specimens.

Frozen samples shipped overnight must be shipped SUNDAY THROUGH THURSDAY only. Do not ship samples the day before a holiday.

Ship to the ECOG-ACRIN CBPF:

ECOG-ACRIN Central Biorepository and Pathology Facility
MD Anderson Cancer Center
Department of Pathology, Unit 085
Tissue Qualification Laboratory for ECOG-ACRIN, Room G1.3586
1515 Holcombe Blvd
Houston, TX 77030
Phone: Toll Free 844-744-2420 (713-745-4440 Local or International Sites)
Fax: 713-563-6506
Email: eacbpf@mdanderson.org

Rev. 12/07

If the STS is unavailable at time of submission, an E5103 Material Submission Form (#2505) must be submitted with the blood specimens and the laboratory must be notified of overnight shipment. Email a completed Shipment Notification Form (Appendix VIII) to eacbpf@mdanderson.org the day of the shipment. Once STS is available, retroactively log the shipment into STS, using the actual collection and shipping dates.

Rev. 10/09

DNA will be extracted from cells as stated above by the ECOG-ACRIN CBPF. 750 nanograms of DNA will be subsequently shipped to the laboratory of Bryan B. Schneider at Indiana University, Melvin and Bren Simon Cancer Center for candidate SNP genotyping and for execution of the GWAS.

Rev. 10/09

10.3.1 Genotyping and GWAS

The following studies will be performed by Indiana University:

Genome wide association study (GWAS) from DNA Outcome (efficacy and toxicity) will be correlated with SNPs from a genome wide approach in a subgroup of both the control arm and the experimental arm. DNA from the first 2500 patients of the E5103 trial will be genotyped for SNPs and CNV markers using the Infinium Human660W-Quad v1 platform from Illumina. The genotype calls will then undergo quality control (QC) assessment and be statistically correlated with disease free survival (DFS), OS, and toxicity data from E5103 in order to discover novel genetic variants that are predictive biomarkers for bevacizumab in breast cancer.

Rev. 10/09

10.3.2 Candidate single nucleotide polymorphism (SNP) genotyping from DNA

Outcome (efficacy and toxicity) will be correlated with candidate SNPs in both the control arm and the experimental arms in a subgroup of the population as validation. Candidate SNPs will include (1) top associations in the GWAS from the first cohort, (2) functional SNPs from genes identified in the first cohort, (3) imputed SNPs (which would include VEGF based on our preliminary data from E2100), and (4) ancestry informative SNPs. These candidate SNPs will be validated/refuted in the second 2450 patients on two separate 1536 SNP chips using a GoldenGate platform; one for toxicity and one for efficacy.

10.4 ECOG-ACRIN Sample Tracking System

Rev. 2/13

As of June 2007, it is **required** that all samples submitted on this trial be entered and tracked using the ECOG-ACRIN Sample Tracking System (STS). As of June 2007, the software will allow the use of either 1) an ECOG-ACRIN user-name and password previously assigned (for those already using STS), or 2) a CTSU username and password.

When you are ready to log the collection and/or shipment of the samples required for this study, please access the Sample Tracking System software by clicking https://webapps.ecog.org/Tst.

Rev. 12/07
Rev. 1/15

Important: Please note that the STS software creates pop-up windows, so you will need to enable pop-ups within your web browser while using the software. A user manual and interactive demo are available by clicking this link:

Rev. 12/07

http://www.ecog.org/general/stsinfo.html. Please take a moment to get familiar with the software prior to using the system.

A shipping manifest must be generated and shipped with all sample submissions.

Rev. 2/13

If STS is unavailable at time of sample submission, a completed Generic Specimen Submission Form (#2981) is to faxed to the receiving laboratory and included with the sample shipment. When STS becomes available, the submissions must be retroactively logged into the system, using actual submission dates and tracking numbers. Indicate on the submission for the appropriate Lab:

- ECOG-ACRIN CBPF
- FCCC Clinical Protocol Support Lab

Please direct your questions or comments pertaining to the STS to ecog-acrin.tst@jimmy.harvard.edu.

10.5 Banking

Rev. 1/15

The samples collected for this study will be retained at the ECOG-ACRIN CBPF for future use in ECOG-ACRIN approved studies. If future use is denied or withdrawn by the patient, the samples will be removed from consideration for use in any future study.

10.6 Sample Inventory Submission Guidelines

Inventories of all samples collected, aliquoted, and used on the above mentioned laboratory correlative study(ies) will be submitted to the ECOG-ACRIN Operations Office - Boston on a monthly basis. Inventories will be submitted electronically by any laboratory holding and/or using any specimens associated with this study.

11. Records to Be Kept

Please refer to the E5103 Forms Packet for the forms submission schedule and copies of all forms. The E5103 Forms Packet may be downloaded by accessing the ECOG World Wide Web Home Page (http://www.ecog.org). Forms must be submitted to the ECOG-ACRIN Operations Office - Boston, FSTRF, 900 Commonwealth Avenue, Boston, MA 02215 (ATTN: DATA).

This study will be monitored by the CTEP Data Update System (CDUS) version 3.0. Cumulative CDUS data will be submitted quarterly from the ECOG-ACRIN Operations Office - Boston to CTEP by electronic means.

11.1 Records Retention

FDA regulations (21 CFR 312.62) require clinical investigators to retain all trial-related documentation, including source documents, long enough to allow the sponsor to use the data to support marketing applications.

This study will be used in support of a US marketing application (New Drug Application), all records pertaining to the trial (including source documents) must be maintained for:

- two years after the FDA approves the marketing application, or
- two years after the FDA disapproves the application for the indication being studied, or
- two years after the FDA is notified by the sponsor of the discontinuation of trials and that an application will not be submitted.

Please contact the ECOG-ACRIN Operations Office - Boston prior to destroying any source documents.

12. Patient Consent and Peer Judgment

Current FDA, NCI, state, federal and institutional regulations concerning informed consent will be followed.

13. References

1. Jemal A, Murray T, Samuels A, et al: Cancer Statistics. CA Cancer J Clin 53:5-26, 2003
2. Parker S, Tong T, Bolden S, et al: Cancer statistics, 1997. CA Cancer J Clin 47:5-27, 1997
3. Polychemotherapy for early breast cancer: an overview of the randomised trials. Early Breast Cancer Trialists' Collaborative Group. Lancet 352:930-42, 1998
4. Effects of chemotherapy and hormonal therapy for early breast cancer on recurrence and 15-year survival: an overview of the randomised trials. Lancet 365:1687-717, 2005
5. Henderson I, Berry D, Demetri G, et al: Improved disease free and overall survival from the addition of sequential paclitaxelbut not from the escalation of doxorubicin dose level in the adjuvant chemotherapy of patients with node positive primary breast cancer. Proc Am Soc Clin Oncol 17, 1998
6. Nabholtz J, Paterson A, Dirix L, et al: A phase III randomized trial comparing docetaxel, doxorubicin and cyclophosphamide to FAC as first line chemotherapy for patients with metastatic breast cancer. Proc Am Soc Clin Oncol 20:22a, 2001
7. Citron ML, Berry DA, Cirrincione C, et al: Randomized trial of dose-dense versus conventionally scheduled and sequential versus concurrent combination chemotherapy as postoperative adjuvant treatment of node-positive primary breast cancer: first report of Intergroup Trial C9741/Cancer and Leukemia Group B Trial 9741. J Clin Oncol 21:1431-9, 2003
8. Hudis C, Citron M, Berry D, et al: Five year follow-up of INT C9741: Dose-dense chemotherapy is safe and effective. *Breast Cancer Research and Treatment* 94(Suppl 1): abstract 41, 2005.
9. Seidman A, Berry D, Cirrincione C, et al: CALGB 9840: Phase III study of weekly (W) paclitaxel (P) via 1-hour(h) infusion versus standard (S) 3h infusion every third week in the treatment of metastatic breast cancer (MBC), with trastuzumab (T) for HER2 positive MBC and randomized for T in HER2 normal MBC. Journal of Clinical Oncology, 2004 ASCO Annual Meeting Proceedings (Post-Meeting Edition) 22:512, 2004
10. Romond EH, Perez EA, Bryant J, et al: Trastuzumab plus adjuvant chemotherapy for operable HER2-positive breast cancer. N Engl J Med 353(16):1673-84, 2005.
11. Sparano JA, Wang M, Martino S, et al: Phase III study of doxorubicin-cyclophosphamide followed by paclitaxel or docetaxel given every 3 weeks or weekly in patients with axillary node-positive or high-risk node-negative breast cancer: results of North American Breast Cancer Intergroup Trial E1199. San Antonio Breast Cancer Symposium, 2005. http://65.110.82.117/sabcs/index.html
12. Gasparini G: Angiogenesis in breast cancer. Role in biology, tumor progression, and prognosis, in Bowcock A (ed): Breast Cancer: Molecular Genetics, Pathogenesis,and Therapeutics. Totowa, NJ, Humana Press Inc., 1999, pp 347-371
13. Relf M, LeJeune S, Scott PA, et al: Expression of the angiogenic factors vascular endothelial cell growth factor, acidic and basic fibroblast growth factor, tumor growth factor beta-1, platelet-derived endothelial cell growth factor, placenta growth factor,

and pleiotrophin in human primary breast cancer and its relation to angiogenesis. Cancer Res 57:963-9, 1997

14. Brem SS, Gullino PM, Medina D: Angiogenesis: a marker for neoplastic transformation of mammary papillary hyperplasia. Science 195:880-2, 1977
15. Jensen HM, Chen I, DeVault MR, et al: Angiogenesis induced by "normal" human breast tissue: a probable marker for precancer. Science 218:293-5, 1982
16. Kurebayashi J, McLeskey SW, Johnson MD, et al: Quantitative demonstration of spontaneous metastasis by MCF-7 human breast cancer cells cotransfected with fibroblast growth factor 4 and LacZ. Cancer Res 53:2178-87, 1993
17. McLeskey SW, Zhang L, Kharbanda S, et al: Fibroblast growth factor overexpressing breast carcinoma cells as models of angiogenesis and metastasis. Breast Cancer Res Treat 39:103-17, 1996
18. McLeskey SW, Tobias CA, Vezza PR, et al: Tumor growth of FGF or VEGF transfected MCF-7 breast carcinoma cells correlates with density of specific microvessels independent of the transfected angiogenic factor. Am J Pathol 153:1993-2006, 1998
19. Zhang L, Chang C, Bacus S, et al: Suppressed transformation and induced differentiation of HER-2/neu-overexpressing breast cancer cell by emodin. Cancer Res 55:3890-3896, 1995
20. Nakajima M, Welch DR, Wynn DM, et al: Serum and plasma M(r) 92,000 progelatinase levels correlate with spontaneous metastasis of rat 13762NF mammary adenocarcinoma. Cancer Res 53:5802-7, 1993
21. Giunciuglio D, Culty M, Fassina G, et al: Invasive phenotype of MCF10A cells overexpressing c-Ha-ras and c-erbB-2 oncogenes. Int J Cancer 63:815-822, 1995
22. Weinstat-Saslow DL, Zabrenetzky VS, VanHoutte K, et al: Transfection of thrombospondin 1 complementary DNA into a human breast carcinoma cell line reduces primary tumor growth, metastatic potential, and angiogenesis. Cancer Res 54:6504-11, 1994
23. Wang M, Liu YE, Greene J, et al: Inhibition of tumor growth and metastasis of human breast cancer cells transfected with tissue inhibitor of metalloproteinase 4. Oncogene 14:2767-74, 1997
24. Liotta LA, Steeg PS, Stetler-Stevenson WG: Cancer metastasis and angiogenesis: an imbalance of positive and negative regulation. Cell 64:327-36, 1991
25. Guinebretiere JM, Le Monique G, Gavoille A, et al: Angiogenesis and risk of breast cancer in women with fibrocystic disease [letter; comment]. J Natl Cancer Inst 86:635-6, 1994
26. Guidi AJ, Fischer L, Harris JR, et al: Microvessel density and distribution in ductal carcinoma in situ of the breast. J Natl Cancer Inst 86:614-9, 1994
27. Engels K, Fox SB, Whitehouse RM, et al: Distinct angiogenic patterns are associated with high-grade in situ ductal carcinomas of the breast. J Pathol 181:207-12, 1997
28. Guidi AJ, Schnitt SJ, Fischer L, et al: Vascular permeability factor (vascular endothelial growth factor) expression and angiogenesis in patients with ductal carcinoma in situ of the breast. Cancer 80:1945-53, 1997
29. Weidner N, Semple J, Welch W, et al: Tumor angiogenesis and metastasis - correlation in invasive breast cancer. N Engl J Med 324:1-8, 1991

30. Weidner N, Folkman J, Pozza F, et al: Tumor angiogenesis: a new significant and independent prognostic indicator in early stage breast carcinoma. J Natl Cancer Inst 84:1875-1887, 1992
31. Horak E, Leek R, Klenk N: Angiogenesis, assessed by platelet/endothelial cell adhesion molecule antibodies, as indicator of node metastasis and survival in breast cancer. Lancet 340:1120-1124, 1992
32. Martin L, Green B, Renshaw C, et al: Examining the technique of angiogenesis assessment in invasive breast cancer. Br J Cancer 76:40-43, 1997
33. Simpson J, Ahn C, Battifora H, et al: Endothelial area as a prognostic indicator for invasive for invasive breast carcinoma. Cancer 77:2077-2085, 1996
34. Toi M, Inada K, Suzuki H, et al: Tumor angiogenesis in breast cancer: its importance as a prognostic indicator and the association with vascular endothelial growth factor expression. Breast Cancer Res Treat 36:193-204, 1995
35. Bevilacqua P, Barbareschi M, Verderio P, et al: Prognostic value of intratumoral microvessel density, a measure of tumor angiogenesis, in node-negative breast carcinoma--results of a multiparametric study. Breast Cancer Res Treat 36:205-17, 1995
36. Hall N, Fish D, Hunt N, et al: Is the relationship between angiogenesis and metastasis in breast cancer real? Surg Oncol 1:223-229, 1992
37. McCulloch P, Choy A, Martin L: Association between tumour angiogenesis and tumour cell shedding into effluent venous blood during breast cancer surgery [see comments]. Lancet 346:1334-5, 1995
38. Fox S, Leek R, Bliss J, et al: Association of tumor angiogenesis with bone marrow micrometastases in breast cancer patients. J Natl Cancer Inst 89:1044-1049, 1997
39. Ferrara N, Davis-Smyth T: The biology of vascular endothelial growth factor. Endocrinol Rev 1997:1-22, 1997
40. Gasparini G, Toi M, Gion M, et al: Prognostic significance of vascular endothelial growth factor protein in node-negative breast carcinoma. J Natl Cancer Inst 89:139-47., 1997
41. Linderholm B, Tavelin B, Grankvist K, et al: Vascular endothelial growth factor is of high prognostic value in node- negative breast carcinoma. J Clin Oncol 16:3121-8, 1998
42. Toi M, Kondo S, Suzuki H, et al: Quantitative analysis of vascular endothelial growth factor in primary breast cancer. Cancer 77:1101-6, 1996
43. Toi M, Hoshina S, Takayanagi T, et al: Association of vascular endothelial growth factor expression with tumor angiogenesis and with early relapse in primary breast cancer. Jpn J Cancer Res 85:1045-1049, 1994
44. Eppenberger U, Kueng W, Schlaeppi JM, et al: Markers of tumor angiogenesis and proteolysis independently define high- and low-risk subsets of node-negative breast cancer patients. J Clin Oncol 16:3129-36, 1998
45. Warren RS, Yuan H, Matli MR, et al: Regulation by vascular endothelial growth factor of human colon cancer tumorigenesis in a mouse model of experimental liver metastasis. J Clin Invest 95:1789-97, 1995
46. Kim KJ, Li B, Winer J, et al: Inhibition of vascular endothelial growth factor-induced angiogenesis suppresses tumour growth in vivo. Nature 362:841-4, 1993

47. Gordon MS, Margolin K, Talpaz M, et al: Phase I safety and pharmacokinetic study of recombinant human anti- vascular endothelial growth factor in patients with advanced cancer. J Clin Oncol 19:843-50., 2001
48. Sledge G, Miller K, Novotny W, et al: A phase II trial of single-agent rhuMAb VEGF (recombinant humanized monoclonal antibody to vascular endothelial cell growth factor) in patients with relapsed metastatic breast cancer. Proc Am Soc Clin Oncol 19:3a, 2000
49. Cobleigh M, Miller K, Langmuir V, et al: Phase II dose escalation trial of Avastin (bevacizumab) in women with previously treated metastatic breast cancer. Breat Cancer Res Treat 69:301, 2001
50. Miller K, Rugo H, Cobleigh M, et al: Phase III trial of capecitabine (Xeloda) plus bevacizumab (Avastin) versus capecitabine alone in women with metastatic breast cancer previously treated with an anthracycline and a taxane. Breast Cancer Res Treat 76:S37, 2002
51. Karp JE, Gojo I, Pili R, et al: Targeting vascular endothelial growth factor for relapsed and refractory adult acute myelogenous leukemias: therapy with sequential 1-beta-d-arabinofuranosylcytosine, mitoxantrone, and bevacizumab. Clin Cancer Res 10:3577-85, 2004
52. Wedam S, Low J, Yang X, et al: A pilot study to evaluate response and angiogenesis after treatment with bevacizumab in patients with inflammatory breast cancer. Proc Am Soc Clin Oncol 23:21, 2004
53. D'Adamo D, Anderson S, Albritton K, et al: Cardiac toxicity in a phase II study of doxorubicin and bevacizumab for patients with metastatic soft tissue sarcoma. Proc Am Soc Clin Oncol 23:817, 2004
54. Burstein H, Parker L, Savoie J, et al: Phase II trial of the anti-VEGF antibody bevacizumab in combination with vinorelbine for refractory advanced breast cancer. Breast Cancer Res Treat 76:S115, 2002
55. Hudis C, Barlow W, Costantino J, et al. Endpoints defined: Proposal for standardized definitions for efficacy endpoints in adjuvant breast cancer trials: The STEEP System. J Clin Oncol 25:2127-2132, 2007
56. Aneshensel CS, Frerichs RR, Clark VA, Yokopenic PA. Telephone versus in-person surveys of community health status. American Journal of Public Health 1982; 72: 1017-1021.
57. Brady MJ, Cella DF, Mo F, Bonomi AE, Tulsky DS, Lloyd SR, Deasy S, Cobleigh M, Shiomoto G. Reliability and validity of the Functional Assessment of Cancer Therapy-Breast Quality-of-Life instrument. Journal of Clinical Oncology 1997; 15: 974-986.
58. Brucker PS, Yost K, Cashy J, et al: General population and cancer patient norms for the Functional Assessment of Cancer Therapy General (FACT-G). Eval Health Prof. 28:192-211, 2005.
59. Cella DF, Tulsky DS, Gray G, et al. The Functional Assessment of Cancer Therapy Scale: Development and validation of the general measure. Journal of Clinical Oncology 1993: 11: 570-579.
60. Cella DF. F.A.C.I.T. Manual: Manual of the Functional Assessment of Chronic Illness Therapy Scales. Version 4. Chicago, IL: Center on Outcomes, Research and Education (CORE), Evanston Northwestern Healthcare and Northwestern University, November 1997.

61. Chang VT, Thaler HT, Polyak TA, Kornblith AB, Lepore JM, Portenoy RK. Quality of life and survival: The role of multidimensional symptom assessment. Cancer 1998; 83: 173-179.
62. Colombotos J. Personal versus telephone interviews: Effect on responses. Public Health Report 1969; 84: 773-782.
63. Coster S, Poole K, Fallowfield LJ. The validation of a quality of life scale to assess the impact of arm morbidity in breast cancer patients post-operatively. Breast Cancer Research and Treatment 2001; 68: 273-282.
64. Duric VM, Stockler MR, Heritier S, Boyle F, Beith J, Sullivan A, et al. Patients' preferences for adjuvant chemotherapy in early breast cancer: what makes AC and CMF worthwhile now? Ann Oncol 2005;16(11):1786-94.
65. Ferrans, CE, Ryan C, Archer LE, Freels S, Lan L, Paskett E, Molokie R, Hurd DD, Kornblith A. Factors contributing to cancer screening in African Americans. Proc Am Soc Oncol, 2008; 26: [Abstract 6514].
66. Fernig S, Levac I, Kohn R, Yelin N. Telephone vs face-to-face interviewing in a community psychiatric survey. American Journal of Public Health 1993; 83: 896-898.
67. Greenberg DB, Kornblith AB, Herndon JE, Zuckerman E, Schiffer CA, Weiss RB, Mayer RJ, Wolchok SM, Holland JC. Quality of life of adult leukemia survivors treated on clinical trials of the Cancer and Leukemia Group B from 1971-1988: Predictors for later psychological distress. Cancer 1997; 80: 1936-1944.
68. Groves R, Kahn M: Surveys by telephone: A national comparison with personal interviews. New York: Academic Press, 1979.
69. Hill JM, Kornblith AB, Jones D, Freeman A, Holland JF, Glicksman AS, Boyett JM, Lenherr B, Bricher M, Dubowy R, Kung F, Maurer H, Holland JC. A comparative study of the long-term psychosocial functioning of childhood acute leukemia survivors treated by intrathecal methotrexate with and without cranial radiation. Cancer 1998; 82:208-218.
70. Hochstim JR: A critical comparison of three strategies of collecting data from households. Journal of the American Statistical Association 1967; 62: 976-989.
71. Holland JC, Herndon J, Kornblith AB, Cella DF, Cooper MR, Green M, Ozer H, Silver RT, Clamon G, Maurer H. A sociodemographic data collection model for cooperative clinical trials [Abstract]. Proc ASCO 1992; 11: No. 445, p.157.
72. Johnson JA, Coons SJ, Ergo A, Szava-Kovats G. Valuation of EuroQOL (EQ-5D) health states in an adult US sample. Pharmacoeconomics 1998; 13: 421-433.
73. Jordan LA, Marcus AC, Reeder LG. Response styles in telephone and household interviewing: A field experiment. Public Opinion Quarterly 1980; 44: 210-222.
74. Kopec JA, Richardson CG, Llewellyn-Thomas H, Klinkhoff A, Carswell A, Chalmers A. Probabilistic threshold technique showed that patients' preferences for specific trade-offs between pain relief and each side effect of treatment in osteoarthritis varied. J Clin Epidemiol. 2007 Sep;60(9):929-38. Epub 2007 May 8.
75. Kornblith AB, Holland JC. A model for quality-of-life research from the Cancer and Leukemia Group B: The telephone interview, conceptual approach to measurement, and theoretical framework. Journal of the National Cancer Institute Monograph 1996; 20:55-62.

76. Kornblith AB, Herndon JE, Zuckerman E, Viscoli C, Horwitz R, Cooper MR, Harris L, Tkaczuk KH, Perry MC, Budman D, Norton L, Holland JC. Social support as a buffer to the psychological impact of stressful life events in women with breast cancer. Cancer 2001; 91: 443-454.

77. Kornblith AB, Herndon JE, Silverman LR, Demakos EP, Odchimar-Reissig R, Holland JF, Powell BL, DeCastro C, Ellerton J, Larson RA, Schiffer CA, Holland JC. The impact of 5-Azacytidine on the quality of life of patients with Myelodysplastic Syndrome [MDS] treated in a randomized Phase III trial of the Cancer and Leukemia Group B [CALGB]. Journal of Clinical Oncology 2002; 20:2441-2452.

78. Kornblith AB, Herndon JE, Weiss RB, Zhang, C, Zuckerman EL, Rosenberg S, Mertz M, Payne D, Massie MJ, Holland JF, Wingate P, Norton L, Holland JC. Long-term adjustment of survivors of early stage breast cancer 20 years after adjuvant chemotherapy. Cancer 2003; 98:679-689.

79. Kornblith AB, Dowell JM, Herndon JE II, Engelman B, Bauer-Wu S, Small EJ, Morrison VA, Atkins J, Cohen HJ, Holland JC. Telephone monitoring of distress in advanced stage cancer patients 65 or more years old: A Cancer and Leukemia Group B Study. Cancer 2006; 107: 2706-2714.

80. Kornblith AB, Herndon J, Zuckerman E, Godley PA, Savarese D, Vogelzang NJ, Holland JC. The impact of docetaxel, estramustine, and low dose hydrocortisone on the quality of life of men with hormone refractory prostate cancer (HRPC) and their partners: A feasibility study. Annals of Oncology 2001; 12: 633-641.

81. Kornblith AB, Thaler H, Wong G, Vlamis V, McCarthy-Lepore J, Loseth D, Hakes T, HoskinsWJ, Portenoy RK. Quality of life of women with ovarian cancer. Gynecologic Oncology 1995; 59: 231-242.

82. Kornblith AB, Powell M, Regan MM, Bennett S, Krasner C, Moy B, Younger J, Goodman A, Berkowitz R, Winer E. Long-term psychosocial adjustment of older vs. younger survivors of breast and endometrial cancer. Psycho-Oncology 2007; 16: 895-903.

83. Matulonis UA, Kornblith A, Hang L, Bryan J, Gibson C, Wells C, Lee J, Sullivan L, Penson R. Long-term adjustment of early-stage ovarian cancer survivors. International Journal of Gynecologic Cancer 2008; 18: 1183-1193.

84. Mehta SS, Lubeck DP, Pasta DJ, Litwin MS. Fear of cancer recurrence in patients undergoing definitive treatment for prostate cancer: Results from CaPSURE. Journal of Urology 2003; 170: 1931-1933.

85. Miller K, Rugo H, Cobleight M et al. Phase III trial of capecitabine (Xeloda) plus bevacizumab (Avastin) versus capecitabine alone in women with metastatic breast cancer previously treat ed with an anthracycline and a taxane. Breast Cancer Research and Treat ment 2002; 76:S37.

86. Paulsen AS, Crowe RR, Noyes R, Pfohl B. Reliability of the telephone interview in diagnosing anxiety disorders. Archives of General Psychiatry 1988; 45; 62-63.

87. Portenoy RK, Thaler HT, Kornblith AB, Lepore JM, Friedlander-Klar H, Kiyasu E, Sobel K,Coyle N, Kemeny N, Norton L, Scher H. The Memorial Symptom Assessment Scale: An instrument for the evaluation of symptom prevalence, characteristics, and distress. European Journal of Cancer 1994; 30A (9):1326-1336. [a]

88. PortenoyRK, Thaler HT, Kornblith AB, Lepore JM, Friedlander-Klar H, Coyle N, Kemeny N,Smart-Curley T, Kemeny N, Norton L, Hoskins W, Scher H. Symptom

prevalence,characteristics and distress in a cancer population. Quality of Life Research 1994; 3: 183-189.[b]

89. Rabin R, de Charro. EQ-5D: a measure of health status from the EurpQol Group. Annals of Medicine 2001; 33: 337-343.
90. Ravdin PM, Siminoff IA, Harvey JA. Survey of breast cancer patients concerning their knowledge and expectations of adjuvant therapy. J Clin Oncol. 1998 Feb;16(2):515-21.
91. Shaw JW, Johnson JA, Coons SJ. US valuation of the EQ-5D health states: Development and testing of the D1 valuation model. Medical Care 2005; 43: 203-220.
92. Siemiatycki J. A comparison of mail, telephone and home interview strategies for houselhold health surveys. Amer J Public Health 1979; 69: 238-245.
93. Simes RJ, Coates AS. Patient preferences for adjuvant chemotherapy of early breast cancer: how much benefit is needed? J Natl Cancer Inst Monogr 2001(30):146-52.
94. Sobin C, Weissman MM, Goldstein RB, Adams P, Wickramaratne P, Warner V, Lish JD. Diagnostic interviewing for family studies: comparing telephone and face-to-face methods for the diagnosis of lifetime psychiatric disorders. Psychiatric Genetics 1993; 3: 227233.
95. Thewes B, Meiser B, Duric VM, Stockler MR, Taylor A, Stuart-Harris R, et al. What survival benefits do premenopausal patients with early breast cancer need to make endocrine therapy worthwhile? Lancet Oncol 2005;6(8):581-8.
96. Weinstein ND, Kwitel A, McCaul KD, Magnan RE, Gerrard M, Gibbons FX. Risk perceptions: assessment and relationship to influenza vaccination. Health Psychol. 2007 Mar;26(2):146-51.
97. Wells KB, Burham A, Leake B, Robins LN. Agreement between face-to-face and telephone administered versions of the depression section of the NIMH Diagnostic Interview Schedule. J Psychiatric Res 1988; 22: 207-220.
98. Woloshin S, Schwatz L, Byrum, Fischoff B and Welch G. (2000). A new scale for assessing perceptions of chance. Medical Decision Making. 20: 298-307.
99. Zikmund-Fisher BJ, Couper M, Singe E, Ubel P, Ziniel S, Fowler FJ, Levin C and Fagerlin A. Prevalence and process of medical decision making in the US: Reports from the DECISIONS survey. 2008. Presented at 30th Annual Meeting Society for Medical Decision Making.
100. Zikmund-Fisher BJ, Fagerlin A, Ubel PA. Improving understanding of adjuvant therapy options by using simpler risk graphics. Cancer. 2008 Dec 15;113(12):3382-90.
101. Zikmund-Fisher BJ, Ubel PA, Smith DM, Derry HA, McClure JB, Stark A, Pitsch RK, Fagerlin A. Communicating side effect risks in a tamoxifen prophylaxis decision aid: the debiasing influence of pictographs. Patient Educ Couns. 2008 Nov;73(2):209-14.
102. Glasziou, P.P., Cole, B.F., Gelber, R.D., Hilden, J., and Simes, R.J. (1998). Quality-adjusted survival analysis with repeated quality-of-life measures. /Statistics in Medicine /*17*, 1215–1229.
103. Zhao, Hongwei and Tsiatis, Anastasios A. (2001) Testing equality of survival functions of quality-adjusted lifetime */Biometrics/*, 57, 861-867

104. Zhao, Hongwei and Tsiatis, Anastasios A. (2000) Estimating mean quality adjusted lifetime with censored data */Sankhya-, Series B/*, 62, 175-188

105. Jordan LA, Marcus AC, Reeder LG. Response styles in telephone and household interviewing: A field experiment. Public Opinion Quarterly 1980; 44: 210-222.

A Double-Blind Phase III Trial of Doxorubicin and Cyclophosphamide followed by Paclitaxel with Bevacizumab or Placebo in Patients with Lymph Node Positive and High Risk Lymph Node Negative Breast Cancer

Rev. 3/13

Appendix I

Suggested Patient Consent Form [Deleted in Update #8]

INFORMED CONSENT INTENTIONALLY REMOVED FROM PROTOCOL DOCUMENT

Appendix I was removed from the protocol document in Update #8 and is posted as a separate document on the ECOG website. This was removed from the protocol to comply with NCI formatting guidelines

A Double-Blind Phase III Trial of Doxorubicin and Cyclophosphamide followed by Paclitaxel with Bevacizumab or Placebo in Patients with Lymph Node Positive and High Risk Lymph Node Negative Breast Cancer [Deleted in]

Rev. 3/13

Appendix I-A

EL112LAB: North American Breast Cancer Groups Biospecimen Bank for Determinants of Late Relapse in Operable Breast Cancer Suggested Patient Consent Form [Deleted in Update #8]

INFORMED CONSENT INTENTIONALLY REMOVED FROM PROTOCOL DOCUMENT

Appendix I-A was inserted into the protocol in Addendum #10.

Appendix I-A was removed from the protocol document in Update #8 and is posted as a separate document on the ECOG website. This was removed from the protocol to comply with NCI formatting guidelines.

A Double-Blind Phase III Trial of Doxorubicin and Cyclophosphamide followed by Paclitaxel with Bevacizumab or Placebo in Patients with Lymph Node Positive and High Risk Lymph Node Negative Breast Cancer

Appendix II
Pathology Submission Guidelines

The following items are included in Appendix II:

1. Guidelines for Submission of Pathology Materials
   (instructional sheet for Clinical Research Associates [CRAs])

Guidelines for Submission of Pathology Materials

Rev. 1/15

The following items should always be included when submitting pathology materials to the ECOG-ACRIN Central Biorepository and Pathology Facility:

- Institutional Surgical Pathology Report
- Pathology materials (see attached List of Required Material)
- ECOG-ACRIN Generic Specimen Submission Form (#2981)

Instructions:

Rev. 1/15

1. Provide the following information to the pathologist

   Patient's name (last, first)

   Protocol number

   Protocol case number (the patient's ECOG-ACRIN sequence number; for intergroup studies, include both the ECOG-ACRIN and other group's sequence numbers)

   Patient's hospital number

   Institution

   Affiliate (if appropriate)

2. Complete blank areas of the pathologist's instructional memo and forward it, along with the List of Required Material to the appropriate pathologist.
3. The pathologist should return the required pathology samples and surgical pathology reports. If any other reports are required, they should be obtained from the appropriate department at this time. The ECOG-ACRIN Generic Specimen Submission Form (#2981) may be used for record keeping and communicating information internally, but is not required to be submitted unless STS is unavailable.
4. Keep a copy of the ECOG-ACRIN Generic Specimen Submission Form (#2981) or STS Shipping Manifest for your records.
5. Double-check that ALL required forms, reports and pathology samples are included in the package to the Central Biorepository and Pathology Facility. (See appropriate List of Required Material.)
   Pathology specimens submitted WILL NOT be processed by the Central Biorepository and Pathology Facility until all necessary items are received.
6. Mail pathology materials to:

   ECOG-ACRIN Central Biorepository and Pathology Facility
   MD Anderson Cancer Center
   Department of Pathology, Unit 085
   Tissue Qualification Laboratory for ECOG-ACRIN, Room G1.3586
   1515 Holcombe Blvd
   Houston, TX 77030
   Phone: Toll Free 844-744-2420 (713-745-4440 Local or International Sites)
   Fax: 713-563-6506
   Email: eacbpf@mdanderson.org

Rev. 1/15

If you have any questions concerning the above instructions or if you anticipate any problems in meeting the pathology material submission deadline of one month, contact the Pathology Coordinator at the ECOG-ACRIN Central Biorepository and Pathology Facility by telephone (844) 744-2420 or by email eacbpf@mdanderson.org.

List of Required Material

E5103: *A Double-Blind Phase III Trial of Doxorubicin and Cyclophosphamide followed by Paclitaxel with Bevacizumab or Placebo in Patients with Lymph Node Positive and High Risk Lymph Node Negative Breast Cancer*

Rev. 2/13
Rev. 1/15

Requirements for patients registered to Step 1

Submit from all patients who have consented to participation in the protocol-defined laboratory research studies or banking for future research.

Original Diagnostic Pathology Specimens

The following materials are to be submitted:

1. Institutional pathology report **(must be included with EVERY pathology submission)**.

Rev. 7/10
Rev. 1/15

2. Required pathology materials:
    - One (1) block of primary tumor
    - One (1) block of lymph node positive tumor

NOTE: If blocks are unavailable, contact the ECOG-ACRIN CBPF at (844) 744-2420 to obtain altenative sample submission guidelines.

Rev. 2/13
Rev. 1/15

Requirements for patients registered to Step 3 (Ancillary study EL112LAB)

The following is to be submitted from all patients registered to Step 3. If blocks are unavailable, contact the ECOG-ACRIN CBPF at (844) 744-2420 to obtain altenative sample submission guidelines.

Original Diagnostic Pathology Specimens (REQUIRED only if not previously submitted)

The following materials are to be submitted:

1. Institutional pathology report **(must be included with EVERY pathology submission)**.
2. Required pathology materials:
    - One (1) block of primary tumor
    - One (1) block of lymph node positive tumor

Rev. 2/13
Rev. 1/15

Recurrence

1. Institutional pathology report **(must be included with EVERY pathology submission)**.
2. Required pathology materials:
    - One (1) block from diagnostic biopsy

ECOG-ACRIN
cancer research group
Reshaping the future of patient care

Robert L. Comis, MD, and Mitchell D. Schnall, MD, PhD
Group Co-Chairs

MEMORANDUM

TO: ____
(Submitting Pathologist)

FROM: Stanley Hamilton, M.D., Chair
ECOG-ACRIN Laboratory Science and Pathology Committee

DATE: ____________________________________

RE: Submission of Pathology Materials for E5103: *A Double-Blind Phase III Trial of Doxorubicin and Cyclophosphamide followed by Paclitaxel with Bevacizumab or Placebo in Patients with Lymph Node Positive and High Risk Lymph Node Negative Breast Cancer*

The patient named on the attached request has been entered onto an ECOG-ACRIN protocol by ______________________________ (ECOG-ACRIN Investigator). This protocol requests the submission of pathology materials for research studies.

Rev. 2/13
Rev.1/15

Keep a copy of the submission for your records. Forward the surgical pathology report(s), the slides and/or blocks and any other required material (see List of Required Material) to the Clinical Research Associate (CRA). The CRA will forward all required pathology material to the ECOG-ACRIN Central Biorepository and Pathology Facility.

Blocks and slides submitted for this study will be retained at the ECOG-ACRIN Central Repository for future studies. Blocks may be available for purposes of patient management upon written request. However, since samples are submitted for laboratory studies, there may be no material left to return.

If you have any questions regarding this request, please contact the Central Biorepository and Pathology Facility at (844) 744-2420 or email eacbpf@mdanderson.org.

The ECOG-ACRIN CRA at your institution is:

Name:____

Address:_____________________________________

Phone: ______________________________________

Thank you.

ECOG-ACRIN Generic Specimen Submission Form

Form No. 2981v3

Institution Instructions: This form is to be completed and submitted with **all specimens** ONLY if the Sample Tracking System (STS) is not available. **Use one form per patient, per time point.** All specimens shipped to the laboratory must be listed on this form. Enter all dates as MM/DD/YY. Keep a copy for your files. Retroactively log all specimens into STS once the system is available. **Contact the receiving lab to inform them of shipments that will be sent with this form.**

Protocol Number ______________________ **Patient ID** ______________________ **Patient Initials** Last ________ First ________

Date Shipped ______________________ **Courier** ______________________ **Courier Tracking Number** ______________________

Shipped To (Laboratory Name) ______________________ **Date CRA will log into STS** ______________________

FORMS AND REPORTS: Include all forms and reports as directed per protocol, e.g., pathology, cytogenetics, flow cytometry, patient consult, etc.

**Required fields for all samples**				**Additional fields for tissue submissions**				**Completed by Receiving Lab**
**Protocol Specified Timepoint:**								
**Sample Type** (fluid or fresh tissue, include collection tube type)	**Quantity**	**Collection Date and Time 24 HR**		**Surgical or Sample ID**	**Anatomic Site**	**Disease Status** (e.g., primary, mets, normal)	**Stain or Fixative**	**Lab ID**

**Fields to be completed if requested per protocol. Refer to the protocol-specific sample submissions for additional fields that may be required.**					
**Leukemia/Myeloma Studies:**	**Diagnosis**	**Intended Treatment Trial**	**Peripheral WBC Count (x1000)**	**Peripheral Blasts %**	**Lymphocytes %**
**Study Drug Information:**	**Therapy Drug Name**	**Date Drug Administered**	**Start Time 24 HR**	**Stop Time 24HR**	
**Caloric Intake:**	**Date of Last Caloric Intake**		**Time of Last Caloric Intake 24HR**		

CRA Name ______________________ **CRA Phone** ______________________ **CRA Email** ______________________

Comments

9/12/14

A Double-Blind Phase III Trial of Doxorubicin and Cyclophosphamide followed by Paclitaxel with Bevacizumab or Placebo in Patients with Lymph Node Positive and High Risk Lymph Node Negative Breast Cancer

Appendix III

Patient Thank You Letter

We ask that the physician use the template contained in this appendix to prepare a letter thanking the patient for enrolling in this trial. The template is intended as a guide and can be downloaded from the ECOG web site at http://www.ecog.org. As this is a personal letter, physicians may elect to further tailor the text to their situation.

This small gesture is a part of a broader program being undertaken by ECOG-ACRIN and the NCI to increase awareness of the importance of clinical trials and improve accrual and follow-through. We appreciate your help in this effort.

[PATIENT NAME] [DATE]

[PATIENT ADDRESS]

Dear [PATIENT SALUTATION],

Thank you for agreeing to take part in this important research study. Many questions remain unanswered in cancer. With the help of people like you who participate in clinical trials, we will achieve our goal of effectively treating and ultimately curing cancer.

We believe you will receive high quality, complete care. Your physician and research staff will maintain very close contact with you. This is important so as to allow your physician to provide you with the best care while learning as much as possible to help you and other patients.

On behalf of ***[INSTITUTION]*** and the ECOG-ACRIN Cancer Research Group, we thank you again and look forward to helping you.

Sincerely,

[PHYSICIAN NAME]

A Double-Blind Phase III Trial of Doxorubicin and Cyclophosphamide followed by Paclitaxel with Bevacizumab or Placebo in Patients with Lymph Node Positive and High Risk Lymph Node Negative Breast Cancer

Appendix IV

Evaluation of Cardiac Toxicity

To evaluate the benefits and risks of continuing bevacizumab, two goals must be balanced:

1. the protection of patients from serious myocardial toxicity and
2. the ability to assess the potential benefit of bevacizumab in patients with node-positive breast cancer

The following guidelines are recommended:

- Schedule each patient's MUGA scan/echocardiogram at the same radiology facility throughout the study.
- There are several conditions wherein a repeat MUGA scan/echocardiogram 6 weeks later is required. Please refer to section 5.5. See section 4.9 for MUGA/echocardiogram reimbursement information.

In some patients, anthracycline-induced myocardial dysfunction may occur months after doxorubicin is discontinued. Therefore, it is important to monitor myocardial function throughout the study to determine whether, and to what degree, there may be additional myocardial toxicity associated with bevacizumab in the adjuvant setting.

In the decision to continue or stop bevacizumab in an asymptomatic patient, both the specific ejection fraction and the change in ejection fraction from baseline must be considered.

Patients should be monitored for signs and symptoms of congestive heart failure (CHF) (i.e., dyspnea, tachycardia, cough, neck vein distension, cardiomegaly, hepatomegaly, paroxysmal nocturnal dyspnea, orthopnea, peripheral edema, etc.). Patients who develop these signs and symptoms must permanently discontinue bevacizumab.

A Double-Blind Phase III Trial of Doxorubicin and Cyclophosphamide followed by Paclitaxel with Bevacizumab or Placebo in Patients with Lymph Node Positive and High Risk Lymph Node Negative Breast Cancer

Appendix V

Procedure for Obtaining a Urine Protein/Creatinine Ratio

1. Obtain at least 4 mL of a random urine sample (does not have to be a 24 hour urine)
2. Determine protein concentration (mg/dL)
3. Determine urine creatinine concentration (mg/dL)
4. Divide #2 by #3 above:

$$\text{Urine protein/creatinine ratio} = \frac{\text{Protein concentration (mg/dL)}}{\text{Creatinine concentration (mg/dL)}}$$

The UPC directly correlates with the amount of protein excreted in the urine per 24 hrs (i.e. a UPC of 1 should be equivalent to 1g protein in a 24hr urine collection)

Rev. 7/09 If protein and creatinine concentrations are not routinely reported at an Institution, their measurements and reports may need to be requested.

Rev. 7/09 **NOTE:** Complete urinalysis is not required. Only the urine PRT/CRT is needed to calculate the UPC.

A Double-Blind Phase III Trial of Doxorubicin and Cyclophosphamide followed by Paclitaxel with Bevacizumab or Placebo in Patients with Lymph Node Positive and High Risk Lymph Node Negative Breast Cancer

Appendix VI

Breast Cancer Clinical Trial Announcement

A Phase III Trial of Doxorubicin and Cyclophosphamide followed by Paclitaxel with or without Bevacizumab in Patients with Lymph Node Positive and High Risk Lymph Node Negative Breast Cancer.

What is bevacizumab (Avastin) and why add it to chemotherapy?

Tumors need an increased blood supply in order to grow. The process of growing new blood vessels is called angiogenesis and tumors have the ability to trigger this process. Bevacizumab (B) can block signals to the blood vessels that tell them to grow. Research has shown that adding an anti-angiogenesis drug like bevacizumab to chemotherapy resulted in a 50% reduction in the risk of cancer progression in women with metastatic breast cancer. Researchers believe that adding bevacizumab to chemotherapy for newly diagnosed women may also be successful in treating breast cancer. Research has shown that Adriamycin and Cytoxan followed by Taxol (AC ->T) is one of the most effective chemotherapies used in the adjuvant setting (for newly diagnosed women and men).

What is the purpose of this study?

This study of 4950 women and men has three objectives:

- To determine if adding bevacizumab (B) to doxorubicin/cyclophosphamide followed by paclitaxel (AC>T) will increase how long patients live without a recurrence of breast cancer (disease free survival)
- To compare short-term (20-24 weeks) versus long-term (50-54 weeks) bevacizumab therapy
- To collect biologic samples including primary tumor blocks, genomic DNA and serum at study entry for subsequent studies.

Who is eligible?

Women and men with newly diagnosed lymph node positive or high risk lymph node negative breast cancer who:

- Are not HER2 positive
- Have had a mastectomy or breast conservation surgery and removal of the lymph nodes.
- Have no evidence of breast cancer after surgery – clear margins
- Do not have a history of clinically significant heart disease
- Are not pregnant

Why use a placebo?

If you or your doctor knew that you were receiving bevacizumab, it might influence the results of this study. To avoid this problem, patients assigned to Arm A will receive a placebo infusion. A placebo is an inactive substance that has no effect on the body. You will be informed of your treatment group at the beginning of your last cycle of paclitaxel (week 18 or week 22).

There are two different dosing schedules of AC for this study called, “classical” and “dose dense”, which one you receive will be decided by you and your doctor

How is the study designed?
If you go on the classical schedule of AC you will be randomized into one of the following three study groups:

Group A –

- AC plus placebo every 3 weeks for 4 treatments followed by
- T weekly for 12 treatments plus placebo every 3 weeks for 4 treatments

Group B –

- BAC every 3 weeks for 4 treatments followed by
- T weekly for 12 treatments **+** B every 3 weeks for 4 treatments

Group C –

- BAC every 3 weeks for 4 treatments followed by
- T weekly for 12 treatments **+** B every 3 weeks for 4 treatments followed by

If registered onto Group D:

- B every 3 weeks for 10 treatments

How is the study designed?
If you go on the dose dense schedule of AC you will be randomized into one of the following three study groups:

Group A –

- AC plus placebo every 2 weeks for 4 treatments followed by
- T weekly for 12 treatments **+** placebo every 3 weeks for 4 treatments

Group B –

- BAC every 14 days for 4 treatments followed by
- T weekly for 12 treatments **+** B every 3 weeks for 4 treatments

Group C –

- BAC every 2 weeks for 4 treatments followed by
- T weekly for 12 treatments **+** B every 3 weeks for 4 treatments followed by

If registered onto Group D:

- B every 3 weeks for 10 treatments

A Double-Blind Phase III Trial of Doxorubicin and Cyclophosphamide followed by Paclitaxel with Bevacizumab or Placebo in Patients with Lymph Node Positive and High Risk Lymph Node Negative Breast Cancer

Appendix VII

Cancer Trials Support Unit (CTSU) Participant Procedures

REGISTRATION/RANDOMIZATION

Prior to the recruitment of a patient for this study, investigators must be registered members of the CTSU. Each investigator must have an NCI investigator number and must maintain an “active” investigator registration status through the annual submission of a complete investigator registration packet (FDA Form 1572 with original signature, current CV, Supplemental Investigator Data Form with signature, and Financial Disclosure Form with original signature) to the Pharmaceutical Management Branch, CTEP, DCTD, NCI. These forms are available on the CTSU registered member Web site or by calling the PMB at 240-276-6575 Monday through Friday between 8:30 a.m. and 4:30 p.m. Eastern time.

Rev.1/15

Each CTSU investigator or group of investigators at a clinical site must obtain IRB approval for this protocol and submit IRB approval and supporting documentation to the CTSU Regulatory Office before they can enroll patients. Study centers can check the status of their registration packets by querying the Regulatory Support System (RSS) site registration status page of the CTSU member web site at http://members.ctsu.org

All forms and documents associated with this study can be downloaded from the E5103 Web page on the CTSU registered member Web site (https://members.ctsu.org). Patients can be registered only after pre-treatment evaluation is complete, all eligibility criteria have been met, and the study site is listed as ‘approved’ in the CTSU RSS.

Requirements for E5103 site registration:

- CTSU IRB Certification
- CTSU IRB/Regulatory Approval Transmittal Sheet

Prestudy requirements for patient enrollment on E5103:

- Patient must meet all inclusion criteria, and no exclusion criteria should apply
- Patient has signed and dated all applicable consents and authorization forms
- All baseline laboratory tests and prestudy evaluations performed within the time period specified in the protocol.

CTSU Procedures for Patient Enrollment

Step 1: Randomization to Arm A, B or C

1. Contact the CTSU Patient Registration Office by calling 1-888-462-3009 between 9:00 AM and 5:30 PM Eastern Time, Monday – Friday. Leave a voicemail to alert the CTSU Patient Registrar that an enrollment is forthcoming. For immediate registration needs, e.g. within one hour, call the registrar cell phone at 1-301-704-2376.
2. Complete the following forms:

- CTSU Patient Enrollment Transmittal Form
- E5103 Eligibility Checklist

3. Fax these forms to the CTSU Patient Registrar at 1-888-691-8039 between the hours of 9:00 AM and 5:30 PM, Monday – Friday, Eastern Time (excluding holidays); however, please be aware that registrations received after 5:00 PM will be processed the next day. The CTSU registrar will check the investigator and site information to ensure that all regulatory requirements have been met. The registrar will also check that forms are complete and will follow-up with the site to resolve any discrepancies.
4. Once investigator eligibility is confirmed and enrollment documents are completed, the CTSU registrar will contact the ECOG-ACRIN to register the patient and to obtain a unique patient ID (to be used on all future forms and correspondence). The CTSU registrar will access the ECOG-ACRIN's on-line registration system, to obtain and assignment of a treatment arm and a unique patient ID (to be used on all future forms and correspondence). The CTSU registrar will confirm registration by fax.
   - Treatment should start within ten working days after randomization.
   - Rev. 12/07, 5/08 All patients and treating physicians will be blinded as to assigned treatment group through Cycle 7. All patients and physicians will be unblinded on Day 1 of Cycle 8. If the patient is found to have been treated on Arm A or Arm B, then the patient will then discontinue protocol treatment following the completion of cycle 8. If the patient is found to have been treated on Arm C, then the patient may be registered to Arm D for treatment with Bevacizumab for cycles 9-18. To unblind patients on Cycle 8, Day 1, follow the instructions outlined in Appendix XI. CTSU sites should follow the instructions pertaining to the web unblinding program and access the program at https://webreg.ecog.org/webunblind. CTSU sites should enter their CTEP ID and password to enter the system.

Step 2: Re-registration to Arm D for Unblinded Patients on Arm C

1. Contact the CTSU Patient Registration Office by calling 1-888-462-3009. Leave a voicemail to alert the CTSU Patient Registrar that an enrollment is forthcoming. For immediate registration needs, e.g. within one hour, call the registrar cell phone at 1-301-704-2376.
2. Complete the following forms:
   - Rev. 12/07 E5103 Step 2 Eligibility Checklist
   - CTSU Patient Enrollment Transmittal Form

Rev. 12/07

3. Fax these forms to the CTSU Patient Registrar at 1-888-691-8039 between the hours of 8:00 a.m. and 8:00 p.m., Mon-Fri, Eastern Time (excluding holidays). The CTSU registrar will check the investigator and site information to ensure that all regulatory requirements have been met. The registrar will also check that forms are complete and follow-up with the site to resolve any discrepancies. 4. The CTSU registrar will access the ECOG-ACRIN's on-line registration system to register the patient. The CTSU registrar will confirm registration by fax.

DATA SUBMISSION AND RECONCILIATION

1. All case report forms (CRFs) and transmittals associated with this study must be downloaded from the E5103 Web page located on the CTSU registered member

Web site (https://members.ctsu.org). Sites must use the current form versions and adhere to the instructions and submission schedule outlined in the protocol.

2. Submit all completed CRFs (with the exception of patient enrollment forms), clinical reports, and transmittals directly to the ECOG-ACRIN [refer to contacts table] unless an alternate location is specified in the protocol. Do not send study data to the CTSU.

3. The ECOG-ACRIN Operations Office - Boston will mail query notices and delinquency reports directly to the site for reconciliation. Please send query responses and delinquent data to the ECOG-ACRIN Operations Office - Boston and do not copy the CTSU Data Operations. Each site should have a designated CTSU Administrator and Data Administrator and **must keep their CTEP AMS account contact information current.** This will ensure timely communication between the clinical site and the ECOG-ACRIN Operations Office - Boston.

SPECIAL MATERIALS OR SUBSTUDIES

Rev. 2/13

1. Specimen collection for banking for future laboratory research studies (Protocol section 10.0 and Appendix II) or the EL112LAB Ancillary Substudy (Appendix XV)

Rev. 2/13

- Participation in the banking aspects or EL112LAB is optional and requires patient consent.
- Collect, prepare, and submit specimens as outlined in the protocol.
- Do not send specimens, supporting clinical reports, or transmittals to the CTSU.
- All specimens submitted for this study must be entered and tracked using the ECOG-ACRIN Sample Tracking System. Upon registering new patients, you can expect to receive an automatic email with instructions for logging into the system and shipping samples. You can also access the Tracking System from the CTSU Member Web Site. Go to the E5103 protocol page and click on the link provided under the Case Report Forms header.

Rev. 4/08

Kits for sample collection and shipment are available for sites in the United States and Canada. Follow the instructions in Appendix XIV.

SERIOUS ADVERSE EVENT (AE) REPORTING (SECTION 5.3)

1. CTSU sites must comply with the expectations of their local Institutional Review Board (IRB) regarding documentation and submission of adverse events. Local IRBs must be informed of all reportable serious adverse reactions.
2. CTSU sites will assess and report adverse events according to the guidelines and timelines specified in the protocol. You may navigate to the CTEP Adverse Event Expedited Report System (CTEP-AERS) from either the Adverse Events tab of the CTSU member homepage (https://members.ctsu.org) or by selecting Adverse Event Reporting Forms from the document center drop down list on the E5103 Web page.
3. Do not send adverse event reports to the CTSU.
4. Secondary AML/MDS/ALL reporting: Report occurrence of secondary AML, MDS, or ALL via the NCI/CTEP AML-MDS Report Form in lieu of CTEP-AERS. Submit the completed form and supporting documentation as outlined in the protocol.

DRUG PROCUREMENT (SECTION 8.0)

Investigational IND agents: Bevacizumab (Note that the PMB will ship drug only to the shipping address specified by the CTSU investigator on their FDA form 1572.)

Commercial Agents: Doxorubicin, Cyclophosphamide, Paclitaxel

1. Information on drug formulation, procurement, storage and accountability, administration, and potential toxicities are outlined in section 8.0 of the protocol.
2. You may navigate to the drug forms by selecting Pharmacy Forms from the document center drop down list on the E5103 Web page.

REGULATORY AND MONITORING

Study Audit

To assure compliance with Federal regulatory requirements [CFR 21 parts 50, 54, 56, 312, 314 and HHS 45 CFR 46] and National Cancer Institute (NCI)/ Cancer Therapy Evaluation Program (CTEP) Clinical Trials Monitoring Branch (CTMB) guidelines for the conduct of clinical trials and study data validity, all protocols approved by NCI/CTEP that have patient enrollment through the CTSU are subject to audit.

Responsibility for assignment of the audit will be determined by the site's primary affiliation with a Cooperative Group or CTSU. For Group-aligned sites, the audit of a patient registered through CTSU will become the responsibility of the Group receiving credit for the enrollment. For CTSU Independent Clinical Research Sites (CICRS), the CTSU will coordinate the entire audit process.

For patients enrolled through the CTSU, you may request the accrual be credited to any Group for which you have an affiliation provided that Group has an active clinical trials program for the primary disease type being addressed by the protocol. (e.g., NSABP members may only request credit for protocols pertaining to breast or colorectal cancers). Registrations to protocols for other disease sites may still take place through CTSU without receiving credit for your NSABP activities. Per capita reimbursement will be issued directly from CTSU.

Details on audit evaluation components, site selection, patient case selection, materials to be reviewed, site preparation, on-site procedures for review and assessment, and results reporting and follow-up are available for download from the CTSU Operations Manual located on the CTSU Member Web site.

Health Insurance Portability and Accountability Act of 1996 (HIPAA)

The HIPAA Privacy Rule establishes the conditions under which protected health information may be used or disclosed by covered entities for research purposes. Research is defined in the Privacy Rule referenced in HHS 45 CFR 164.501. Templated language addressing NCI-U.S. HIPAA guidelines are provided in the HIPAA Authorization Form located on the CTSU website.

The HIPAA Privacy Rule does not affect participants from outside the United States. Authorization to release Protected Health Information is NOT required from patients enrolled in clinical trials at non-US sites.

Clinical Data Update System (CDUS) Monitoring

This study will be monitored by the Clinical Data Update System (CDUS) Version 3.0. Cumulative CDUS data will be submitted quarterly to CTEP by electronic means. The sponsoring Group fulfills this reporting obligation by electronically transmitting to CTEP the CDUS data collected from the study-specific case report forms.

A Double-Blind Phase III Trial of Doxorubicin and Cyclophosphamide followed by Paclitaxel with Bevacizumab or Placebo in Patients with Lymph Node Positive and High Risk Lymph Node Negative Breast Cancer

Appendix VIII

Rev. 2/13

E5103 Shipment Notification Form – REMOVED

Rev. 12/07
Rev. 1/15

All samples submitted from patients participating in E5103 must be logged into the online ECOG-ACRIN Sample Tracking System (see Section 10.4). In the event that STS is not available at time of sample shipment use the Generic Specimen Submission Form (#2981) found in the forms packet or Appendix II. When STS is available, shipments must be logged retroactively into the system, indicating the actual dates of collection and shipment.

A Double-Blind Phase III Trial of Doxorubicin and Cyclophosphamide followed by Paclitaxel with Bevacizumab or Placebo in Patients with Lymph Node Positive and High Risk Lymph Node Negative Breast Cancer

Appendix IX

Cooperative Research and Development Agreement (CRADA)

The agent(s) supplied by CTEP, DCTD, NCI used in this protocol is/are provided to the NCI under a Collaborative Agreement (CRADA, CTA, CSA) between the Pharmaceutical Company(ies) (hereinafter referred to as 'Collaborator(s)') and the NCI Division of Cancer Treatment and Diagnosis. Therefore, the following obligations/guidelines, in addition to the provisions in the Intellectual Property Option to Collaborator
Rev. 1/15 (http://ctep.cancer.gov/industryCollaborations2/intellectual_property.htm) contained within the terms of award, apply to the use of the Agent(s) in this study:

1. Agent(s) may not be used for any purpose outside the scope of this protocol, nor can Agent(s) be transferred or licensed to any party not participating in the clinical study. Collaborator(s) data for Agent(s) are confidential and proprietary to Collaborator(s) and shall be maintained as such by the investigators. The protocol documents for studies utilizing investigational Agents contain confidential information and should not be shared or distributed without the permission of the NCI. If a copy of this protocol is requested by a patient or patient's family member participating on the study, the individual should sign a confidentiality agreement. A suitable model agreement can be downloaded from: http://ctep.cancer.gov.

2. For a clinical protocol where there is an investigational Agent used in combination with (an)other investigational Agent(s), each the subject of different collaborative agreements , the access to and use of data by each Collaborator shall be as follows (data pertaining to such combination use shall hereinafter be referred to as "Multi-Party Data."):

    a. NCI will provide all Collaborators with prior written notice regarding the existence and nature of any agreements governing their collaboration with NIH, the design of the proposed combination protocol, and the existence of any obligations that would tend to restrict NCI's participation in the proposed combination protocol.

    b. Each Collaborator shall agree to permit use of the Multi-Party Data from the clinical trial by any other Collaborator solely to the extent necessary to allow said other Collaborator to develop, obtain regulatory approval or commercialize its own investigational Agent.

    c. Any Collaborator having the right to use the Multi-Party Data from these trials must agree in writing prior to the commencement of the trials that it will use the Multi-Party Data solely for development, regulatory approval, and commercialization of its own investigational Agent.

3. Clinical Trial Data and Results and Raw Data developed under a Collaborative Agreement will be made available exclusively to Collaborator(s), the NCI, and the FDA, as appropriate and unless additional disclosure is required by law or court order as described in the IP Option to Collaborator (http://ctep.cancer.gov/industryCollaborations2/intellectual_property.htm). Additionally, all Clinical Data and Results and Raw Data will be collected , used and disclosed

consistent with all applicable federal statutes and regulations for the protection of human subjects, including, if applicable, the *Standards for Privacy of Individually Identifiable Health Information* set forth in 45 C.F.R. Part 164.

4. When a Collaborator wishes to initiate a data request, the request should first be sent to the NCI, who will then notify the appropriate investigators (Group Chair for Cooperative Group studies, or PI for other studies) of Collaborator's wish to contact them.

5. Any data provided to Collaborator(s) for Phase 3 studies must be in accordance with the guidelines and policies of the responsible Data Monitoring Committee (DMC), if there is a DMC for this clinical trial.

6. Any manuscripts reporting the results of this clinical trial must be provided to CTEP by the Group office for Cooperative Group studies or by the principal investigator for non-Cooperative Group studies for immediate delivery to Collaborator(s) for advisory review and comment prior to submission for publication. Collaborator(s) will have 30 days from the date of receipt for review. Collaborator shall have the right to request that publication be delayed for up to an additional 30 days in order to ensure that Collaborator's confidential and proprietary data, in addition to Collaborator(s)'s intellectual property rights, are protected. Copies of abstracts must be provided to CTEP for forwarding to Collaborator(s) for courtesy review as soon as possible and preferably at least three (3) days prior to submission, but in any case, prior to presentation at the meeting or publication in the proceedings. Press releases and other media presentations must also be forwarded to CTEP prior to release. Copies of any manuscript, abstract and/or press release/ media presentation should be sent to:

Rev. 1/15 Email: ncicteppubs@mail.nih.gov

7. The Regulatory Affairs Branch will then distribute them to Collaborator(s). No publication, manuscript or other form of public disclosure shall contain any of Collaborator's confidential/ proprietary information.

A Double-Blind Phase III Trial of Doxorubicin and Cyclophosphamide followed by Paclitaxel with Bevacizumab or Placebo in Patients with Lymph Node Positive and High Risk Lymph Node Negative Breast Cancer

Appendix X

NYHA Classification and Peripheral Vascular Disease Grading

New York Heart Association (NYHA) Classification

Class I: patients with no limitation of activities; they suffer no symptoms from ordinary activities.

Class II: patients with slight, mild limitation of activity; they are comfortable with rest or with mild exertion.

Class III: patients with marked limitation of activity; they are comfortable only at rest.

Class IV: patients who should be at complete rest, confined to bed or chair; any physical activity brings on discomfort and symptoms occur at rest.

Peripheral Vascular Disease Grading (Fontaine)

Grade I: Asymptomatic decreased distal pulses (Ankle-Brachial Index < 0.9)

Grade II: Intermittent claudication

Grade III: Daily rest pain

Grade IV: Focal tissue necrosis (Ankle-Brachial Index < 0.3)

A Double-Blind Phase III Trial of Doxorubicin and Cyclophosphamide followed by Paclitaxel with Bevacizumab or Placebo in Patients with Lymph Node Positive and High Risk Lymph Node Negative Breast Cancer

Appendix XI

E5103 Cycle 8 Unblinding Instructions

All patients and treating physicians will be blinded to the assigned treatment group through cycle 7 of step 1. Following the completion of Cycle 7 treatment, all patients are to be unblinded on cycle 8, day 1 of treatment. Clinical research associates (CRAs), nurses, or physicians should follow the instructions below for the E5103 Web Unblind Program to unblind the patients registered at their institution

NOTE: The E5103 Web Unblind Program will not unblind patients prior to Cycle 8, Day 1 of their treatment.

NOTE: The E5103 Web Unblind Program should not be used for cases involving emergency unblinding. For information regarding emergency unblinding, please see Section 4.8 of the protocol.

NOTE: The CRA, nurse, or physician must be affiliated with the institution that registered the patient to step 1 of the protocol.

E5103 Web Unblinding Program Instructions:

1. Access the Web Unblind program at: https://webreg.ecog.org/webunblind
2. Enter your username and password for the ECOG web site.
3. Enter the protocol (Prot), patient sequence number (Case), and step (step will always be “1”) in the appropriate fields.
4. Type a comment in the *Reason for Unblinding* field:
    a. Enter your name, followed by “Cycle 8, day 1 protocol unblinding.”

       For example, “Sally Smith-Cycle 8, day 1 protocol unblinding.”
5. Click **Unblind Treatment.**

   The screen is now updated to include the patient’s step 1 treatment assignment, as shown in the following example:

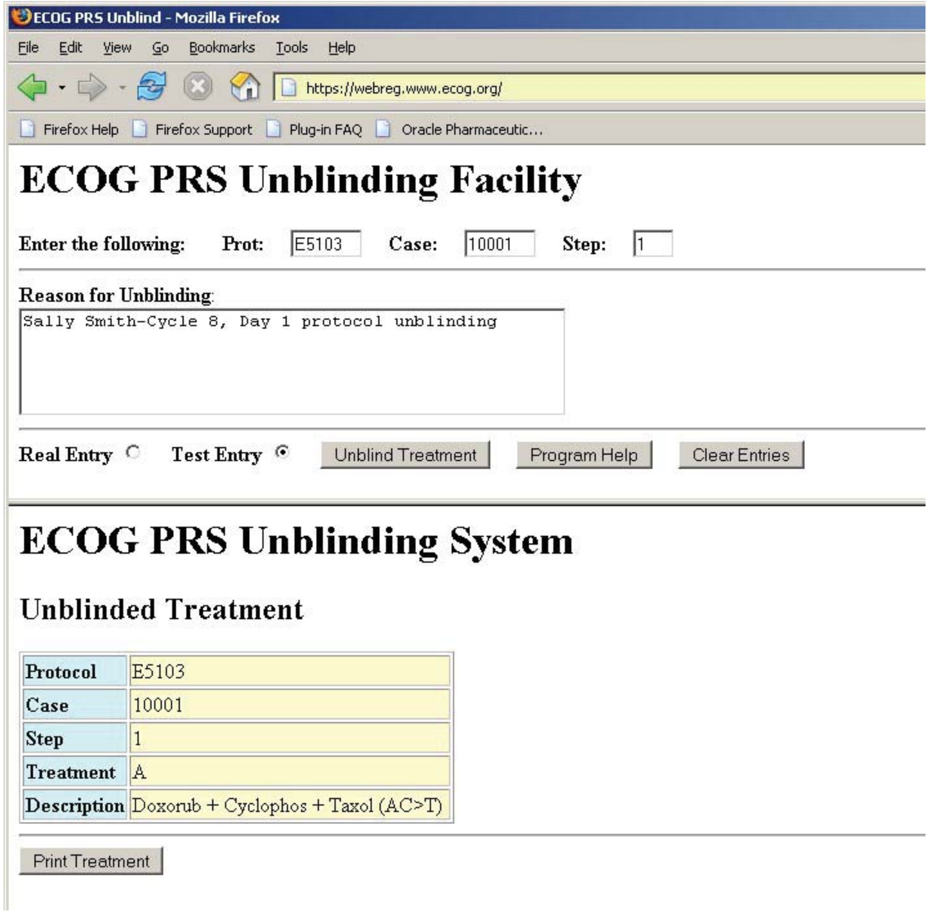

If the *Treatment* field in the Web Unblind program indicates that the patient received arm A or B, the patient should complete cycle 8 of treatment on Step 1 and then be followed per protocol. ***These patients are not eligible to register to step 2 of the protocol.***

If the *Treatment* field in the Web Unblind program indicates that the patient received arm C, the patient should complete cycle 8 of treatment on Step 1 and then register to step 2.

If you have any questions regarding the E5103 Web Unblind Program, please contact a member of the ECOG-ACRIN Drug Team at (617) 632-3610 or ecog.drugorder@jimmy.harvard.edu.

A Double-Blind Phase III Trial of Doxorubicin and Cyclophosphamide followed by Paclitaxel with Bevacizumab or Placebo in Patients with Lymph Node Positive and High Risk Lymph Node Negative Breast Cancer

Appendix XII

Substitute W-9 Tax Form

Instructions

1. This form is a substitute to the Internal Revenue Service W-9 Tax Form. In addition to capturing all of the information required by the IRS, it also collects other information that is needed for our records. Questions regarding this form should be directed to the Pharmaceutical Liaison at the ECOG-ACRIN Operations Office - Boston 617-632-3610. Please note that only US-based institutions can use this form.
2. Complete all requested information and sign the substitute W-9 form. The original copy should be submitted to the ECOG-ACRIN Operations Office - Boston as soon as possible.

Please provide the legal name and address of the organization associated with the Federal Tax Identification Number listed in this section. (Generally, the corporate headquarters address of the university, hospital, or business should be provided. Genentech will use this information for income reporting to the IRS and your organization).

Legal Name:____________________________________

Corporate Address: ____________________________________

____

Rev. 5/08 **Circle Group Affiliation: ACOSOG, CALGB, CTSU, ECOG-ACRIN, GOG, NCCTG, NCIC, NSABP, RTOG, SWOG**

City:________________ **State:**__________ **ZipCode:**__________

NCI CTEP ID:____________________

Federal Tax Identification Number:______________ **Phone Number:**______________

Please identify the organization's preferred payment address. Genentech will use this information for mailing checks to the organization. Please note that while Genentech can submit payment to an alternate address, it cannot make checks payable to a different organizational name or to a third party.

Payment Address:____________________________________

City:________________ **State:**__________ **ZipCode:**__________

Phone Number: __________ **Fax Number:**__________ **E-mail:** ____________

Is this payment address affiliated with the Federal Tax ID listed above?:________________

Please identify whether the organization has a special status as defined by the following criteria:
(Select all that apply)

Minority Business Enterprise (at least 51% minority-owned and managed business)__________
Woman's Business Enterprise (at least 51% woman-owned and managed business)__________
Small Disadvantaged Business (as certified by the SBA) __________
Veteran Business Enterprise (at least 51% veteran-owned business) __________
Historically Underutilized Small Business (as certified by the SBA) __________
None of the Above __________

Under penalties of perjury, I certify that all of the information provided above is correct and that my organization is not subject to back-up withholding.

Printed Name:____________________ **Title:**__________________

Signature:____________________ **Date:**__________________

A Double-Blind Phase III Trial of Doxorubicin and Cyclophosphamide followed by Paclitaxel with Bevacizumab or Placebo in Patients with Lymph Node Positive and High Risk Lymph Node Negative Breast Cancer

Appendix XIII

Important Tips for this FDA Registration Trial

ECOG-ACRIN, NCI, and Genentech have entered into a collaborative agreement on E5103, and results from this trial are intended to be reported to the Food and Drug Administration (FDA) as a well-controlled study in support of a new indication for the use of Bevacizumab as adjuvant therapy for patients with lymph node positive and high risk node negative breast cancer. For this reason, ECOG-ACRIN developed E5103 as an FDA registration protocol, which means that this protocol involves several special handling and reporting procedures, some of which are described below and others will be detailed in an ECOG website posting, and via the CTSU website for other Groups and CICRS sites that activate E5103.

1. **Alpha Oncology Data Sweeps**: ECOG-ACRIN has contracted with Alpha Oncology, a contract research organization (CRO), to conduct data sweeps. The goal of these data sweeps is to assist institutions with timely data submission. Alpha Oncology will utilize phone requests, email notifications, and site visits of high-accruing institutions to collect current survival information, data clarification form (DCF) responses, and any outstanding data. The objective of these sweeps is to collect data; they are not designed as an audit, nor are they intended to generate corrections to previously submitted data.
2. **Case Report Forms and Data Submission**: The FDA has requested that additional data points be collected for this protocol. Therefore, additional reimbursement will be provided, as outlined below. The E5103 forms submission schedule has been posted with the protocol on the ECOG web site (www.ecog.org). The forms submission schedule provides guidance about when each of the case report forms (CRFs) is due. Institutions should adhere to the data submission timelines provided in this schedule.
3. **Institution Reimbursement**: Because E5103 will involve additional workload at the institutions, Genentech has agreed to provide additional institution reimbursement to help offset the associated costs. The reimbursement amount is posted on the Reimbursement page of the ECOG web site at (http://www.ecog.org/ecoginst/tools/ecog_reimbursements.html) and on the CTSU website. Reimbursements for non-ECOG-ACRIN institutions will be paid to the applicable cooperative group, which will be responsible for forwarding payment to their member institutions. Eligibility for and receipt of this additional reimbursement will be based on timely data submission.

Follow-up with Institutions: E5103 is expected to reach a full accrual of 4950 patients within approximately 25 months, with a follow-up period of 6.2 years. Therefore, the final analysis of the protocol is expected to take place approximately 6.5 years after activation. These time frames are only approximations and may be altered based on the actual accrual rate and the rate of data events generated. To assist Genentech with their regulatory filing, ECOG-ACRIN will be providing a database transfer to Genentech after the study is released by ECOG-ACRIN's Data Monitoring Committee (DMC). At that time, Genentech will review the data and may have questions for the institutions on certain data points. These questions may result in additional data clarification forms (DCFs) being sent to the institutions by the ECOG-ACRIN E5103 data management team. Therefore, institutions may be expected to answer DCFs a few years after the actual data event occurred.

A Double-Blind Phase III Trial of Doxorubicin and Cyclophosphamide followed by Paclitaxel with Bevacizumab or Placebo in Patients with Lymph Node Positive and High Risk Lymph Node Negative Breast Cancer

Rev. 12/07, 10/13
Rev. Add14

Appendix XIV

E5103 and EL112LAB Collection and Shipping Kit Order Form

As of April 12, 2016 Specimen Collection/Shipping Kits are provided by CENETRON CENTRAL LABORATORIES and are ordered ONLINE at www.cenetron.com

Kits may be ordered within one month prior to visit, including that for patients who are potentially eligible to E5103, Step 3 but not yet consented. EL112LAB1 kits are not protocol or patient specific and can be interchanged between patients and trial. Protocol # must be provided for tracking purposes.

Please complete the online form completely, including the valid ECOG-ACRIN protocol number, ECOG-ACRIN patient case number, and complete shipping address. If information is missing the kit processing will be delayed.

Ordering process:

1. Proceed to www.cenetron.com. Click the "Order Kits" button at the top right.
2. Complete the online form as follows
   - Sponsor (REQUIRED): ECOG-ACRIN
   - Contact Name (REQUIRED): Name of the site's kit contact. Should match the name of the individual provided in OPEN as the kit contact
   - Protocol Number (REQUIRED): E5103 (or PACT1)
   - Phone Number (REQUIRED): Phone number of the kit contact. Please insure that this is a number that can be reached from an external caller.
   - Site Number (REQUIRED): Institution's NCI site ID
   - FAX Number: Fax number of the kit contact
   - Investigator: Last name of the kit contact is adequate
   - Email (REQUIRED): The email of the site's kit contact. Must be entered twice to confirm
   - Date Supplies Needed (REQUIRED): Add 3 **business** days or more to order date. E.g. if ordering on 2/5/2016, indicate 2/10/2016 to accommodate the weekend. Reminder that holidays must also be considered in this timeline.
   - KIT NAME (required): Complete with the appropriate kit
     - EL112LAB Step 3 BASELINE*

       *NOTE: This kit is for E5103 only. The baseline kit may lack the Immunex CellSAVE tube. Please substitute using an 8.5–10mL EDTA vacutainer from institutional supplies and ship First Overnight to Fox Chase per protocol.
     - EL112LAB Follow-up Kit

- Quantity: 1
- Comments: Provide E5103 (or PACT1) Case ID and full shipping address
  - “Patient caseID =” #####
  - "Ship kit to" Name of the individual to whom the kit is shipped.
  - Full street address, town, state and zip code
- Answer the security question

Questions regarding kits can be directed to projectmanagement@cenetron.com or call the Cenetron clinical trials group at 512-439-2000

A Double-Blind Phase III Trial of Doxorubicin and Cyclophosphamide followed by Paclitaxel with Bevacizumab or Placebo in Patients with Lymph Node Positive and High Risk Lymph Node Negative Breast Cancer

Rev. Add14

Appendix XV
Ancillary Study EL112LAB

North American Breast Cancer Groups Biospecimen Bank for Determinants of Late Relapse in Operable Breast Cancer

Study PIs: Joseph Sparano, MD and

Patients registered to treatment on E5103 or PACT1 who meet the criteria outlined in Section XV.6 are eligible to participate in this ancillary study. Patients must provide written signed consent, additional to that from participation in the treatment trial, to participate in this ancillary study.

Guidelines for enrollment and submission of data and specimens are outlined below in Section XV.6. A model consent for this ancillary study is provided as a sub-appendix (Appendix I-A) to the model consent of the respective treatment trial.

XV.1 Hypothesis and Specific Aims

Late relapse (defined as occurring 5 or more years after diagnosis) accounts for up to one-half of all breast cancer recurrences, and is difficult to study because of the lack of adequate biospecimens linked to clinical data with sufficiently long follow-up. In addition, recurrence may be driven by dynamic rather than static host factors that may wax or wane with time. ***We propose that our current paradigm of assessing and counseling solely at diagnosis may need to be reconsidered to include a second evaluation point 5 or more years after diagnosis when patients continue to be at risk for recurrence, and needs to include an evaluation of both tumor and host-related factors in the context of competing risks. We hypothesize that tumor and host-related factors contributing to recurrence that may be identified and potentially modifiable via pharmacologic or nutritional or other lifestyle interventions***.

Our specific aims include:

1. To create a biospecimen repository including plasma, serum and CellSearch™ cassettes containing circulating tumor cells (CTC) for evaluating determinants of late relapse, including ***candidate biomarkers*** reflecting occult tumor burden (e.g., CTCs and plasma tumor DNA) and host factors (e.g., estrogen, insulin-IGF axis, inflammation, etc).
2. To create a biorepository of metastatic tumor samples in patients who have had a late relapse.
3. To determine body mass index (BMI) and comorbidity burden in patients with operable breast cancer five or more years after diagnosis.
4. To determine whether there is a relationship between late relapse and BMI at diagnosis and at 5 years after diagnosis, and whether BMI-associated inflammatory and/or metabolic biomarkers are associated with early and late recurrence.

XV.2 Background and Significance

Late relapse occurring after 5 years or more after a diagnosis of operable breast cancer is a major clinical problem accounting for up to one-half of all relapses in ER-positive disease, but also may occur unpredictably in other breast cancer subtypes.[1,2] For example, when evaluating patterns of recurrence in 4950 eligible patients enrolled in the E1199 trial who received adjuvant chemotherapy (plus endocrine therapy if ER-positive)[3], the annual hazard rate (HR) of recurrence within the first 5 years of diagnosis was about 3-fold higher for patients with triple negative breast cancer (TNBC) and 2-fold higher for HER2/neu-positive (HER2+) breast cancer compared with ER- and/or PR positive, HER2-negative disease (ER+/PR+), but was higher for ER+/PR+ disease compared with other subtypes beyond 5 years and remained consistent over time (**Figure 1A, left**).[4] In addition, host factors such as body mass index (BMI) at diagnosis may contribute to late relapse, especially in ER+/PR+ disease (**Figure 1B, right**). Two prior reports have found a similar association.[5] [6]

Figure 1A

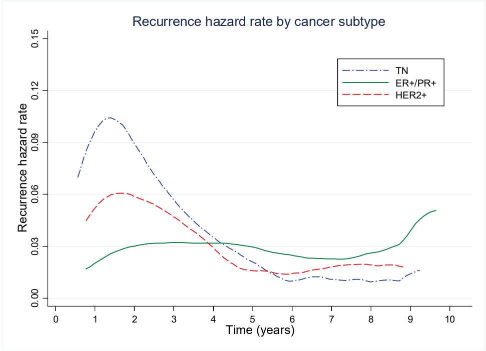

Figure 1B

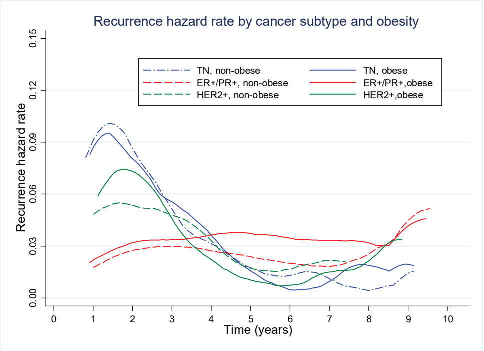

Figure 1A (left): Annual hazard rate (HR) for recurrence for patients with operable breast cancer treated with adjuvant sequential doxorubicin/cyclophosphamide-taxane therapy in trial E1199, including patients with ER- and/or PR-positive, HER2 negative disease (ER+PR+), triple negative disease (TN) and HER2/neu positive disease (since there was limited information beyond 8-9 years, the confidence intervals are wide at the tails of these curves)
Figure 1B (right): HR of recurrence in obese (BMI >/= 30 kg/m2) vs. non-obese patients at diagnosis**.**

There are no clinical or pathologic features predictive of late relapse; gene expression assays predict earlier recurrences.[7] [8] Although extended adjuvant therapy with an aromatase inhibitor given for up to 5 years after 2-5 year course of tamoxifen therapy has been shown to reduce the risk of recurrence in ER-positive disease, the absolute benefits are low, resulting in many patients receiving unnecessary therapy.[9]

XV.3 Candidate Biomarkers for Late Recurrence: Plasma Tumor DNA, Insulin/IGF Axis, and Inflammation

Plasma Tumor DNA and Circulating Tumor Cells (CTCs). Several assays are available that may detect occult tumor burden, but their clinical utility has not been established. Detection of circulating tumor DNA in the blood of cancer-free patients is one such assay, although the sensitivity, specificity, and positive/negative predictive value for predicting recurrence is currently unknown.**[10]** Enumeration of CTCs in the blood (≥ 1 CTC in 7.5 ml blood) has also shown to be associated with recurrence when

detected in patients with operable breast cancer who have not yet had surgery**[11]**, but has not been evaluated as a predictor of recurrence in patients who are cancer free for at least 5 years after surgery. ***Measurement of both plasma tumor DNA and CTC in the same patients at the same time offers an excellent opportunity to evaluate and directly compare these two technologies.*** Although the CTC enumeration must be done in real time, the results will not be provided to the clinician or patient for clinical decision making. CellSearch™ cassettes containing the CTC will be dried and stored for future analysis. Plasma will be evaluated at a future date when the technology has been optimized, and when the sample collection has been completed and there is sufficient clinical follow-up and events to permit an analysis.

Insulin-IGF Axis. As described above, obesity at diagnosis is associated with late relapse in ER+PR+ disease. Obesity is associated with hyperinsulinemia, and higher fasting insulin levels have been associated with increased breast cancer risk[12] and breast cancer recurrence.[13-15] The relation of obesity with insulin resistance, metabolic syndrome, and cardiovascular disease has been shown to vary. The concept of the “healthy obese” – a subset of patients who demonstrate few pernicious effects related to their obesity – has been described and increasingly studied.[16] This could apply as well to cancer risk in obese patients. Thus, risk stratification by relevant “pernicious biomarkers” could potentially be important in identifying which obese patients are at increased risk of recurrence and would therefore benefit from specific interventions. Insulin shares substantial amino acid sequence homology, downstream signaling pathways, and mitogenic/anti-apoptotic activity with insulin-like growth factor (IGF)-1, a peptide hormone that mediates many of the growth effects of growth hormone. Insulin receptors are expressed at high levels in breast cancer cell lines[17] and human breast cancer specimens.[18] Insulin decreases levels of sex hormone binding globulin (thereby increasing free estradiol)[19], upregulates androgen secretion by the ovaries[20], increases HR expression and binding capacity[21], and induces proliferation in normal and dysplastic breast epithelial cells. [21,22] Moreover, higher expression of activated IGF-1/insulin receptors has been associated with higher risk of recurrence[23], and IGF signaling has been associated with resistance to paclitaxel.[24] Elevated HOMA (homeostasis model assessment) scores and low levels of adiponectin (both associated with obesity) were associated with increased breast cancer mortality in women enrolled in the Health, Eating, Activity, and Lifestyle (HEAL) Study, a prospective cohort study of women with stage I-IIIA breast cancer.[25] The HOMA score is a method for assessing ß-cell function and insulin resistance ([insulin μ/mL X glucose mmol/L]/22.5).[26] In addition, chronic hypergylcemia (reflected by an elevated Hemoglobin A1C level) has been associated with a higher risk of breast cancer recurrence and death.[27] Finally, a systematic review and meta-analysis examining the effect of pre-existing diabetes on breast cancer-related outcomes found that diabetics had a greater risk of death and present with more advanced stage disease. [28] The IGF-axis also includes and IGF-2, a related growth factor, and six IGF binding proteins (IGFBPs) which have both IGF-dependent and independent effects. In addition to their levels in circulation, IGF-axis proteins are produced locally in tissues with autocrine and paracrine activity. HR-positive breast cancers exhibit significantly higher gene expression of the IGF pathway.[29] Widespread gene expression alterations have been described in breast tumors from obese patients as compared to other tumors, which resulted in identification of a 662 gene signature; this signature correlated in publicly available datasets with a gene signature for IGF signaling, and in one cohort was associated with a shorter time to recurrence.[30]

Inflammation. There is also compelling evidence that obesity produces a systemic inflammatory state[31], which in some settings may promote neoplastic transformation or growth.[32] In both dietary and mouse genetic models of obesity, necrotic adipocytes surrounded by macrophages form crown-like structures (CLS) in the mammary glands and visceral fat, and are associated with activation of NF-kappaB, proinflammatory mediators, and elevated levels of aromatase.[33] Indeed, it has been shown that the relative benefit of the aromatase inhibitor (AI) anastrozole versus tamoxifen tends to be better in leaner women compared to overweight women in the ATAC trial.[34] It is conceivable therefore, that CLS may serve as biomarker for AI resistance, and such patients may require tamoxifen or higher than conventional AI dosing in order to adequately suppress the estrogen signaling.

Given the important role that each of these factors described above, a late relapse biospecimen bank will facilitate evaluation of tumor and host factors that may contribute to recurrence. Potential candidate biomarkers therefore include biomarkers reflecting occult tumor burden (plasma tumor DNA and CTCs) and host factor (possibly including but not limited to estrogens and their metabolites, inflammatory markers, and multiple components of the insulin/IGF-axis ligand and receptor levels in circulation and in tissues). Moreover, an ongoing NCI-sponsored clinical trial (MA32) is evaluating the role of metformin to prevent early recurrence within 5 years, and will attempt to identify predictive biomarkers of benefit (similar to those being evaluated in this proposal). Should that trial prove to be positive, this will provide a foundation whether metformin or other strategies may be useful in preventing late relapse.

XV.4 Comorbidities and Competing Risks in Operable Breast Cancer

Rev. 3/13

From 2004-2008, the median age at diagnosis for cancer of the breast was 61 years of age, and approximately 16% were 70 years of age or older (including 15.5% between 75 and 84 and 5.6% 85+ years of age) (http://seer.cancer.gov/statfacts/html/breast.html#references). Comorbidities are common in the elderly and pose a competing risk that attenuates the potential benefit of adjuvant therapy. For women with early stage ER-positive breast cancer, the risk of death enrolled in the ATAC trial from causes other than breast cancer at 10 years ranges from 20-60%. [35] ***Given our focus on factors contributing to late relapse, it will be critical to evaluate competing risks in this patient population who will be on average at least 5 years older than at presentation.***

XV.5 Research Plan

We propose to evaluate patients enrolled on two ECOG-coordinated adjuvant trials, including: (1) **E5103:** This trial accrued 4994 patients with node-positive (or high risk node-negative), HER2/neu negative operable breast cancer between 11/2/07 and 2/28/11. All patients received sequential doxorubicin/cyclophosphamide-paclitaxel chemotherapy alone or in combination with bevacizumab, plus endocrine therapy for patients with ER-positive disease (2) **TAILORx:** This trial enrolled 10,273 patients with ER-positive, HER2/neu negative, node negative breast cancer between 4/7/06 and 10/6/10, of whom 1629 with a Recurrence Score (RS) < 11 were assigned to endocrine therapy alone, 1737 with an RS > 25 were assigned to chemotherapy plus endocrine therapy, and 6907 were randomized to endocrine therapy vs. chemoendrocrine therapy. Consenting trial participants in both trials had banking of tumor, germ-line DNA, and serum/plasma at baseline.

Rev. 3/13

We will identify patients who meet the following criteria: (1) alive and disease-free between 4.5-7.5 years after registration in the parent trial, (2) willingness to sign informed consent and donate blood specimens annually x 5 years after registration. ***In***

addition to being followed for recurrence, second primary cancer, and survival in accordance with the parent protocol, patients will also be evaluated at 6 time points (in E5103) as described in the table below, including 1 CTC measurement at baseline. Patients who have a recurrence will be asked to provide a blood specimen and specimen from the metastatic tumor (when feasible).

Serum/plasma samples will be forwarded to the ECOG-ACRIN Central Biorepository and Pathology Facility (CBPF).

XV.5.1 Plasma Tumor DNA and Circulating Tumor Cells (CTCs).

Rev. 10/13
Rev. 1/15

CTC samples will be sent to and analyzed within 96 hours of collection in the laboratory of Dr. Katherine Alpaugh at the Fox Chase Cancer Center using CellSave tubes and the Veridex CellSearch™ platform, which allows detection of as few as 1 CTC/9 ml blood. The Alpaugh lab has considerable expertise in CTC measurement in both research and clinical settings. [36-39]The CTC portion of the laboratory is CLIA certified to perform the FDA-approved clinical diagnostic testing for metastatic breast, prostate and colon cancer and has a CTC research division exploring alternative CTC separation methods to obtain single CTC for mutational gene analysis. The laboratory has supported two previous ECOG studies (E1104 and E1105) and four GOG studies for which samples were shipped for analysis.

XV.5.2 Inflammatory Biomarkers

Inflammatory biomarkers will be evaluated using the MDS V-Plex Human Cytokine 36-Plex Kit. We hypothesize that one or more inflammatory biomarkers will be associated with distant recurrence. Rather than evaluate only specimens from patients enrolled 5 or more years after diagnosis on EL112, a case-control study (relapsers v. non-relapses) involving approximately 550 serum specimens from E5103 patients (see statistical section), will be performed:

- Baseline/registration (E5103-Step 1): N=3401 (68% of 4994 trial participants) have a baseline serum specimen in STS, of which N=250 are expected to be associated with distant recurrence (based on 729 iDFS events x 68% = 496 samples x 50% expected to have distant recurrence and usable sample. This analysis will therefore include analysis of 500 specimens (250 cases: 250 controls)
- Late relapse bioabank (E5103-Step 3): N=711, of which N=422 have a specimen from both Step 1 and Step 3, associated with 24 late distant recurrences after registration on step 3. We propose case: cohort study (relapsers:non-relapses) involving approximately 48 serum specimens (24 relapsers and 24 non-relapsers).

The V-Plex Human Cytokine 36-Plex Kit requires 0.21mL of serum, which would not deplete any specimens from any patient. The V-PLEX Human Cytokine 36-Plex is optimized for long-term and multi-site biomarker studies. The 36 assays in the V-PLEX Human Cytokine 36-Plex are provided in four multiplex panels—Chemokine Panel 1 (human), Cytokine Panel 1 (human), TH17 Panel 1 (human), and Proinflammatory Panel 1 (human). The panels are optimized to achieve the highest level of performance from each assay, providing efficient biomarker screening and profiling without compromising performance. These panels are composed of assays against human cytokines and chemokines that are involved in many biological processes such as inflammation, the Th1/Th2 pathway,

chemotaxis, the Th17 pathway, angiogenesis, and immune system regulation. As a result of their association with such a wide number of diseases, these assays are the subject of drug discovery projects, diagnostics development, and basic research. The 36-plex panel can be initially used as a screening panel to identify relevant biomarkers that can then be assembled into a custom panel for long-term studies. The panel includes the following: Eotaxin, Eotaxin-3, GM-CSF, IFN-γ, IL-10, IL-12/IL-23p40, IL-12p70, IL-13, IL-15, IL-16, IL-17A, IL-1α, IL-1β, IL-2, IL-21, IL-22, IL-23, IL-27, IL-31, IL-4, IL-5, IL-6, IL-7, IL-8, IL-8 (HA), IP-10, MCP-1, MCP-4, MDC, MIP-1α, MIP-1β, MIP-3α, TARC, TNF-α, TNF-β, VEGF-A. Additional information may be found on the link at

https://www.mesoscale.com/en/products/v-plex-human-cytokine-36-plex-kit-k15089d/

These assessments will occur under the direction of Ignacio Wistuba, MD at the MD Anderson Cancer Center.

Statistical Analysis: Cases (recurrences) will be matched to controls using a propensity score approach, with the factors hormone receptor status, number of positive nodes, tumor size, tumor grade, menopausal status, and age taken into account. Controls will also be required to have at least as much follow-up as the corresponding cases. Controls for cases in the late relapse cohort must also be in the late relapse cohort. Only one control will be assayed for each case.

Pearson and Spearman correlations among the levels of 36 cytokine markers will be computed, as will correlations between these markers and BMI and other quantitative patient and disease characteristics. Associations of the markers with categorical patient and disease characteristics, such as hormone receptor status, menopausal status and grade, will be examined using Kruskal-Wallis tests. Principal components analysis will be used to look for lower dimensional structure in the set of 36 cytokine markers.

The primary endpoint for examining association with disease outcomes is disease recurrence. Conditional logistic regression analysis, with models fit via maximum likelihood, will be used to estimate hazard ratios and test for associations (because matched controls are sampled from the risk set for the case, the odds ratio in the conditional logistic model corresponds to the hazard ratio for distant recurrence in a proportional hazards model). Tests will be performed at the two-sided 5% significance level. The power for a 1:2 matched case-control sample is approximated using formula (7) in Dupont [41]. With 250 matched sets, there will be 80% power for a 4 hazard ratio of 1.66 for comparing high vs. low values of a marker using a median split if the correlation of the marker values between cases and controls is 0. If this correlation is 0.25, then there would still be power for a hazard ratio of 1.80. For considering joint effects of the cytokines, the leading principal components will also be examined as variables in the models. Models incorporating L1 regularization (LASSO) will also be fit, to consider joint effects of multiple factors.

For the late recurrence cohort, similar analyses will be performed, but with 24 recurrences, power will be limited. With 24 recurrences, the two-sided 5% conditional logistic model test for association of recurrence with a single continuous marker will have 80% power for an effect size corresponding to a hazard ratio of 6.45 for comparing two groups with a median split if the within matched set correlation is 0.

XV.5.3 **Cell Free DNA Pilot Study**

We will isolate and quantify total cell free (cf)DNA from blood samples submitted from patients on E5103 in order to determine the feasibility of using these samples from this study for genotyping analysis. The goal is to obtain at least 20ng of cfDNA per ml (10ng are needed for genotyping NGC analysis). In addition, we propose to run a cfDNA Integrity Index which evaluates the size distribution of the cfDNA by Alu quantitative PCR (AluqPCR) that targets 300 bp long Alu repetitive sequences, which are randomly interspersed across the human genome. The expected integrity of plasma cfDNA, calculated as the ratio of AluqPCR values (Alu247/Alu115), is in the range of 0.39 to 0.55. The primary purpose here is to determine feasibility of isolating cf DNA prior to largescale evaluation for specific circulating tumor DNA assays. We propose that this be done from 25 samples collected using PST-heparin tube, and 25 specimens obtained in PPT-EDTA tube from the same 25 patients; specimens would be selected from subjects who have not had recurrence and had limited/missing followup information (as selected by the statistical center), and thus would otherwise be of little value. This analysis would be done under Dr. Wistuba's supervision at MD Anderson, using methods previously described by his group [42]. It is noteworthy that a key finding from this publication was that the mean cfDNA from EDTA tubes processed is a similar manner within 2 hours of blood draw was approximately 25 ng DNA/ml plasma.

Statistical Analysis: For cfDNA extraction, the primary endpoint is the proportion of samples where adequate cfDNA for analysis can be extracted from each type of sample. With 25 samples for each type, the exact binomial 95% confidence interval on the true rate is (80%, 100%) if 24/25 are adequate, (74%,99%) if 23/25 are, (69%, 97%) if 22/25 are, and (59%, 93%) if 20/25 are.

XV.6 **Patient Participation Requirements for Ancillary Study EL112LAB**

Rev. 3/13

The ECOG-ACRIN Operations Office - Boston will provide sites a list of patients who have been identified as potential candidates for EL112LAB. The information provided will include the date range for which the patients would be eligible to participate which is dependent on the date of randomization to treatment. Routine notifiers will also be utililized to serve as reminders to sites regarding the timeline of a patient's potential eligibility.

Rev. 10/13

The list of **potentially eligible** patients at your site will be provided via a password protected WebPortal. The location and access information to the WebPortal:

web link: https://webapps.ecog.org/LabStudyEligible/

web name: EL112LAB Potential Eligible Patient List

You access the WebPortal using your CTSU login and password. Access to the listing will be based on your site association assignment in RSS, thus it is important that the sites' contacts are up to date with CTEP.

The listing of eligible patient will be rolling, being updated as new patients enter the eligibility window. The list is based on time of registration to treatment on the respective parent trial, are alive and recurrence free, and have not withdrawn participation from follow-up on the respective trial as reported to ECOG-ACRIN or the CTSU on the relevant study forms. Full eligibility requirements are outlined below. Actual patient

eligibility is to be verified by the site prior to registration to EL112LAB and collection of the EL112LAB blood specimens.

XV.6.1 Patient Eligibility

Patients must meet the following criteria:

Rev. 3/13

1. Patient was registered to treatment on ECOG-ACRIN trials E5103 or PACT1/ TAILORx, at least 4.5 years (54 months) and no more than 7.5 years (90 months) prior to registration to step 3. It is preferable for patients to be registered to step 3 at 5 years (60 months +/- 6 months) after registration to treatment on the relevant clinical trial.
2. Patient is disease free, with no prior recurrence, at time of registration to EL112LAB.
3. Patient must be willing to contribute the required information and specimens.
4. Previous submission of tumor tissue or blood specimens:
    a. E5103 patients – If primary tumor specimens had not been submitted after patient registered to treatment per E5103 Section 10, tumor tissue must be available for submission within 4 weeks following registration to Step 3
    b. PACT1/TAILORx - The primary tumor tissue and peripheral blood samples were previously submitted for research studies just prior to or following registration to treatment as outlined in PACT1/TAILORx Section 10.
5. Patient must provide written signed consent to participate in the Ancillary Substudy EL112LAB: *North American Breast Cancer Groups Biospecimen Bank for Determinants of Late Relapse in Operable Breast Cancer.* The model consent for the substudy is provided as Appendix I-A of the relevant parent clinical trial.

XV.6.2 Registration to the Ancillary Study EL112LAB

Patients are registered to Step 3 as outlined in Section 4 of the parent protocol (E5103, Section 4.11; PACCT-1/TAILORx Section 4.4).

Rev. 10/13

Regarding timing of Registration to EL112LAB, blood collection and sample submission:

It is understood that blood collection may immediately follow obtaining the patient's consent to participate in EL112LAB, thus registration to EL112LAB may follow the blood collection. If this occurs, register the patient to step 3 within 48 hours following the blood collection. Step 3 registration is required to update the consent permission for the specimen collection and use. Note that STS will allow logging the samples prior to registration to step 3 and report the actual date of blood collection.

If the blood collection is delayed more than 1 week following time of consent, registration to EL112LAB (step 3 registration) is to be delayed until just prior to the blood draw. Expectancy timepoints are tagged from date of registration, not time of actual collection. A long time frame between registration to EL112LAB

and the initial EL112LAB blood draw will result in expectancy tracking problems for the site and the lab.

XV.6.3 Data and Specimen Submissions

NOTE: Requirements for EL112LAB do NOT impact or change the requirements of the parent protocol.

A. Comorbidity, BMI and Menstrual History

Rev. 3/13 EL112LAB Comorbidity Form is to be completed at time of registration to EL112LAB.

Rev. 3/13 The EL112LAB Menstrual History Form is to be completed at the following time points:

- Registration to EL112LAB
- Years 1,2,3,4,5 from time of registration to EL112LAB

Please refer to the Forms Packet of the relevant parent clinical trial for copies of the forms. The E5103 or PACT1/TAILORx Forms Packet may be downloaded by accessing the ECOG World Wide Web Home Page (http://www.ecog.org) or from the CTSU website. Forms must be submitted to the ECOG-ACRIN Operations Office - Boston (ATTN: DATA) at FSTRF, 900 Commonwealth Avenue, Boston, MA 02215.

B. Specimen Submissions

To order the blood collection/submission kits, follow the instructions in Appendix XIV.

Rev. 10/13 It is encouraged that blood collections to occur at the time the patient attends the clinic for their standard or follow-up appointments to monitor their health. Trips to the clinic specifically for collection of these research samples are not required. If draw is not a fasting draw, report the date and time of last caloric intake in STS.

All sample submissions must be logged and tracked using the **ECOG-ACRIN Sample Tracking System (STS)**. The STS generated shipping manifest is to be included with all shipments. If STS is unavailable at time of sample submission, follow the instructions in section 10.4 of E5103 or PACT1/TAILORx.

Rev. 1/15 **Access to the shipping account for specimen shipments** for this project can now only be obtained by logging into fedex.com with an account issued by the ECOG-ACRIN CBPF. For security reasons, the account number will no longer be given out in protocols, over the phone, or via email. If your site needs to have an account created, please contact the ECOG-ACRIN CBPF by email at eacbpf@mdanderson.org.

Specimen collection and submission requirements are summarized in Table 2. Collection tubes are listed in order of recommended draw order, however follow your institutional draw order requirements.

All specimens submitted must be labeled with the parent protocol number, the ECOG-ACRIN protocol specific patient ID, patient initials, date of collection and sample type (e.g. PST, RST, PPT, CTAD, Immunex).

Table XV.1 Specimen Submission Summary

Specimen Type	Collection type	Protocol: Time points	Submit to:
Plasma[1,2] + residual cells (WBC+RBC)	CTAD (sodium citrate, theophylline, adenosine, dipyridamole;light blue top)	E5103<br>• Registration to EL112LAB<br>• Years 1,2,3,4,5<br>• Recurrence<br>PACT1/TAILORx<br>• Registration to EL112LAB<br>• Recurrence	ECOG-ACRIN Central Biorepository and Pathology Facility (CBPF)
Rev. 1/15 Serum[1,2]	Rapid Serum Tube (RST – no anticoagulant, orange top)		
Plasma[1,2]	Plasma Separator Tube (PST – lithium heparin, green top),		
Plasma[1,2]	Plasma Preparation Tube (PPT - EDTA, pearl white top)		
Peripheral Blood[1]	CellSave®	E5103<br>• Registration to EL112LAB	Fox Chase Cancer Center Clinical Protocol Support Lab, P2011
Rev. 10/13 Rev. 1/15 Tumor Tissue Block	Primary Tumor	E5103<br>Required if not previously submitted	ECOG-ACRIN Central Biorepository and Pathology Facility (CBPF)
	Recurrence, diagnostic biopsy	All patients	

1. FASTING DRAWS STRONGLY ENCOURAGED. Date and time of specimen collection and last caloric intake is to be provided, via STS, with submission of the blood specimens.

Rev. 10/13

2. If a kit is not available at time of sample collection, institutional supplies may be utilized. If the equivalent tube types cannot be obtained, the following standard tubes may be substituted: RST = SST or red top, PST = heparin (green top) tube, PPT = EDTA (purple top) tube, CTAD = citrate (light blue top). For E5103 patients, there are no substitutions for CellSave® vacutainers.

I. Submission to the FCCC Clinical Protocol Support Laboratory (LAB015)

Rev. 10/13, 10/14

Peripheral Blood (Immunicon Cell Save Tubes): Draw 9mL of blood into each tube, making sure to fill the tube completely. Sample must be shipped *day of collection* at ambient temperature. DO NOT REFRIGERATE SAMPLE

FASTING DRAWS ARE STRONGLY ENCOURAGED. Provide date and time of last caloric intake in STS.

Samples must be sent by overnight courier at ambient temperature on the *day of collection* to address below. Questions may be directed to Kathy Alpaugh at (215) 214-1634 or email (rk_alpaugh@fccc.edu). Samples must be sent to arrive Monday through Friday. The laboratory is CLOSED on Saturdays, Sundays and holidays. IF DRAWN ON A FRIDAY mark the waybill “FOR MONDAY DELIVERY”. If to be drawn on a DAY BEFORE A HOLIDAY, telephone the Fox Chase Laboratory to receive alternative shipping instructions.

Ship Immunicon tubes *only* to:

Fox Chase Cancer Center
Clinical Protocol Support Lab, P2011
Attn: Kathy Alpaugh
333 Cottman Avenue
Philadelphia, PA 19111-2497

Rev. 1/15

II. Submissions to the ECOG-ACRIN Central Biorepository and Pathology Facility (CBPF)

Questions are to be directed to the ECOG-ACRIN CBPF at eacbpf@mdanderson.org or call 844-744-2420.

a. Plasma and Serum Samples

- FASTING DRAWS STRONGLY ENCOURAGED. Provide date and time of last caloric intake in STS.
- Peripheral blood should be collected into each vacutainer.
- Immediately invert tube several times: CTAD, 3-4 times; RST, 5-6 times; PPT and PST, 8-10 times.
- For the RST tube, allow to clot for at least 5 minutes. If an SST or standard red top tube is used, allow to clot for 30 minutes.
- Centrifuge, within two hours of draw, at room temperature at 1100g – 1300g for 10 minutes in a swing bucket or 15 minutes for a fixed angle centrifuge
- Aliquot serum and plasma into 2 cryovials for each vacutainer collected. CLEARLY LABEL CRYOTUBES WITH RST, PPT, PST or CTAD or institutional tube types utilized.
- For the CTAD vacutainer – DO NOT DISCARD RESIDUAL CELLS. Restore the cap to the plastic vacutainer tube and store with the plasma and serum samples. If the vacutainer is glass, transfer cells to a vryovial prior to freezing.
- Freeze cryovials and residual cells at ≤–70ºC. Batch ship overnight on dry ice. If speciments cannot be stored at ≤-70 ºC, store at ≤-20ºC and ship within one (1) week of collection.

b. Tumor tissue specimens

The required representative tumor blocks (see table XV.1) are to be submitted within 4 weeks of registration to EL112LAB (primary tumor) or collection (recurrence.) If a block is not available, contact the ECOG-
Rev. 1/15
ACRIN CBPF at (844) 744-2420 to discuss possible alternative submission guidelines.

The following forms are to be submitted with the tumor tissue:

- STS-generated shipping manifest
- Copy of the Pathology Report

Samples are to be shipped Monday through Thursday only. Pathology materials are to be submitted at ambient within one month of collection.

Plasma and serum samples are to be shipped on dry ice quarterly. Multiple patient samples may be batched shipped together, however each plastic bag must contain samples from a single patient only.

Ship with the relevant documentation to:

ECOG-ACRIN Central Biorepository and Pathology Facility
MD Anderson Cancer Center
Department of Pathology, Unit 085
Tissue Qualification Laboratory for ECOG-ACRIN, Room G1.3586
1515 Holcombe Blvd
Houston, TX 77030
Phone: Toll Free 844-744-2420 (713-745-4440 Local or International Sites)
Fax: 713-563-6506
Rev. 1/15 Email: eacbpf@mdanderson.org

XV.7 References

1. Davies C, Godwin J, Gray R, et al: Relevance of breast cancer hormone receptors and other factors to the efficacy of adjuvant tamoxifen: patient-level meta-analysis of randomised trials. Lancet 378:771-84, 2011
2. Peto R, Davies C, Godwin J, et al: Comparisons between different polychemotherapy regimens for early breast cancer: meta-analyses of long-term outcome among 100,000 women in 123 randomised trials. Lancet 379:432-44, 2012
3. Sparano JA, Wang M, Martino S, et al: Weekly paclitaxel in the adjuvant treatment of breast cancer. The New England journal of medicine 358:1663-71, 2008
4. Sparano JA, Strickler HD: Breast cancer patients who are obese at diagnosis: alea iacta est? or "is the die cast"? Oncology 25:1002, 1004, 1007, 2011
5. de Azambuja E, McCaskill-Stevens W, Francis P, et al: The effect of body mass index on overall and disease-free survival in node-positive breast cancer patients treated with docetaxel and doxorubicin-containing adjuvant chemotherapy: the experience of the BIG 02-98 trial. Breast Cancer Res Treat 119:145-53, 2010
6. Majed B, Moreau T, Senouci K, et al: Is obesity an independent prognosis factor in woman breast cancer? Breast Cancer Res Treat 111:329-42, 2008
7. Sparano JA, Paik S: Development of the 21-gene assay and its application in clinical practice and clinical trials. Journal of clinical oncology : official journal of the American Society of Clinical Oncology 26:721-8, 2008
8. Mook S, Schmidt MK, Weigelt B, et al: The 70-gene prognosis signature predicts early metastasis in breast cancer patients between 55 and 70 years of age. Annals of oncology : official journal of the European Society for Medical Oncology / ESMO 21:717-22, 2010
9. Jin H, Tu D, Zhao N, et al: Longer-term outcomes of letrozole versus placebo after 5 years of tamoxifen in the NCIC CTG MA.17 trial: analyses adjusting for treatment crossover. Journal of clinical oncology : official journal of the American Society of Clinical Oncology 30:718-21, 2012
10. Shaw JA, Page K, Blighe K, et al: Genomic analysis of circulating cell-free DNA infers breast cancer dormancy. Genome research 22:220-31, 2012

11. Lucci A, Hall CS, Lodhi AK, et al: Circulating tumour cells in non-metastatic breast cancer: a prospective study. The lancet oncology, 2012
12. Kabat GC, Kim M, Caan BJ, et al: Repeated measures of serum glucose and insulin in relation to postmenopausal breast cancer. Int J Cancer 125:2704-10, 2009
13. Goodwin PJ, Ennis M, Pritchard KI, et al: Fasting insulin and outcome in early-stage breast cancer: results of a prospective cohort study. J Clin Oncol 20:42-51, 2002
14. Duggan C, Irwin ML, Xiao L, et al: Associations of insulin resistance and adiponectin with mortality in women with breast cancer. J Clin Oncol 29:32-9
15. Irwin ML, Duggan C, Wang CY, et al: Fasting C-peptide levels and death resulting from all causes and breast cancer: the health, eating, activity, and lifestyle study. J Clin Oncol 29:47-53
16. Wildman RP: Healthy obesity. Curr Opin Clin Nutr Metab Care 12:438-43, 2009
17. Milazzo G, Giorgino F, Damante G, et al: Insulin receptor expression and function in human breast cancer cell lines. Cancer Res 52:3924-30, 1992
18. Frittitta L, Vigneri R, Papa V, et al: Structural and functional studies of insulin receptors in human breast cancer. Breast Cancer Res Treat 25:73-82, 1993
19. Pugeat M, Crave JC, Elmidani M, et al: Pathophysiology of sex hormone binding globulin (SHBG): relation to insulin. J Steroid Biochem Mol Biol 40:841-9, 1991
20. Barbieri RL, Smith S, Ryan KJ: The role of hyperinsulinemia in the pathogenesis of ovarian hyperandrogenism. Fertil Steril 50:197-212, 1988
21. Panno ML, Salerno M, Pezzi V, et al: Effect of oestradiol and insulin on the proliferative pattern and on oestrogen and progesterone receptor contents in MCF-7 cells. J Cancer Res Clin Oncol 122:745-9, 1996
22. Elias JJ, Armstrong RC: Brief communication: Hyperplastic and metaplastic responses of human mammary fibroadenomas and dysplasias in organ culture. J Natl Cancer Inst 51:1341-3, 1973
23. Law JH, Habibi G, Hu K, et al: Phosphorylated insulin-like growth factor-i/insulin receptor is present in all breast cancer subtypes and is related to poor survival. Cancer Res 68:10238-46, 2008
24. Huang GS, Brouwer-Visser J, Ramirez MJ, et al: Insulin-like growth factor 2 expression modulates Taxol resistance and is a candidate biomarker for reduced disease-free survival in ovarian cancer. Clin Cancer Res 16:2999-3010, 2010
25. Duggan C, Irwin ML, Xiao L, et al: Associations of insulin resistance and adiponectin with mortality in women with breast cancer. Journal of clinical oncology : official journal of the American Society of Clinical Oncology 29:32-9, 2011
26. Matthews DR, Hosker JP, Rudenski AS, et al: Homeostasis model assessment: insulin resistance and beta-cell function from fasting plasma glucose and insulin concentrations in man. Diabetologia 28:412-9, 1985
27. Erickson K, Patterson RE, Flatt SW, et al: Clinically defined type 2 diabetes mellitus and prognosis in early-stage breast cancer. Journal of clinical oncology : official journal of the American Society of Clinical Oncology 29:54-60, 2011

28. Peairs KS, Barone BB, Snyder CF, et al: Diabetes mellitus and breast cancer outcomes: a systematic review and meta-analysis. Journal of clinical oncology : official journal of the American Society of Clinical Oncology 29:40-6, 2011
29. Sparano JA, Gray, R., Goldstein L.J., Childs, B.H., Brassard, D., Bugarini, R., Rowley, S., Baker, J., Shak, S., Badve, S., Baehner, F.L., Kenny, P., Perez, E.A., Shulman, L.N., Martino, S., Sledge, G.W., Davidson, N.E.: Gene Expression Profiling of Phenotypically-Defined Hormone-Receptor Positive Breast Cancer: Evidence for Increased Transcriptional Activity of the Insulin Growth Factor Receptor Pathway and Other Pathways. Breast Cancer Res Treat (abstract 5165), 2009
30. Creighton CJ, Sada YH, Zhang Y, et al: A gene transcription signature of obesity in breast cancer. Breast Cancer Res Treat, 2011
31. Sinicrope FA, Dannenberg AJ: Obesity and breast cancer prognosis: weight of the evidence. J Clin Oncol 29:4-7, 2011
32. Wang T, Rohan TE, Gunter MJ, et al: A prospective study of inflammation markers and endometrial cancer risk in postmenopausal hormone nonusers. Cancer Epidemiol Biomarkers Prev 20:971-7, 2011
33. Subbaramaiah K, Howe LR, Bhardwaj P, et al: Obesity is associated with inflammation and elevated aromatase expression in the mouse mammary gland. Cancer Prev Res (Phila) 4:329-46, 2011
34. Sestak I, Distler W, Forbes JF, et al: Effect of body mass index on recurrences in tamoxifen and anastrozole treated women: an exploratory analysis from the ATAC trial. J Clin Oncol 28:3411-5, 2010
35. Ring A, Sestak I, Baum M, et al: Influence of comorbidities and age on risk of death without recurrence: a retrospective analysis of the arimidex, tamoxifen alone or in combination trial. Journal of clinical oncology : official journal of the American Society of Clinical Oncology 29:4266-72, 2011
36. Behbakht K, Sill MW, Darcy KM, et al: Phase II trial of the mTOR inhibitor, temsirolimus and evaluation of circulating tumor cells and tumor biomarkers in persistent and recurrent epithelial ovarian and primary peritoneal malignancies: a Gynecologic Oncology Group study. Gynecol Oncol 123:19-26, 2011
37. Cohen SJ, Alpaugh RK, Gross S, et al: Isolation and characterization of circulating tumor cells in patients with metastatic colorectal cancer. Clin Colorectal Cancer 6:125-32, 2006
38. Urtishak S, Alpaugh RK, Weiner LM, et al: Clinical utility of circulating tumor cells: a role for monitoring response to therapy and drug development. Biomark Med 2:137-45, 2008
39. Dotan E, Cohen SJ, Alpaugh KR, et al: Circulating tumor cells: evolving evidence and future challenges. Oncologist 14:1070-82, 2009
40. https://www.mesoscale.com/en/products/v-plex-human-cytokine-36-plex-kit-k15089d/
41. Dupont, WD. Power calculations for matched case-control studies. Biometrics 1988; 44(4):1157-1168.
42. Mehrotra M, Singh RR, Chen W, et al. Study of preanalytic and analytic variables for clinical next generation sequencing of circulating cell-free nucleic acid. J Mol Diagn. 2017 Jul;19(4):514-524.
