# Supplementary material for: Quality of Life Following Receipt of Adjuvant Chemotherapy With and Without Bevacizumab in Patients With Lymph Node–Positive and High-Risk Lymph Node–Negative Breast Cancer
Source: JAMA Netw Open. 2022 Feb 28;5(2):e220254. doi: 10.1001/jamanetworkopen.2022.0254 (PMC8886546; doi:10.1001/jamanetworkopen.2022.0254)
Supplement: Supplement 2. — eFigure. E5103 Decision-making–Quality of Life Flowchart eTable. Comparison of Baseline Characteristics Between Those Included and Not Included in the QOL Analysis Relative to the Parent Trial [file jamanetwopen-e220254-s002.pdf]

Supplemental Online Content

Rosenberg SM, O'Neill A, Sepucha K, et al. Quality of life following receipt of adjuvant chemotherapy with and without bevacizumab in patients with lymph node–positive and high-risk lymph node–negative breast cancer. *JAMA Netw Open.* 2022;5(2):e220254. doi:10.1001/jamanetworkopen.2022.0254

eFigure. E5103 Decision-making–Quality of Life Flowchart

eTable. Comparison of Baseline Characteristics Between Those Included and Not Included in the QOL Analysis Relative to the Parent Trial

This supplemental material has been provided by the authors to give readers additional information about their work.

© 2022 Rosenberg SM et al. *JAMA Network Open.*

eFigure. E5103 Decision-making–Quality of Life Flowchart

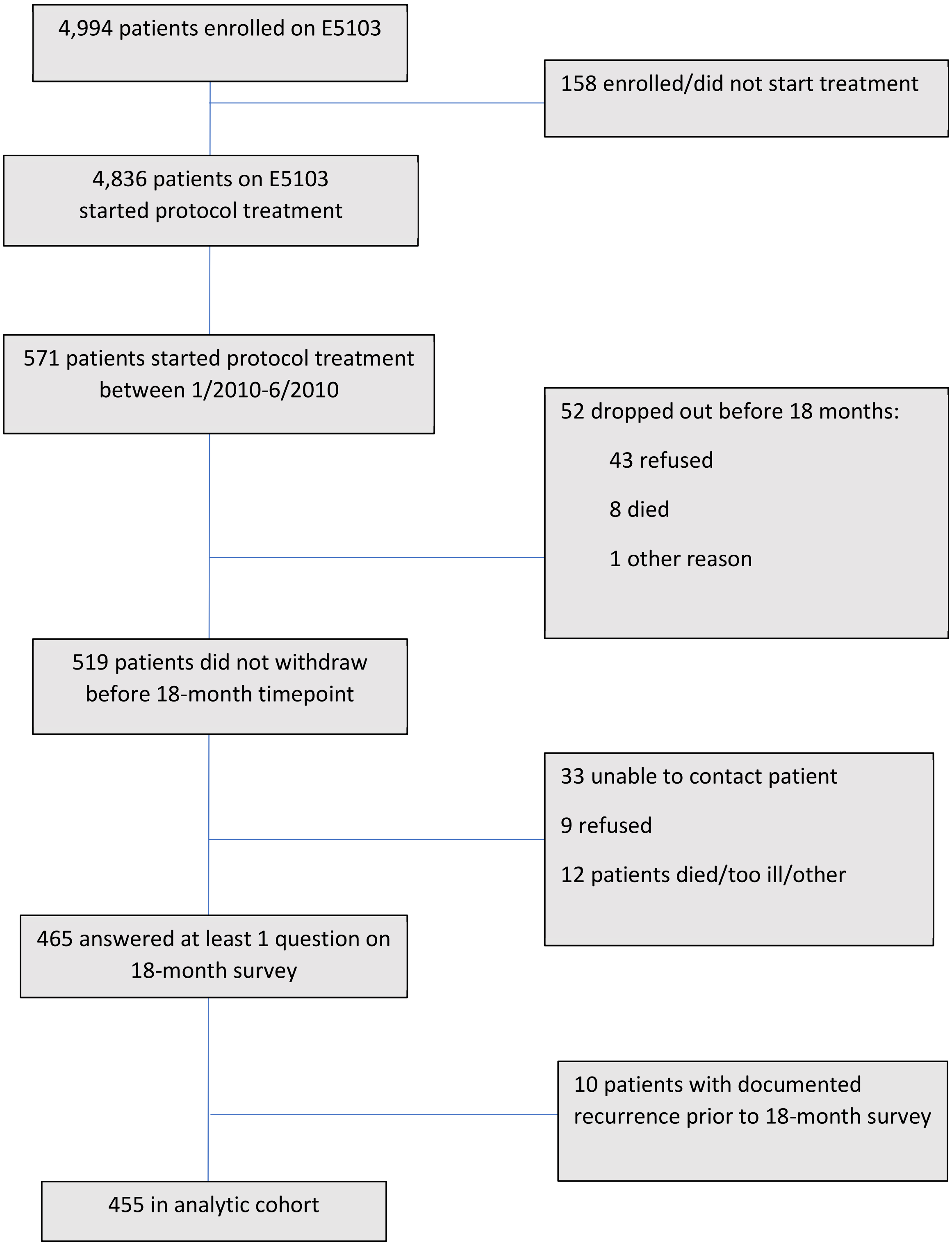

© 2022 Rosenberg SM et al. *JAMA Network Open.*

eTable. Comparison of Baseline Characteristics Between Those Included and Not Included in the QOL Analysis Relative to the Parent Trial

	Parent Trial not in QOL<br>N=4381	In QOL<br>N=455	Total<br>N=4836*
	n (%)	n (%)	n (%)
Gender			
Male	19(<1)	3(<1)	22
Female	4362(99.6)	452(99.3)	4814
Age			
Median	51.6	51.9	51.6
Race			
White	3702(85)	393(86)	4095
Other	679(15)	62(14)	741
Ethnicity			
Hispanic	318(8)	23(5)	341
Non-Hispanic	3867(92)	403(95)	4270
Missing/unknown			225
Primary Surgery/RT			
Breast Conserving Surgery	1968(46)	195(43)	2163
Mastectomy no WBRT	870(20)	86(19)	956
Mastectomy +WBRT	1451(34)	171(38)	1622
Missing			95
ERPgR Status			
ER and PgR Negative	1588(36)	156(34)	1744
ER and/or PgR Positive	2791(64)	299(66)	3090
Missing			2
Tumor Size			
≤2 cm	1703(39)	165(36)	1868
>2	2764(61)	290(64)	2964
Missing			4

© 2022 Rosenberg SM et al. *JAMA Network Open.*

	Parent Trial not in QOL<br>N=4381	In QOL<br>N=455	Total<br>N=4836*
	n (%)	n (%)	n (%)
Nodal Status			
Negative	1193(27)	125(27)	1318
Positive	3186(73)	330(73)	3516
Missing			2
Histologic Grade			
I	375(9)	39(9)	414
II	1412(33)	160(36)	1572
III	2498(58)	245(55)	2743
Missing			107

*n=4386 who started protocol treatment

© 2022 Rosenberg SM et al. *JAMA Network Open.*
