# Supplementary material for: Quality of Life Following Receipt of Adjuvant Chemotherapy With and Without Bevacizumab in Patients With Lymph Node–Positive and High-Risk Lymph Node–Negative Breast Cancer
Source: JAMA Netw Open. 2022 Feb 28;5(2):e220254. doi: 10.1001/jamanetworkopen.2022.0254 (PMC8886546; doi:10.1001/jamanetworkopen.2022.0254)
Supplement: Supplement 3. — Data Sharing Statement [file jamanetwopen-e220254-s003.pdf]

Data Sharing Statement

Rosenberg. Quality of Life Following Receipt of Adjuvant Chemotherapy With and Without Bevacizumab in Patients With Lymph Node-Positive and High-Risk Lymph Node-Negative Breast Cancer. *JAMA Netw Open*. Published February 28, 2022. doi:10.1001/jamanetworkopen.2022.0254

Data
Data available: Yes
Data types: Other (please specify)
Additional Information: All data to replicate the analyses in the article would be made available upon request.
How to access data: The data from the present article would be made available by request from the NCTN/NCORP Data Archive in a timeframe of at least 6 months after the request is made.
When available: With publication

Supporting Documents
Document types: None

Additional Information
Who can access the data: Anyone requesting the data via the NCTN/NCORP Data Archive (https://nctn-data-archive.nci.nih.gov).
Types of analyses: Investigators requesting the data outline a purpose through a data request mechanism
Mechanisms of data availability: The NCTN/NCORP Data Archive has an approval mechanism for the data request.
